# Design, synthesis and antimycobacterial activity of imidazo[1,5-*a*]quinolines and their zinc-complexes†

**DOI:** 10.1039/d4md00086b

**Published:** 2024-03-12

**Authors:** Michael Marner, Niclas Kulhanek, Johanna Eichberg, Kornelia Hardes, Michael Dal Molin, Jan Rybniker, Michael Kirchner, Till F. Schäberle, Richard Göttlich

**Affiliations:** a Fraunhofer-Institute for Molecular Biology and Applied Ecology (IME) Branch Bioresources Ohlebergsweg 12 35392 Giessen Germany; b Institute of Organic Chemistry, Justus-Liebig-University Heinrich-Buff-Ring 17 Giessen 35392 Germany; c Institute for Insect Biotechnology, Justus-Liebig-University Giessen Ohlebergsweg 12 35392 Giessen Germany; d German Center for Infection Research (DZIF), Partner Site Giessen-Marburg-Langen Ohlebergsweg 12 35392 Giessen Germany; e Department I of Internal Medicine, University of Cologne 50937 Cologne Germany; f Center for Molecular Medicine Cologne (CMMC), University of Cologne 50931 Cologne Germany; g German Center for Infection Research (DZIF), Partner Site Bonn-Cologne Cologne Germany; h BMBF Junior Research Group in Infection Research “ASCRIBE”, Branch for Bioresources of the Fraunhofer Institute for Molecular Biology and Applied Ecology IME Ohlebergsweg 12 35392 Giessen Germany

## Abstract

Tuberculosis has remained one of the world's deadliest infectious diseases. The complexity and numerous adverse effects of current treatment options as well as the emergence of multi-drug resistant *M. tuberculosis* (Mtb) demand research and innovation efforts to yield new anti-mycobacterial agents. In this study, we synthesized a series of imidazo[1,5-*a*]quinolines, including 4 new analogs, and evaluated their activity against Mtb. Inspired by previous studies, we also designed 8 compounds featuring a coordinated metal ion, determined their absolute configuration by single-crystal X-ray diffraction and included them in the bioactivity study. Remarkably, the metal complexation of 5c with either Zn^2+^ or Fe^2+^ increased the Mtb inhibitory activity of the compound 12.5-fold and reduced its cytotoxicity. Ultimately, out of the 21 analyzed imidazo[1,5-*a*]quinoline analogs, two zinc complexes (C1 and C7) showed the strongest, specific activity against Mtb H37Rv *in vitro* (IC_90_ = 7.7 and 17.7 μM).

## Abstract 2

In this study, we report the novel bioactivity of imidazo[1,5-*a*]quinolines and their complexes against *Mycobacterium tuberculosis* Mtb.

## Graphical abstract

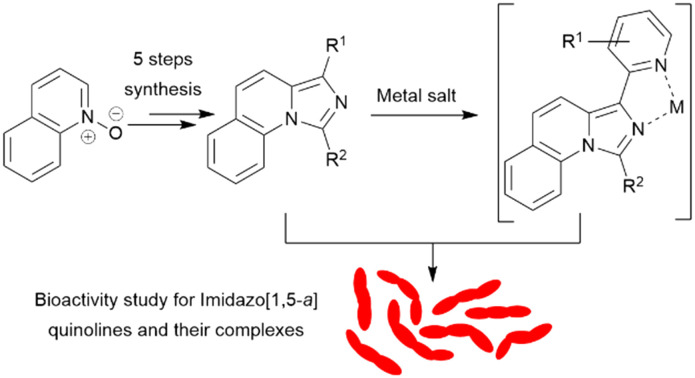

## Introduction

Tuberculosis (TB) is a communicable infectious disease caused by *Mycobacterium tuberculosis* (Mtb). Despite global efforts, TB remains one of the world's deadliest killers of the past two decades.^1–3^ In 2023, the World Health Organization (WHO) reported 10.6 million new cases and 1.3 million deaths caused by TB.^4^ In addition, the COVID-19 pandemic is considered to have erased the progress made in the years up to 2019.^5,6^ The net reduction of the TB incidence from 2015 to 2022 was only 8.7%, missing the important key milestone of the WHO *End TB Strategy* by far (50% reduction until 2025).

Recommended treatment regimens for drug-sensitive Mtb are long and complex (high doses of 4 antibiotics over 4 to 6 months or longer).^7,8^ Non-compliance, *e.g.* misused or mismanaged antibiotic therapy, facilitates the emergence and spread of rifampicin-resistant TB, multidrug-resistant TB and even extensively drug-resistant TB (RR-TB/MDR-TB/XDR-TB). In 2021, the number of MDR-TB cases increased to 450 000. Chemotherapy against MDR-TB and XDR-TB is even more complicated, and the clinical outcome is generally poor.^5^ Therefore, continuous research and innovation towards new and improved TB active agents is of great importance. A promising approach to improve the potency of anti-mycobacterial agents is their combination with further antimicrobial molecules.

Transition metals, such as zinc (Zn^2+^), are involved in many physiological processes and are appreciated for their pharmaceutical potential. As part of the innate immune response towards pathogens, macrophages can deploy phagosomal zinc intoxication defence mechanisms.^9^ Antibacterial,^10–12^ antifungal^13^ and antiparasitic^14,15^ features of metal-drug complexes have been observed in many studies.

Sonawane *et al.* demonstrated that the activity of rifampicin could be increased by complexing it with Zn^2+^ and encapsulating it into transferrin-conjugated silver quantum dots.^16^ In addition, other metal complexes with Cu^2+^ and V^5+/4+^ showed good anti-mycobacterial activity, highlighting the potential use of complexes in TB treatment.^17,18^

In this early discovery study, we set out to design, synthesize and evaluate the *in vitro* activity of imidazo[1,5-*a*]quinolines. Compounds featuring this core motif have already been reported to exhibit diverse biological activities.^19–21^ However, these scaffolds were never evaluated for their potency towards Mtb. Hence, in the first step, we expanded a set of literature-known imidazo[1,5-*a*]quinolines (5a–i)^22^ with four new structures (6a, 6b, 7a, and 8a) by diversifying substitutions at R^1^ and R^2^. The substitution of the imidazole ring was selected based on its synthetic feasibility and reaction yield. Next, we prepared metal complexes of the most intriguing compounds and evaluated their antimycobacterial activity.

## Results and discussion

### Synthesis and crystal structures

**Scheme 1:**
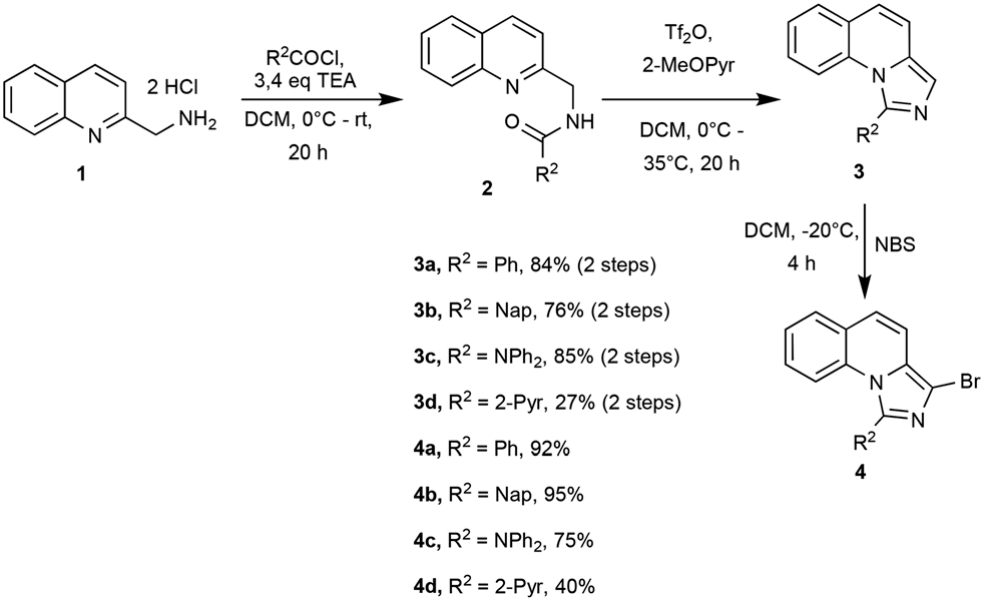
General synthesis route and isolated yields.

We employed our versatile synthetic route reported in an earlier publication (Scheme 1).^22^ Here, we introduced R^2^ over an Einhorn acylation and established the imidazo ring system by nucleophilic substitution. 3a–d was then selectively brominated with *N*-bromosuccinimide at −20 °C.

**Scheme 2:**
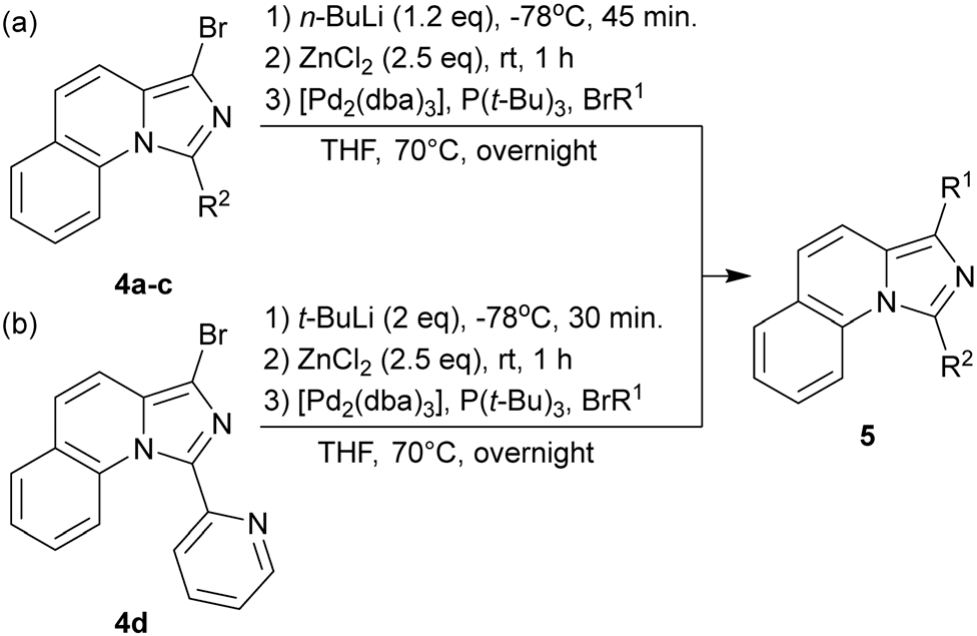
Employed coupling strategies for the final products.

Bromides (4a–c) were transmetalated with *n*-BuLi and ZnCl_2_ by Negishi coupling (Scheme 2a). The excess of ZnCl_2_ in the solution led to the formation of bidentate Zn complexes. The transmetalation with *n*-BuLi resulted in the alkylation of the nitrogen within the pyridine moity of 4d, forming *n*-butylbromide. We eliminated the presence of halogenoalkanes in the solution using 2 eq. of *t*-BuLi (Scheme 2b).

The majority of products were isolated in good yields of 58–84%. 6a–b and 5h were isolated in lower yields even with the improved reaction conditions. We observed a high percentage of 3d – an effect that we could not prevent even by longer reaction time or elevated temperature. The ligand character of 3 might have inhibited the activity of the Pd-catalyst. Similarly, the +M-substituent in the R^1^ position of 5h might have resulted in a deactivating chelating effect. 5e–5g was converted into their hydrochloride salts to improve their solubility in water (Table 1).

**Table 1:** Yields for Negishi coupling

Number	R^2^	R^1^	Isolated product/%
5a^a^	Ph	2-Thiophenyl	58
5b^a^	Ph	Ph	84
5c^a^	Ph	2-Pyridinyl	73
5d^a^	Ph	2-Quinolinyl	79
5e^b^	Ph	2-Pyrimidinyl	60
5f^b^	Ph	2-(5-Ph-Pyridinyl)	70
5g^b^	Ph	3-Isoquinolinyl	79
5h^a^	Ph	2-Me_2_N-Ph	40
5i^a^	Ph	2-MeO-Ph	84
6a^c^	2-Pyridyl	2-Pyridinyl	26
6b^c^	2-Pyridyl	Ph	39
7a^a^	Nap	2-Pyridinyl	68
8a^a^	NPh_2_	2-Pyridinyl	66

a Conditions shown in Scheme 2a.

b conditions shown in Scheme 2a, products were isolated as hydrochloride salts.

c conditions shown in Scheme 2b.

**Scheme 3:**
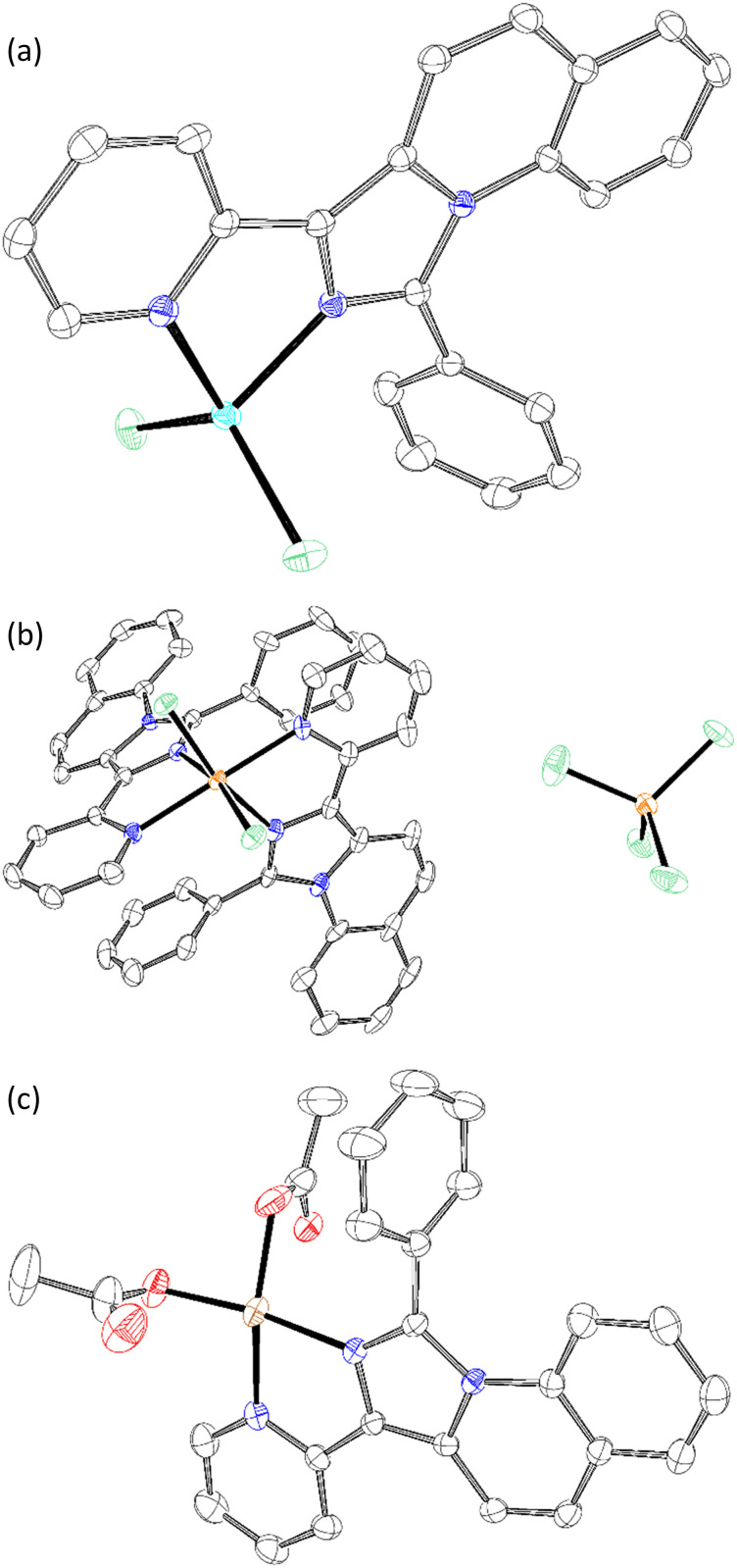
Crystal structures for (a) C1, (b) C2, and (c) C3. Analysed by XRD measurements, solved with ShelXL and visualized with ORTEP-3. Hydrogen atoms are hidden for better visibility (white = carbon, blue = nitrogen, red = oxygen, green = chlorine, cyan = zinc, orange = iron and brown = copper).

Chelation was performed with the listed ligands (Table 2) in THF. Besides Zn, compound 5c was also combined with FeCl_3_ and Cu(OAc)_2_. The structures of C1 (Scheme 3a), C2 (Scheme 3b) and C3 (Scheme 3c) were analysed by XRD from single crystals. The M^2+^-central atoms showed square planar orientation. C2 formed an octahedral complex by the displacement of chloride, resulting in a charged complex and, thus, good solvability in water. However, the solubility of the complexes also influenced the isolation yield. The most soluble complexes (C4–8) were retrieved in poor yields after the washing step. The data evaluation is provided in the supplementary information.

**Table 2:** General procedure of the complexation reaction and molecular formula of synthesised complexes with yields

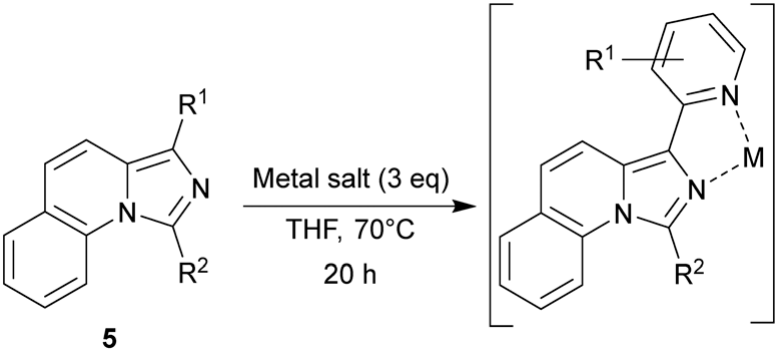				
Number	Ligand	Metal salt	Molecular formular	Isolated product/%
C1	5c	ZnCl_2_	[ZnCl_2_5c]	95
C2	5c	FeCl_3_	[FeCl_2_(5c)_2_]FeCl_4_	97
C3	5c	Cu(OAc)_2_	[Cu(OAc)_2_5c]	98
C4	7a	ZnCl_2_	[ZnCl_2_7a]	44
C5	5e	ZnCl_2_	[ZnCl_2_5e]	40
C6	5f	ZnCl_2_	[ZnCl_2_5f]	64
C7	5g	ZnCl_2_	[ZnCl_2_5g]	74
C8	8a	ZnCl_2_	[ZnCl_2_8a]	64

### Antimycobacterial activity

The antimicrobial effect of the 21 synthesized compounds was initially accessed against a panel of 4 microbial indicator strains (*Escherichia coli* ATCC35218, *Staphylococcus aureus* ATCC33592, *Septoria tritici* MUCL45408 and *Mycobacterium tuberculosis* H37Ra). Cytotoxicity was investigated using an epithelia cell line from human lung carcinoma (Calu-3). The results are summarized in Tables 3 and S1.†

**Table 3:** Minimum inhibitory concentration (MIC) of investigated imidazo[1,5-*a*]quinolines. Ec: *Escherichia coli* ATCC35218, Sa: *Staphylococcus aureus* ATCC33592; Str*: Septoria tritici* MUCL45408, Mtb: *Mycobacterium tuberculosis* H37Ra; MICs given in μM. Calu-3: epithelia cell line from lung carcinoma, “—” indicates no effect at 100μM and “+” toxic effects at 100 μM

Number	Ec	Sa	Str	Mtb	Calu-3
5a	>196	>196	>196	>196	—
5b	>200	>200	>200	>200	—
5c	>199	>199	>199	25	+
5d	>172	>172	>172	86–43	—
5e	>148	>148	>148	1	+
5f	>136	>136	>136	34	—
5g	>144	>144	>144	1–0.5	—
5h	>127	>127	>127	2	+
5i	>183	>183	>183	46	—
6a	>198	>198	>198	12–6	—
6b	>199	>199	>199	50–25	+
7a	>172	>172	>172	5–1	+
8a	>155	>155	>155	>155	—
C1	>140	>140	>140	2	—
C2	>132	>132	>132	2	—
C3	>127	>127	16–8	2	+
C4	> 126	31.5	63	2–1	+
C5	>140	17–9	17	2–1	+
C6	>120	>120	>120	8	—
C7	>126	126	126	2–1	—
C8	>117	>117	>117	>117	—

From the investigated compounds, 13 showed no cytotoxicity at a high dose of 100 μM. Simultaneously, 4 (5g, C1, C2, C7) of the non-toxic compounds exhibited an intriguing MIC of ≤2 μM against our surrogate Mtb strain H37Ra. These values are in range or only slightly higher than MICs of the reference drugs used in this study (MIC of gentamicin against H37Ra = 4 μM and MIC of rifampicin 0.07 μM; see Table S1 in ESI†) and literature reported values against various Mtb strains (*e.g.* isoniazid = 0.3–1.4 μM; levofloxacin = 0.8–1.4 μM, amikacin = 0.4–1.7 μM, bendaquiline = 0.03–1 μM, ethambutol = 0.3–3 μM or ethionamide = 10 μM).^23–26^

Interestingly, the low antimycobacterial activity of 5c against H37Ra (25 μM) could be potentiated by a factor of 12.5 by complexation with either Zn^2+^ (C1), Fe3^+^ (C2) or Cu2^+^ (C3). Although the copper-acetate complex maintained the initially observed cytotoxic properties of 5c, we observed no toxicity of the Zn^2+^ and Fe^3+^ complexes towards the Calu-3 lung carcinoma cell line at 100 μM.

The inhibitory effects of 5g, C1, C2, and C7 were specifically observed against Mtb, while the other test strains were not affected. These 4 compounds were then screened in a second-tier assay against wild-type *Mycobacterium tuberculosis* strain ATCC 35801. After activity confirmation at a high dose of 20 μM, the MIC values were determined. Cytotoxicity was also revaluated in the human liver cancer cell line HepG2 (Table 4, Fig. S1–S6†).

**Table 4:** IC_50/90_ values of prioritized compounds against BSL-3 *M. tuberculosis* ATCC 35801. Prioritization was based on primary antimicrobial screening results. Values are given in μM

Number	Mtb ATCC 35801			HepG2
	Inhibition at 20 μM	IC_50_	IC_90_	IC_50_
5g	82%	86.5	136.2	>100
C1	90%	6	7.7	>100
C2	26%	n.d.	n.d.	n.d.
C7	n.d.	9.1	17.7	83.3

Although the promising activity of C1 against H37Ra could be transferred to the wild-type strain ATCC 35801 (IC_50/90_ = 6/7.7 μM), C2 showed no growth inhibitory effects against this strain. Similarly, the initially promising growth inhibitory activity of 5g against the Mtb surrogate (1 μM) could not be transferred to the virulent Mtb strain (IC_50/90_ = 86.5/132.2 μM), but the potency was strongly increased (IC_50/90_ = 9.14/17.72 μM) upon Zn^2+^ complexation (C7). The other designed imidazo[1,5-*a*]quinoline-Zn^2+^ complexes were either cytotoxic (C4–C5) or inactive (C6, C8).

## Conclusions

In summary, we synthesized a series of 21 imidazo[1,5-*a*]quinolines and screened them against the surrogate strain *M.tuberculosis* HR37a, as well as against *Escherichia coli, Staphylococcus aureus,* the fungal plant pathogen *Septoria tritici* and lung carcinoma cell line Calu-3. Inspired by previous reports, we also decided to include metal-chelated variants in the compound series. To the best of our knowledge, the present study is the first to describe the antimycobacterial effects of imidazo[1,5-*a*]quinolines. Interestingly, the initially moderate antimycobacterial activity of 5c against H37Ra (25 μM) could be potentiated by a factor of 12.5 by complexation with either Zn^2+^ or Fe^2+^, while the cytotoxic effect was reduced (>100 μM).

Besides compounds C1 and C2, two additional compounds (5g and C7) exhibited specific HR37a activity (<2 μM) and were therefore followed up in a second-tier assay against BSL-3 *Mycobacterium tuberculosis* ATCC 35801. Ultimately, we identified two zinc complexes C1 and C7, with intriguing anti-tuberculosis activity and low cytotoxicity. Although these compounds surfaced from a relatively small derivative library, their *in vitro* potency was comparable to that of developed Mtb drugs. However, the major challenge regarding TB is the treatment of multi-drug resistant forms of the disease for which currently available drugs are not effective.^27^ Hence, it would be of interest to profile our candidates against RR-TB, MDR-TB and XDR-TB strains. Additional work investigating the mode of action, frequency of resistance, cross resistance, collateral susceptibility effects as well as ADME Tox properties of target compounds is crucial to evaluate their clinical potential.

## Author contributions

M. Marner performed the biological studies and prepared the draft. N. Kulhanek performed the synthesis and analytics and prepared the draft. N. Kulhanek and M. Marner contributed equally to this publication. Johanna Eichberg performed cytotoxicity analysis and Michael Dal Molin conducted BSL-3 work. M. Kirchner analysed and solved the XRD data.

## Conflicts of interest

There are no conflicts to declare.

## Supplementary Material

MD-015-D4MD00086B-s001

MD-015-D4MD00086B-s002
