# Supplementary material for: Design, synthesis and antimycobacterial activity of imidazo[1,5-a]quinolines and their zinc-complexes
Source: RSC Med Chem. 2024 Mar 12;15(5):1746–50. doi: 10.1039/d4md00086b (PMC11110795; doi:10.1039/d4md00086b)
Supplement: MD-015-D4MD00086B-s001 [file MD-015-D4MD00086B-s001.pdf]

Electronic Supplementary Material (ESI) for RSC Medicinal Chemistry.
This journal is © The Royal Society of Chemistry 2024

Supporting Information

Design, synthesis and antimycobacterial activity of Imidazo[1,5-a]quinolines and their Zinc-complexes

Michael Marner,[a‡] Niclas Kulhanek,[b‡] Johanna Eichberg,[a,h] Kornelia Hardes ,[a,h] Michael Dal Molin,[e,f] Jan Rybniker,[e,f,g] Michael Kirchner,[b] Till F. Schäberle,[a,c,d] and Richard Göttlich*[b]

a. Fraunhofer-Institute for Molecular Biology and Applied Ecology (IME) Branch Bioresources, Ohlebergsweg 12, 35392 Giessen, Germany

b. Institute of Organic Chemistry, Justus-Liebig-University, Heinrich-Buff-Ring 17, Giessen 35392, Germany

c.Institute for Insect Biotechnology, Justus-Liebig-University Giessen, Ohlebergsweg 12, 35392, Giessen, Germany

d. German Center for Infection Research (DZIF); Partner Site Giessen-Marburg-Langen, Ohlebergsweg 12, 35392 Giessen, Germany.

e. Department I of Internal Medicine, University of Cologne, 50937 Cologne, Germany

f. Center for Molecular Medicine Cologne (CMMC), University of Cologne, 50931 Cologne, Germany

g.German Center for Infection Research (DZIF), Partner Site Bonn-Cologne, Cologne Germany

f. BMBF Junior Research Group in Infection Research „ASCRIBE", Branch for Bioresources of the Fraunhofer Institute for Molecular Biology and Applied Ecology IME, Ohlebergsweg 12, 35392 Giessen, Germany

‡ *The authors contributed equally*

Table of Contents

1. General Considerations 2
2. Synthesis of 3-Bromo-imidazo[1,5-a]quinolines 2
   2.1 General procedure for amid formation 2
   2.2 General procedure for imidazo ring closure 3
   2.3 General procedure for bromination 5
3. Methods for the Negishi coupling 6
4. General procedure of complex formation 9
5. Copies of ^{1}H and ^{13}C NMR Spectra 13
5. XRD data 31
6. Biological Methods 33
   6.1 Antimicrobial Assays 33
   6.2 Cytotoxicity assays 34
7. Bioactivity data 36
   7.1 Antimicrobial data 36
   7.2 Cytotoxicity data 38
8. References 39
9. Author Contribution 40

1. General Considerations

All solvents were purified by distillation prior to use. Anhydrous solvents were used from ACROS Organics™ as AcroSeal™ bottles. Commercially available chemicals were used as obtained from the supplier unless otherwise stated. Syntheses prepared under anhydrous conditions were generally performed under standard Schlenk technique in nitrogen atmosphere. For purification by column chromatography, silica gel 60 (Merck) was used. ^{1}H and ^{13}C NMR spectra were recorded at the Bruker Avance II 400, the Bruker Avance III 400 and the Bruker Avance II 200 "Microbay" spectrometers in deuterated solvents. ^{1}H and ^{13}C chemical shifts were determined by reference to the residual solvent signals. High-resolution ESI mass spectra were recorded in methanol with an ESImicroTOF spectrometer from Bruker Daltonics in positive ion mode unless otherwise stated. As a power supply, the Sky Toppower PS1110 was used.

All not specifically stated reactions were performed according to our previous publication.[1]

2. Synthesis of 3-Bromo-imidazo[1,5-a]quinolines

2.1 General procedure for amid formation

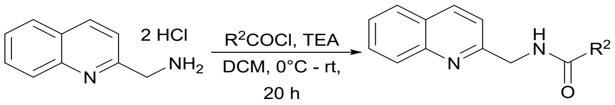

The procedure was based on the work of *Pelletier et al.*[2]:

60 mL of dry DCM was added to 2-aminomethylquinolin dihydrochloride (1 eq) under N_2-atmosphere. The suspension was cooled to 0°C and dry TEA (3.4 eq) was added. The yellow solution was kept at 0 °C for 30 min. Acid chloride R^2COCl (1.1 eq) was added and the solution stirred for 20 h at room temperature. Finally, 60 mL sat. Na_2CO_3 solution was added and the aqueous phase was extracted twice with 60 mL DCM. The combined organic phases were dried over Na_2SO_4, filtered and the solvent removed *in vacuo*. The product was not further purified and used directly in the next step.

***N*-(Quinolin-2-ylmethyl)-1-naphthamide (2b)**

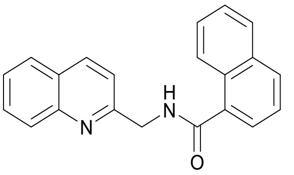

This compound was prepared according to the general procedure for amid formation from 4.811 g (20.82 mmol) 2-aminomethylquinolin dihydrochloride with 3.4 ml (22.90 mmol) 1-naphthoyl chloride. The red crude solid was used without further purification.

N-(Quinolin-2-ylmethyl)-diphenylurea (2c)

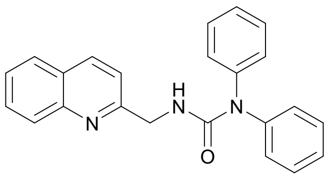

This compound was prepared according to the general procedure for amid formation from 5.685 g (24.60 mmol) 2-aminomethylquinolin dihydrochloride with 6.269 g (27.06 mmol) *N*-diphenylcarbamoyl chloride. The red crude solid was used without further purification.

N-(Quinolin-2-ylmethyl)pyridinylamide (2d)

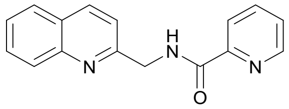

This compound was prepared according to the general procedure for amid formation from 4.724 g (20.44 mmol) 2-aminomethylquinolin dihydrochloride with 3.183 g (22.48 mmol) 2-picolinic acid chloride. The red crude solid was used without further purification.

2.2 General procedure for imidazo ring closure

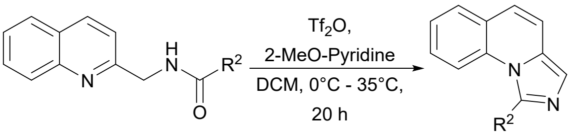

The procedure was based on the work of *Pelletier et al.*[2]:

The quinolin amide (1 eq) was placed in 60 mL of dry DCM under an N_2 atmosphere and cooled to 0°C. The solution was first treated with 2-methoxypyridine (1.1 eq) and then slowly with Tf_2O (1.2 eq). The reaction solution was stirred at 35°C for 20 h. 20 mL sat. Na_2CO_3 solution was added slowly and the aqueous phase was extracted twice with 60 mL DCM. The combined organic phases were dried over Na_2SO_4 and the solvent removed *in vacuo*. Purification was carried out by silica chromatography.

1-(Naphthalen-1-yl)imidazo[1,5-a]quinoline (3b)

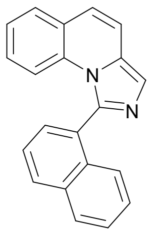

The compound was prepared according to the general procedure of imidazo ring closure from the entire unpurified compound **2b**. The crude mixture was purified by silica chromatography (1:4 ethylacetat:*n*-hexan). The pure product was obtained as a brown solid (4.673 g, 15.88 mmol, 76% over 2 steps). ^{1}H NMR (400 MHz, $CDCl_3$): δ = 8.07 (d, 1H), 7.97 (d, J = 1.10 Hz, 1H), 7.73 (dd, J = 7.02, 1.31 Hz, 1H), 7.69 (s, 1H), 7.67 – 7.58 (m, 2H), 7.50 (ddd, J = 8.15, 5.80, 2.21 Hz, 1H), 7.43 (d, J = 9.39 Hz, 1H), 7.38 – 7.31 (m, 2H), 7.22 (ddd, J = 7.90, 7.07, 1.23 Hz, 1H), 7.09 (d, J = 9.41 Hz, 1H), 7.01 – 6.96 (m, 1H), 6.91 (ddd, J = 8.58, 7.05, 1.55 Hz, 1H) ppm; ^{13}C NMR (101 MHz, $CDCl_3$): δ = 140.5, 133.9, 132.7, 132.4, 131.4, 130.4, 130.3, 129.0, 128.6, 128.6, 127.9, 127.3, 126.6, 125.7, 125.6, 125.6, 125.2, 122.3, 121.8, 117.3, 117.2 ppm; HRMS(ESI): m/z calculated for $C_{21}H_{15}N_2$ {M+H$^+$}: 295.1230. Found: 295.1230.

***N*,*N*-Diphenylimidazo[1,5-a]quinolin-1-amine (3c)**

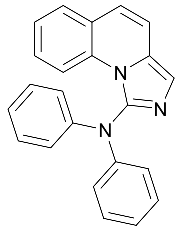

The compound was prepared according to the general procedure of imidazo ring closure from the entire unpurified compound **2c**. The crude mixture was purified by silica chromatography (1:2 ethylacetat:*n*-hexan). The pure product was obtained as an orange solid (7.044 g, 21.00 mmol, 85% over 2 steps). ^{1}H NMR (400 MHz, $CDCl_3$): δ = 8.63 – 8.55 (m, 1H), 7.61 – 7.52 (m, 1H), 7.44 (s, 1H), 7.34 – 7.17 (m, 7H), 7.07 – 6.95 (m, 7H) ppm; ^{13}C NMR (101 MHz, $CDCl_3$): δ = 145.5, 140.2, 132.0, 129.7, 128.9, 128.5, 128.4, 125.6, 125.5, 123.6, 121.8, 121.4, 120.5, 117.2, 116.9 ppm; HRMS(ESI): m/z calculated for $C_{23}H_{18}N_3$ {M+H$^+$}: 336.1495. Found: 336.1498.

1-(2-Pyridinyl)imidazo[1,5-a]quinoline (3d)

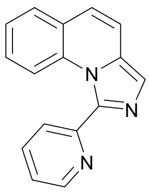

The compound was prepared according to the general procedure of imidazo ring closure from the entire unpurified compound **2d**. The crude mixture was purified by silica chromatography (4:6 ethylacetat:*n*-hexan). The pure product was obtained as a grey solid (1.349 g, 5.50 mmol, 27% over 2 steps). ^{1}H NMR (400 MHz, $CDCl_3$): δ = 8.80 (ddd, J = 4.81, 1.83, 0.98 Hz, 1H), 8.26 (dt, J = 7.82, 1.09 Hz, 1H), 8.11 (td, J = 7.82, 1.77 Hz, 1H), 7.90 (s, 1H), 7.78 (dd, J = 7.83, 1.57 Hz, 1H), 7.67 – 7.59 (m, 2H), 7.56 (ddd, J = 7.92, 7.31, 1.07 Hz, 1H), 7.50 (d, J = 9.47 Hz, 1H), 7.46 – 7.36 (m, 2H) ppm; ^{13}C NMR (101

MHz, $CDCl_3$): δ = 150.3, 145.3, 138.6, 137.5, 131.0, 130.7, 129.6, 129.0, 128.3, 127.1, 126.7, 126.6, 126.2, 119.2, 115.8, 114.7 ppm; HRMS(ESI): m/z calculated for $C_{16}H_{12}N_3$ {M+H$^+$}: 246.1026. Found: 246.1028.

2.3 General procedure for bromination

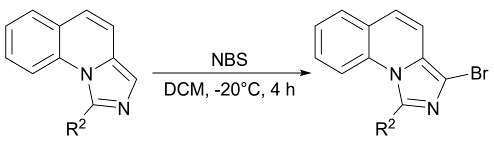

The imidazo[1,5-a]quinoline was dissolved in 60 mL dry DCM under an N_2 atmosphere and cooled to -20 °C. 1.1 eq NBS was slowly added to the solution. The reaction solution was stirred at -20°C for 4 h. 20 ml sat. $Na_2S_2O_3$ solution was applied and the aqueous phase extracted twice with 40 mL DCM. The combined organic phases were dried over Na_2SO_4, filtered, and the solvent removed *in vacuo*. Purification was carried out by silica chromatography.

3-Bromo-1-(naphthalen-1-yl)imidazo[1,5-a]quinoline (4b)

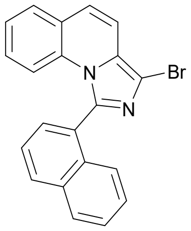

The compound was prepared according to the general procedure for bromination from 4.403 g (14.96 mmol) compound **3b**. The crude mixture was purified by silica chromatography (1:4 ethylacetat:*n*-hexan (v/v)). The pure product was obtained as a pale brown solid (5.280 g, 14.15 mmol, 95%). ^{1}H NMR (400 MHz, $CDCl_3$): δ = 8.08 (d, J = 0.94 Hz, 1H), 7.97 (d, J = 1.05 Hz, 1H), 7.72 (dd, J = 7.06, 1.32 Hz, 1H), 7.66 – 7.60 (m, 2H), 7.51 (ddd, J = 8.16, 5.99, 2.08 Hz, 1H), 7.42 – 7.32 (m, 3H), 7.26 – 7.21 (m, 1H), 7.15 (d, J = 9.44 Hz, 1H), 6.99 – 6.89 (m, 2H) ppm; ^{13}C NMR (101 MHz, $CDCl_3$): δ = 139.8, 133.8, 132.5, 132.2, 130.6, 130.5, 129.2, 128.9, 128.6, 128.2, 127.9, 127.5, 126.7, 125.7, 125.7, 125.6, 125.5, 122.6, 117.0, 116.2, 109.3 ppm; HRMS(ESI): m/z calculated for $C_{21}H_{14}BrN_2$ {M+H$^+$}: 373.0335. Found: 373.0332.

3-Bromo-*N*,*N*-diphenylimidazo[1,5-a]quinolin-1-amine (4c)

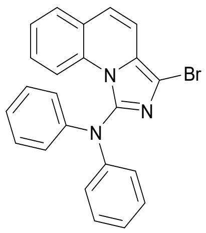

The compound was prepared according to the general procedure for bromination from 6.746 g (20.11 mmol) compound **3c**. The crude mixture was purified by silica chromatography (1:9 ethylacetat:*n*-hexan (v/v)). The pure product was obtained as a pale red solid (6.240 g, 15.06 mmol, 75%). ^{1}H NMR (400 MHz, $CDCl_3$): δ = 8.61 – 8.51 (m, 1H), 7.62 – 7.54 (m, 1H), 7.35 – 7.29 (m, 2H), 7.27 – 7.19 (m, 5H), 7.08 – 6.98 (m, 7H) ppm; ^{13}C NMR (101 MHz, $CDCl_3$): δ = 145.1, 139.0, 131.9, 129.8, 129.7, 128.9, 128.7, 126.8, 126.1, 125.6, 123.8, 122.6, 121.5, 121.4, 116.9, 116.0, 107.1 ppm; HRMS(ESI): m/z calculated for $C_{23}H_{17}BrN_3$ {M+H$^+$}: 414.0601. Found: 414.0604.

3-Bromo-1-(2-pyridinyl)imidazo[1,5-a]quinoline (4d)

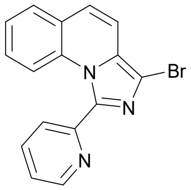

The compound was prepared according to the general procedure for bromination from 1.349 g (5.50 mmol) compound **3d**. The crude mixture was purified by silica chromatography (3:7 ethylacetat:*n*-hexan (v/v)). The pure product was obtained as a pale brown solid (0.703 g, 2.17 mmol, 40%). ^{1}H NMR (400 MHz, $CDCl_3$): δ = 8.81 (dt, J = 4.60, 1.29 Hz, 1H), 8.13 (td, J = 7.71, 1.68 Hz, 1H), 8.08 – 8.02 (m, 1H), 7.73 (dt, J = 7.82, 2.15 Hz, 1H), 7.62 (ddt, J = 8.41, 4.64, 2.29 Hz, 1H), 7.55 – 7.38 (m, 2H), 7.39 – 7.31 (m, 2H), 7.31 – 7.21 (m, 1H) ppm; ^{13}C NMR (101 MHz, $CDCl_3$): δ = 146.6, 140.9, 137.9, 131.7, 130.2, 129.6, 128.6, 126.9, 126.5, 125.9, 125.1, 124.8, 117.9, 115.6, 115.1, 110.6 ppm; HRMS(ESI): m/z calculated for $C_{16}H_{10}BrN_3Na$ {M+Na$^+$}: 345.9950. Found: 345.9950.

3. Methods for the Negishi coupling

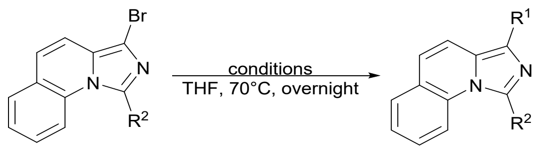

Method A: 0.400 g (1 eq) 3-Bromoimidazo[1,5-a]quinoline was dissolved in 10 mL dry THF under N_2 atmosphere and cooled to -78°C. 1.2 eq of *n*-BuLi (1.6 M) were slowly added dropwise and the solution was kept at -78° C for 30 min. 2.5 eq $ZnCl_2$ in dry THF (1 M) were added at -78°C and then stirred at room temperature for 1 h. 3 mol% of [$Pd_2(dba)_3$]$CHCl_3$, 6 mol% of P(*t*-Bu)$_3$ and 2 eq of the chosen coupling bromide were added and the reaction solution was stirred at 70° C for 20 h. The precipitate was filtered and washed first with THF, then with H_2O and again with THF. The solid was treated with 15 mL conc. $NH_{3\,(aq)}$ solution and the aqueous phase extracted three times with 20 mL DCM. The pure product was obtained after concentration *in vacuo* of the combined organic phases.

Method B: 0.400 g (1 eq) 3-Bromoimidazo[1,5-a]quinoline was dissolved in 10 mL dry THF under N_2 atmosphere and cooled to -78°C. 2 eq of *t*-BuLi (1.9 M) were slowly added dropwise and the solution was kept at -78° C for 30 min. 2.5 eq $ZnCl_2$ in dry THF (1 M) were added at -78°C and then stirred at room temperature for 1 h. 3 mol% of $[Pd_2(dba)_3]CHCl_3$, 6 mol% of $P(t\text{-}Bu)_3$ and 2 eq of the chosen coupling bromide were added and the reaction solution was stirred at 70° C for 20 h. 15 mL conc. $NH_{3\,(aq)}$ solution was added and the aqueous phase extracted three times with 20 mL DCM. The combined organic phases were concentrated *in vacuo* and the impure product was purified by silica chromatography.

3-(2-Pyridinyl)-1-(naphthalen-1-yl)-imidazo[1,5-a]quinoline (7a)

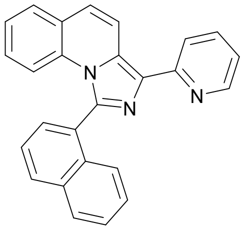

The compound was prepared according to **method A** for Negishi-coupling with 0.400 g (1.07 mmol) 3-bromo-1-(naphthalen-1-yl)imidazo[1,5-a]quinoline (**4b**) and 2-Bromopyridine as coupling partner. The product was obtained as a yellow solid (0.270 g, 0.73 mmol, 68%). ^{1}H NMR (400 MHz, $CDCl_3$): δ = 8.77 (d, J = 9.53 Hz, 1H), 8.70 (ddd, J = 4.90, 1.92, 0.92 Hz, 1H), 8.28 (dt, J = 8.09, 1.09 Hz, 1H), 8.10 (dt, J = 8.31, 1.10 Hz, 1H), 7.79 (dd, J = 6.98, 1.27 Hz, 1H), 7.72 (td, J = 7.72, 1.83 Hz, 1H), 7.70 – 7.63 (m, 2H), 7.51 (ddd, J = 8.16, 6.68, 1.31 Hz, 1H), 7.43 (dd, J = 8.48, 1.13 Hz, 1H), 7.34 (ddd, J = 8.29, 6.70, 1.29 Hz, 1H), 7.29 – 7.20 (m, 3H), 7.16 (ddd, J = 7.46, 4.88, 1.21 Hz, 1H), 7.00 – 6.89 (m, 2H) ppm; ^{13}C NMR (101 MHz, $CDCl_3$): δ = 154.8, 149.1, 140.2, 136.6, 134.0, 132.8, 132.4, 132.0, 131.6, 130.4, 129.0, 129.0, 128.6, 128.6, 128.0, 127.4, 126.7, 125.9, 125.9, 125.7, 125.3, 123.5, 121.1, 121.0, 119.9, 117.1 ppm; HRMS(ESI): m/z calculated for $C_{26}H_{18}N_3$ {M+H$^+$}: 372.1495. Found: 372.1498.

***N*,*N*-diphenyl-3-(pyridin-2-yl)imidazo[1,5-a]quinolin-1-amine (8a)**

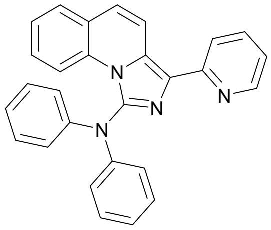

The compound was prepared according to **method A** for Negishi coupling with 0.400 g (0.97 mmol) 3-bromo-*N*,*N*-diphenylimidazo[1,5-a]quinolin-1-amine (**4c**) and 2-Bromopyridine as coupling partner. The product was obtained as a yellow solid (0.265 g, 0.64 mmol, 66%). ^{1}H NMR (400 MHz, $CDCl_3$): δ =

9.22 – 9.14 (m, 1H), 9.05 (d, *J* = 9.55 Hz, 1H), 8.91 – 8.84 (m, 1H), 8.61 (d, *J* = 8.26 Hz, 1H), 8.41 – 8.32 (m, 1H), 7.90 (dt, *J* = 7.97, 2.94 Hz, 1H), 7.82 (d, *J* = 9.53 Hz, 1H), 7.74 (t, *J* = 6.59 Hz, 1H), 7.65 – 7.57 (m, 2H), 7.50 – 7.40 (m, 4H), 7.29 – 7.20 (m, 6H) ppm; ^{13}C NMR (101 MHz, $CDCl_3$): δ = 147.0, 144.7, 144.2, 142.7, 141.4, 131.5, 130.8, 129.9, 129.0, 128.7, 126.8, 126.0, 125.6, 124.2, 122.1, 121.6, 121.5, 121.2, 117.0, 116.9 ppm; HRMS(ESI): m/z calculated for $C_{28}H_{21}N_4$ {M+H$^+$}: 413.1761. Found: 413.1752.

3-(2-Pyridinyl)-1-(2-pyridinyl)-imidazo[1,5-a]quinoline (6a)

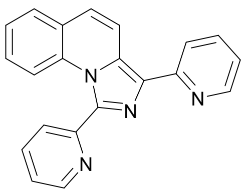

The compound was prepared according to **method B** for Negishi coupling with 0.200 g (0.62 mmol) 3-bromo-1-(2-pyridinyl)imidazo[1,5-a]quinoline (**4d**) and 2-Bromopyridine as coupling partner. The product was obtained as a yellow solid (0.052 g, 0.16 mmol, 26%). ^{1}H NMR (400 MHz, $CDCl_3$): δ = 8.93 – 8.87 (m, 1H), 8.77 (dt, J = 4.91, 1.42 Hz, 1H), 8.65 (d, J = 9.56 Hz, 1H), 8.44 (d, J = 8.23 Hz, 1H), 8.12 – 8.04 (m, 2H), 8.00 (td, J = 7.69, 1.81 Hz, 1H), 7.77 – 7.72 (m, 1H), 7.57 – 7.40 (m, 5H), 7.32 (ddd, J = 8.68, 7.20, 1.59 Hz, 1H) ppm; ^{13}C NMR (101 MHz, $CDCl_3$): δ = 149.7, 137.8, 131.9, 131.6, 129.1, 128.5, 127.3, 126.5, 126.1, 125.9, 124.8, 123.6, 122.0, 118.6, 117.5 ppm; HRMS(ESI): m/z calculated for $C_{21}H_{15}N_4$ {M+H$^+$}: 323.1291. Found: 323.1292.

3-Phenyl-1-(2-pyridinyl)-imidazo[1,5-a]quinoline (6b)

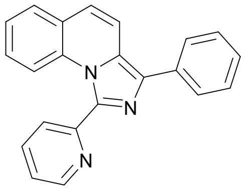

The compound was prepared according to **method B** for Negishi coupling with 0.100 g (0.31 mmol) 3-bromo-1-(2-pyridinyl)imidazo[1,5-a]quinoline (**4d**) and Bromobenzol as coupling partner. The product was obtained as a yellow solid (0.038 g, 0.12 mmol, 39%). ^{1}H NMR (400 MHz, $CDCl_3$): δ = 8.82 (ddd, J = 4.84, 1.75, 0.87 Hz, 1H), 8.49 (dt, J = 7.82, 1.05 Hz, 1H), 8.18 (td, J = 7.82, 1.79 Hz, 1H), 8.11 (dt, J = 6.79, 1.22 Hz, 1H), 8.07 – 8.03 (m, 2H), 7.80 (dd, J = 7.83, 1.41 Hz, 1H), 7.73 (d, J = 9.65 Hz, 1H), 7.66 (ddd, J = 7.82, 4.83, 1.08 Hz, 1H), 7.63 – 7.56 (m, 3H), 7.53 – 7.45 (m, 2H), 7.44 – 7.38 (m, 1H) ppm; ^{13}C NMR (101 MHz, $CDCl_3$): δ = 150.0, 143.6, 138.8, 136.7, 130.9, 130.5, 130.2, 129.6, 129.6, 129.2, 129.2, 128.6, 128.5, 127.8, 127.1, 126.6, 126.5, 126.3, 119.2, 115.8 ppm; HRMS(ESI): m/z calculated for $C_{22}H_{16}N_3$ {M+H$^+$}: 322.1339. Found: 322.1339.

4. General procedure of complex formation

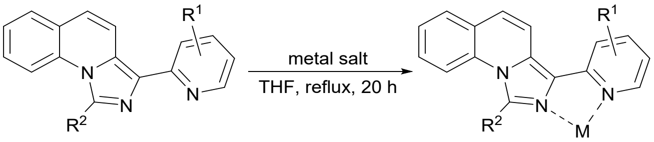

The ligand is dissolved in THF and 1.1 eq of metal salt is added. The solution is heated to reflux and stirred overnight. The precipitated powder is filtered off and washed with fresh THF. The solid is dried under high vacuum.

[(3-(2-Pyridinyl)-1-phenyl-imidazo[1,5-a]quinoline)-dichloro-zinc(II)] (C1)

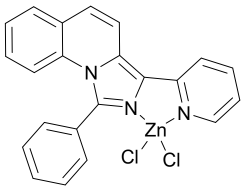

The compound was prepared according to the general procedure for complex formation with 0.400 g (1.24 mmol) 3-(2-pyridinyl)-1-phenyl-imidazo[1,5-a]quinoline (**5c**) and zinc (II) chloride as metal salt. The product was obtained as a grey solid (0.538 g, 1.18 mmol, 95%). ^{1}H and ^{13}C NMR in DMSO-d6 were inconclusive, but are displayed at the NMR section for comparison with the pure ligand. HRMS(ESI) (only ligand was found): m/z calculated for $C_{22}H_{16}N_3$ {M+H+}: 322.1339. Found: 322.1342. Elemental analysis calculated $C_{22}H_{15}Cl_2N_3Zn$: C: 57.74%, H: 3.30%, N: 9.18%; found: C: 57.31%, H: 3.18%, N: 8.83%. Crystal for XRD was grown by diffusion. The compound was dissolved in DMSO and placed in a chamber filled with Et_2O.

[(3-(2-Pyridinyl)-1-phenyl-imidazo[1,5-a]quinoline)-trichloro-iron(III)] (C2)

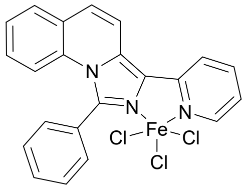

The compound was prepared according to the general procedure for complex formation with 0.100 g (0.31 mmol) 3-(2-pyridinyl)-1-phenyl-imidazo[1,5-a]quinoline (**5c**) and iron (III) chloride as metal salt. The product was obtained as a grey solid (0.148 g, 0.30 mmol, 97%). ^{1}H and ^{13}C NMR in DMSO-d6 were inconclusive, but are displayed at the NMR section for comparison with the pure ligand. HRMS(ESI) (only ligand was found): m/z calculated for $C_{22}H_{16}N_3$ {M+H+}: 322.1339. Found: 322.1334. Elemental analysis calculated $C_{22}H_{15}Cl_3N_3Fe$: C: 54.64%, H: 3.13%, N: 8.69%; found: C: 54.54%, H: 2.96%, N: 8.61%.

Crystal for XRD was grown by diffusion. The compound was dissolved in methanol and placed in a chamber filled with *n*-pentan.

[(3-(2-Pyridinyl)-1-phenyl-imidazo[1,5-a]quinoline)-diacetate-copper(II)] (C3)

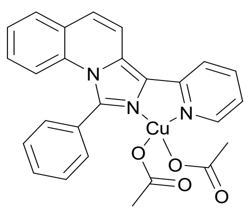

The compound was prepared according to the general procedure for complex formation with 0.200 g (0.62 mmol) 3-(2-Pyridinyl)-1-phenyl-imidazo[1,5-a]quinoline (**5c**) and copper (II) acetate as metal salt. The product was obtained as a green solid (0.310 g, 0.61 mmol, 98%). ^{1}H and ^{13}C NMR in DMSO-d6 were inconclusive, but are displayed at the NMR section for comparison with the pure ligand. HRMS(ESI) (only ligand was found): m/z calculated for $C_{22}H_{16}N_3$ {M+H$^+$}: 322.1339. Found: 322.1338. Elemental analysis calculated $C_{26}H_{21}CuN_3O_4$: C: 62.08%, H: 4.21%, N: 8.35%; found: C: 62.45%, H: 4.12%, N: 8.71%. Crystal for XRD was grown by diffusion. The compound was dissolved in chloroform and placed in a chamber filled with n-pentan.

[(3-(2-Pyridinyl)-1-(naphthalen-1-yl)-imidazo[1,5-a]quinoline)-dichloro-zinc(II)] (C4)

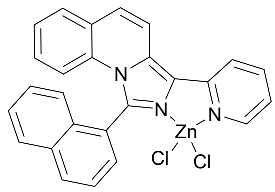

The compound was prepared according to the general procedure for complex formation with 0.397 g (1.07 mmol) 3-(2-pyridinyl)-1-(naphthalen-1-yl)-imidazo[1,5-a]quinoline (**7a**) and zinc (II) chloride as metal salt. The product was obtained as a grey solid (0.236 g, 0.47 mmol, 44%). ^{1}H and ^{13}C NMR in DMSO-d6 were inconclusive, but are displayed at the NMR section for comparison with the pure ligand. HRMS(ESI) (only ligand was detected): m/z calculated for $C_{26}H_{18}N_3$ {M+H$^+$}: 372.1495. Found: 372.1490. Elemental analysis calculated $C_{26}H_{17}Cl_2N_3Zn$: C: 61.51%, H: 3.38%, N: 8.28%; found: C: 61.19%, H: 3.43%, N: 7.91%.

[(3-(2-Pyrimidinyl)-1-phenyl-imidazo[1,5-a]quinoline)-dichloro-zinc(II)] (C5)

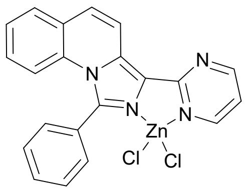

The compound was prepared according to the general procedure for complex formation with 0.400 g (1.24 mmol) 3-(2-pyrimidinyl)-1-phenyl-imidazo[1,5-a]quinoline (**5e**) and zinc (II) chloride as metal salt. The product was obtained as a grey solid (0.226 g, 0.49 mmol, 40%). ^{1}H and ^{13}C NMR in DMSO-d6 were inconclusive, but are displayed at the NMR section for comparison with the pure ligand. HRMS(ESI) (only ligand was found): m/z calculated for $C_{21}H_{15}N_4$ {M+H$^+$}: 323.1291. Found: 323.1290. Elemental analysis calculated $C_{21}H_{15}Cl_2N_4Zn$: C: 54.99%, H: 3.08%, N: 12.22%; found: C: 55.06%, H: 2.88%, N: 11.90%.

[(1-Phenyl-3-(5-phenylpyridin-2-yl)imidazo[1,5-a]quinoline)-dichloro-zinc(II)] (C6)

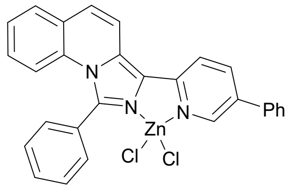

The compound was prepared according to the general procedure for complex formation with 0.493 g (1.24 mmol) 1-phenyl-3-(5-phenylpyridin-2-yl)imidazo[1,5-a]quinoline (**5f**) and zinc (II) chloride as metal salt. The product was obtained as a grey solid (0.422 g, 0.79 mmol, 64%). ^{1}H and ^{13}C NMR in DMSO-d6 were inconclusive, but are displayed at the NMR section for comparison with the pure ligand. HRMS(ESI) (only ligand was detected): m/z calculated for $C_{28}H_{20}N_3$ {M+H$^+$}: 398.1652. Found: 398.1650. Elemental analysis calculated $C_{28}H_{19}Cl_2N_3Zn$: C: 63.01%, H: 3.59%, N: 7.87%; found: C: 62.84%, H: 3.71%, N: 7.70%.

[(3-(3-Isoquinolinyl)-1- phenyl -imidazo[1,5-a]quinoline)-dichloro-zinc(II)] (C7)

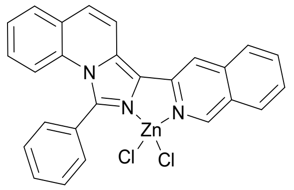

The compound was prepared according to the general procedure for complex formation with 0.461 g (1.24 mmol) 3-(3-isoquinolinyl)-1-phenyl-imidazo[1,5-a]quinoline (**5g**) and zinc (II) chloride as metal salt. The product was obtained as a grey solid (0.468 g, 0.92 mmol, 74%). ^{1}H and ^{13}C NMR in DMSO-d6 were inconclusive, but are displayed at the NMR section for comparison with the pure ligand. HRMS(ESI) (only ligand was detected): m/z calculated for $C_{26}H_{18}N_3$ {M+H$^+$}: 372.1495. Found: 372.1499. Elemental analysis calculated $C_{26}H_{17}Cl_2N_3Zn$: C: 61.51%, H: 3.38%, N: 8.28%; found: C: 61.46%, H: 3.11%, N: 8.06%.

[(*N*,*N*-Diphenyl-3-(pyridin-2-yl)imidazo[1,5-a]quinolin-1-amine)-dichloro-zinc(II)] (C8)

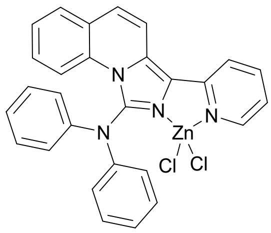

The compound was prepared according to the general procedure for complex formation with 0.400 g (0.97 mmol) *N*,*N*-diphenyl-3-(pyridin-2-yl)imidazo[1,5-a]quinolin-1-amine (**8a**) and zinc (II) chloride as metal salt. The product was obtained as a yellow solid (0.339 g, 0.62 mmol, 64%). ^{1}H and ^{13}C NMR in DMSO-d6 were inconclusive, but are displayed at the NMR section for comparison with the pure ligand. HRMS(ESI) (only ligand was detected): m/z calculated for $C_{28}H_{21}N_4$ {M+H$^+$}: 413.1761. Found: 413.1756. Elemental analysis calculated $C_{28}H_{20}Cl_2N_4Zn$: C: 61.28%, H: 3.67%, N: 10.21%; found: C: 61.17%, H: 3.43%, N: 9.89%.

5. Copies of ^{1}H and ^{13}C NMR Spectra

Compound 3b

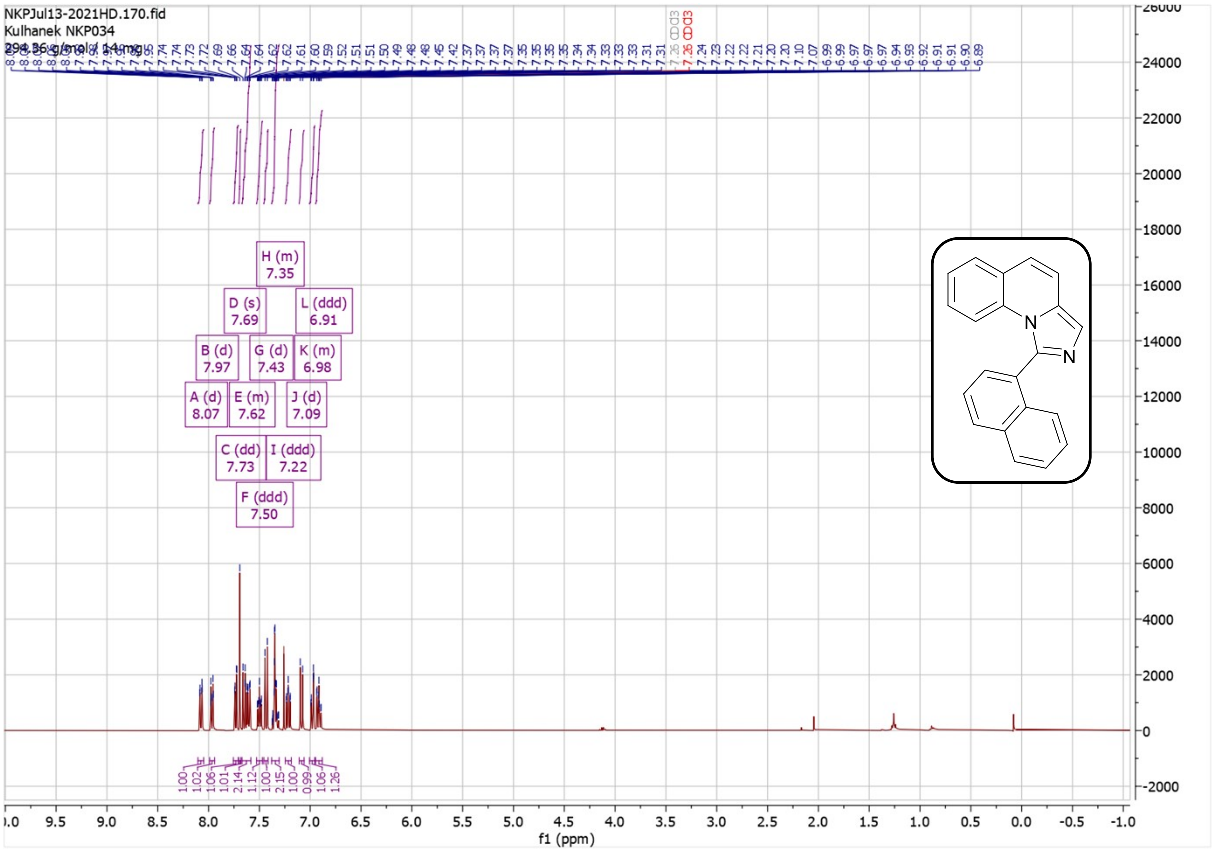

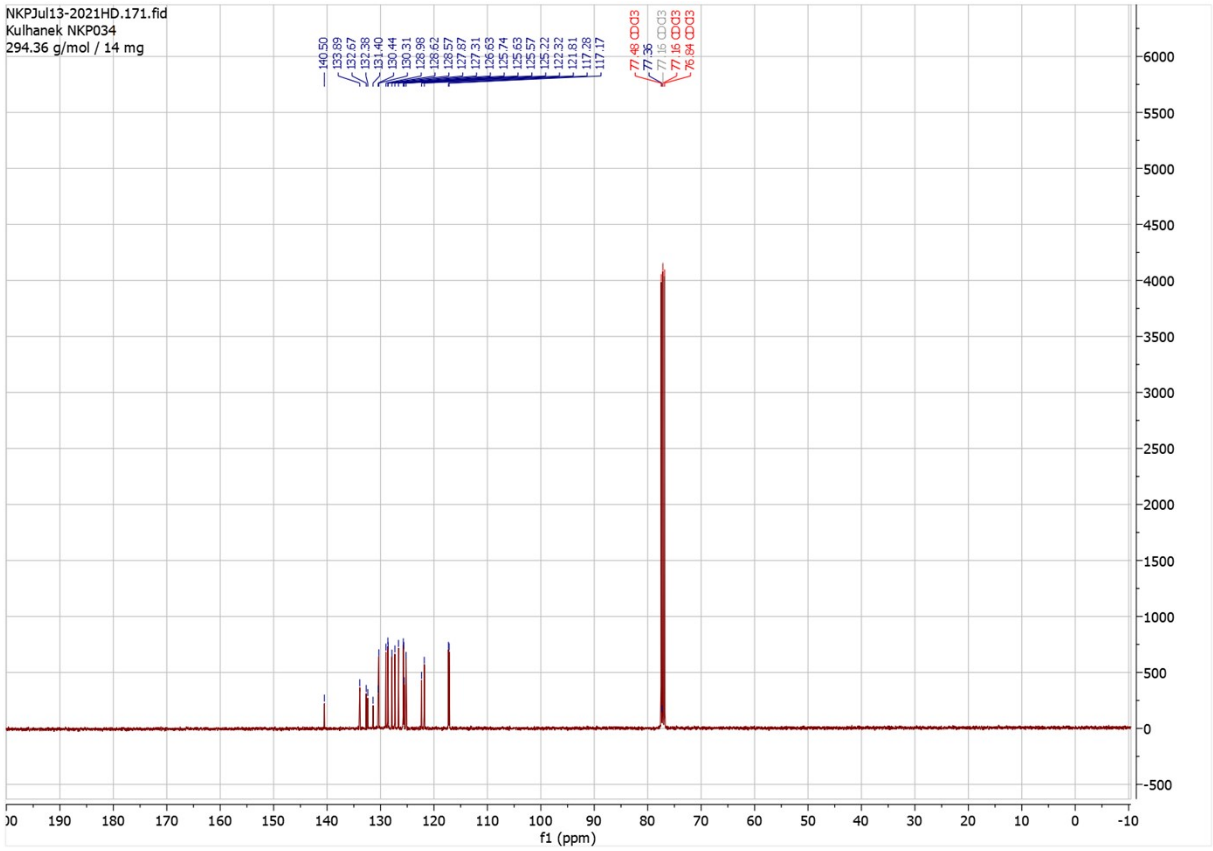

Compound 3c

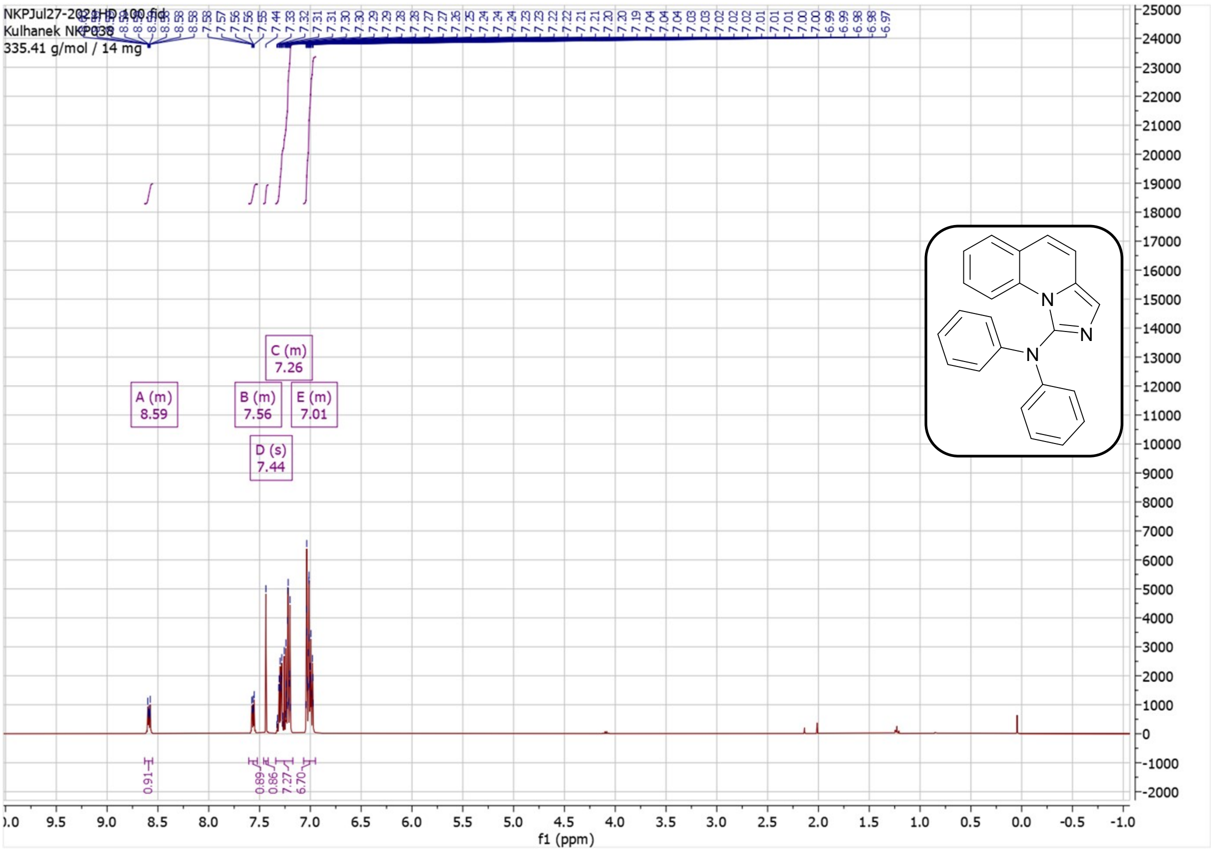

NKPJul27-2021HD.100.fid
Kulhanek NKP038
335.41 g/mol / 14 mg
A (m)
8.59
B (m)
7.56
C (m)
7.26
D (s)
7.44
E (m)
7.01
0.91
0.89
0.86
7.27
6.70
N
N
N
f1 (ppm)

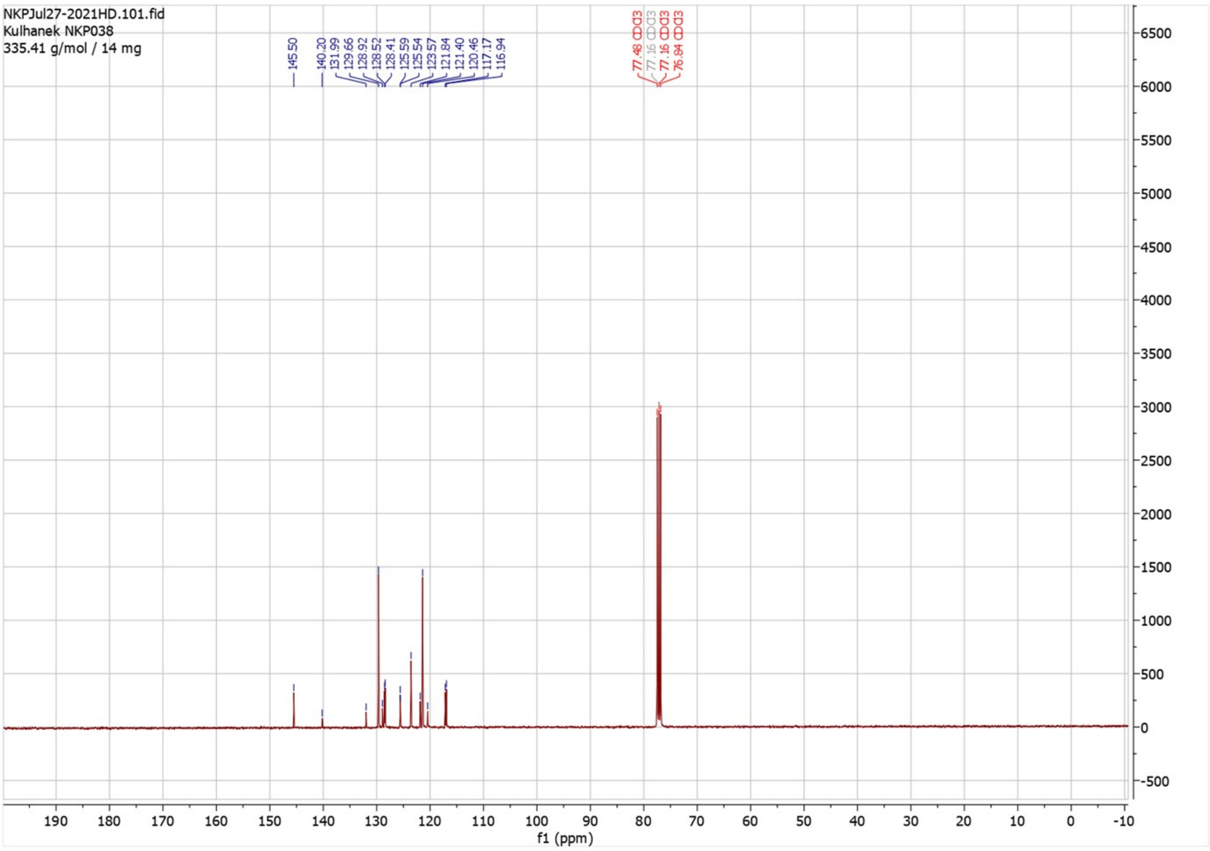

NKPJul27-2021HD.101.fid
Kulhanek NKP038
335.41 g/mol / 14 mg
145.50
140.20
131.99
129.66
128.92
128.52
128.41
125.59
125.54
123.57
121.84
121.40
120.46
117.17
116.94
77.48 CDCl3
77.16 CDCl3
77.16 CDCl3
76.84 CDCl3
f1 (ppm)

Compound 3d

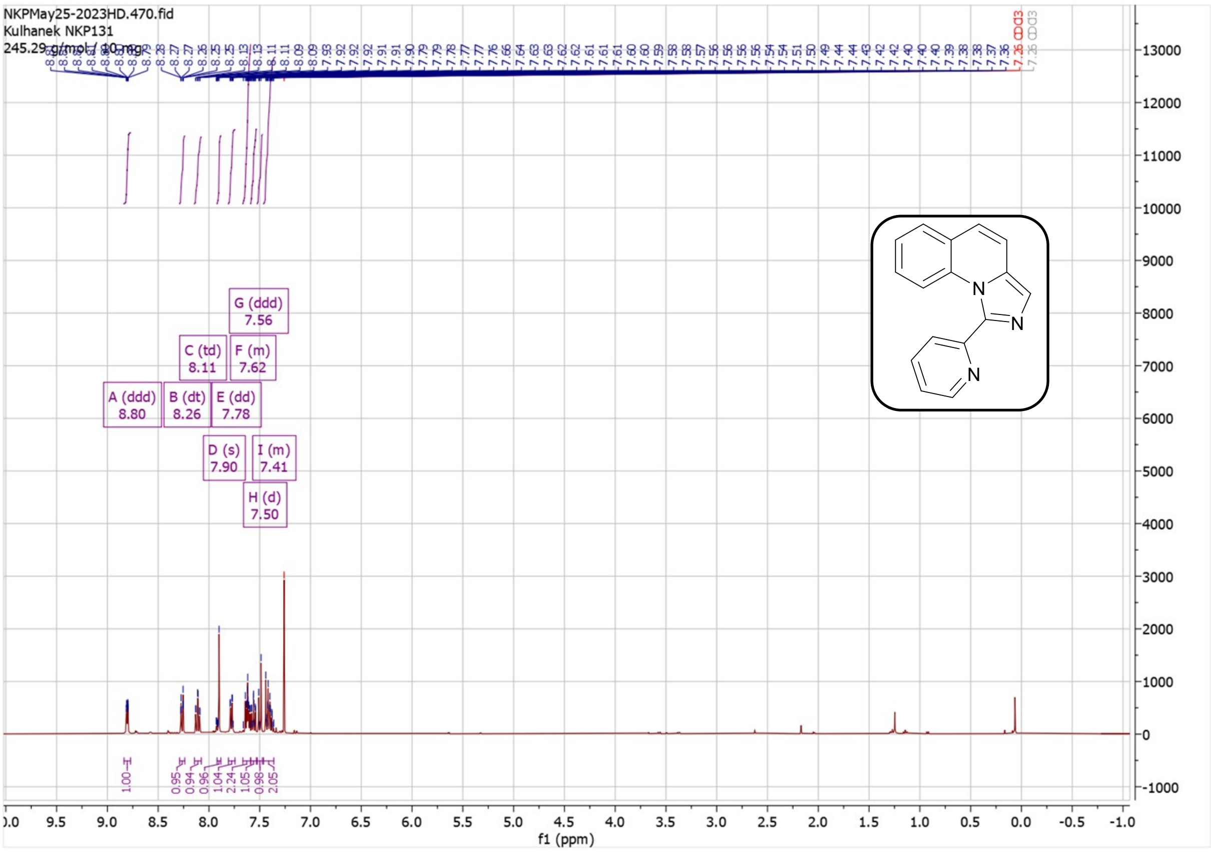
NKPMay25-2023HD.470.fid
Kulhanek NKP131
245.29 g/mol / 10 mg
A (ddd) 8.80
B (dt) 8.26
C (td) 8.11
D (s) 7.90
E (dd) 7.78
F (m) 7.62
G (ddd) 7.56
H (d) 7.50
I (m) 7.41
1.00
0.95
0.94
0.96
1.04
2.24
1.05
0.98
2.05
N
N
N
f1 (ppm)

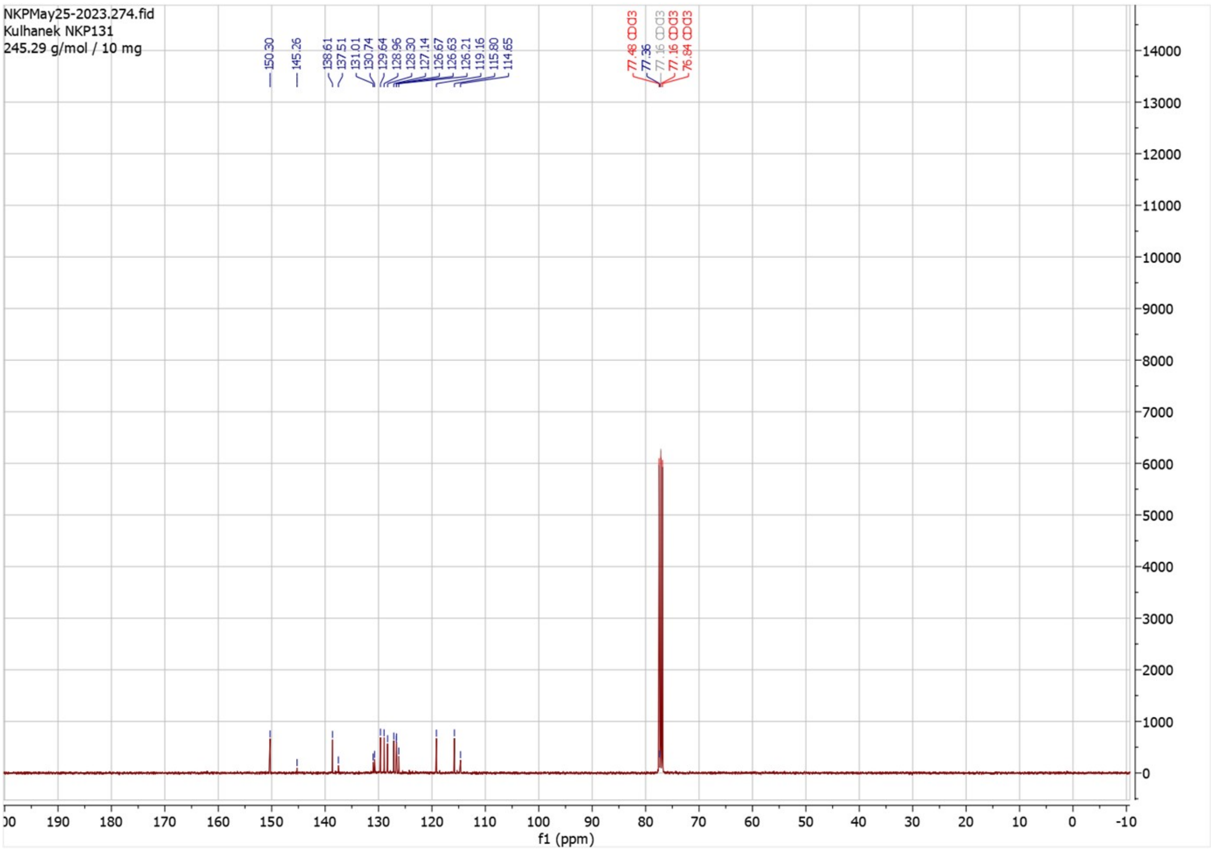
NKPMay25-2023.274.fid
Kulhanek NKP131
245.29 g/mol / 10 mg
150.30
145.26
138.61
137.51
131.01
130.74
129.64
128.96
128.30
127.14
126.67
126.63
126.21
119.16
115.80
114.65
77.48 CDCl3
77.36
77.16 CDCl3
77.16 CDCl3
76.84 CDCl3
f1 (ppm)

Compound 4b

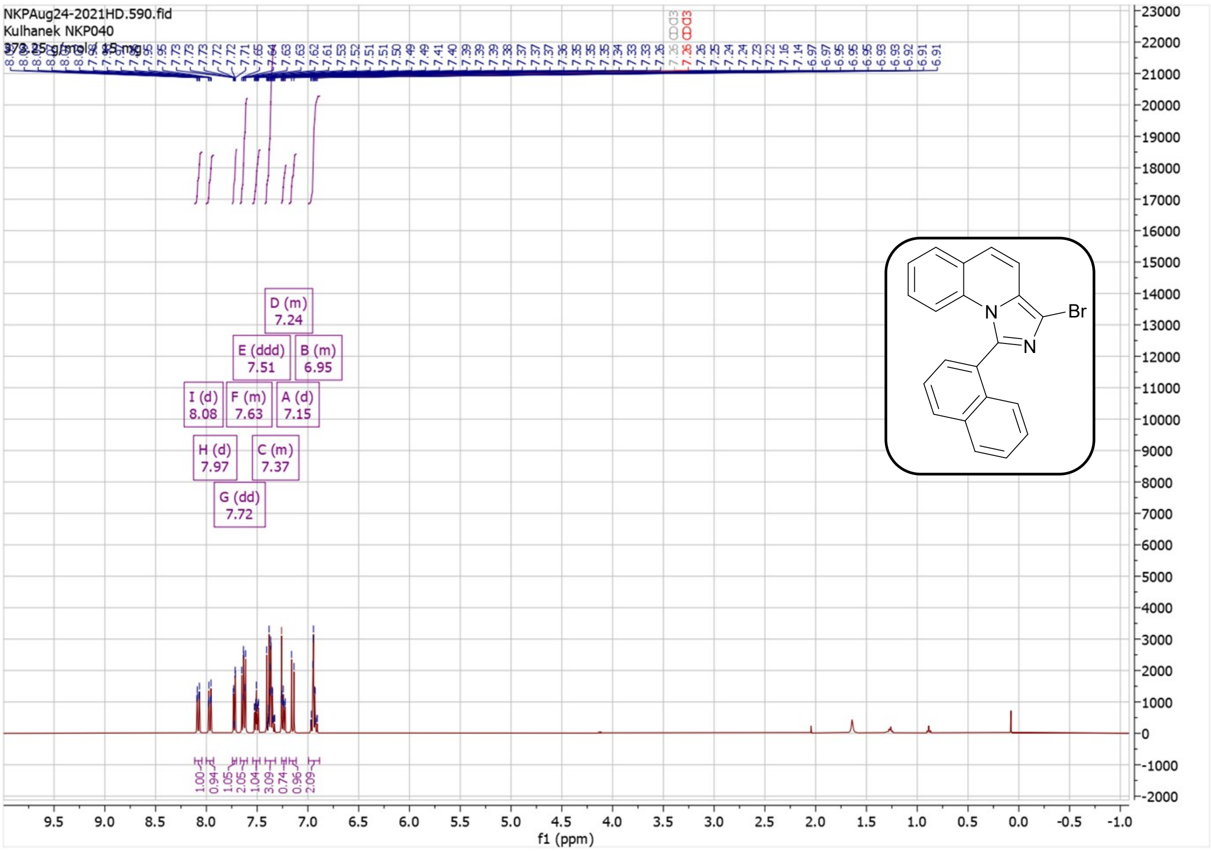
NKPAug24-2021HD.590.fid
Kulhanek NKP040
D (m) 7.24
E (ddd) 7.51
B (m) 6.95
I (d) 8.08
F (m) 7.63
A (d) 7.15
H (d) 7.97
C (m) 7.37
G (dd) 7.72
1.00 0.94 1.05 2.05 1.04 3.09 0.74 0.96 2.09
Br
N
N
f1 (ppm)

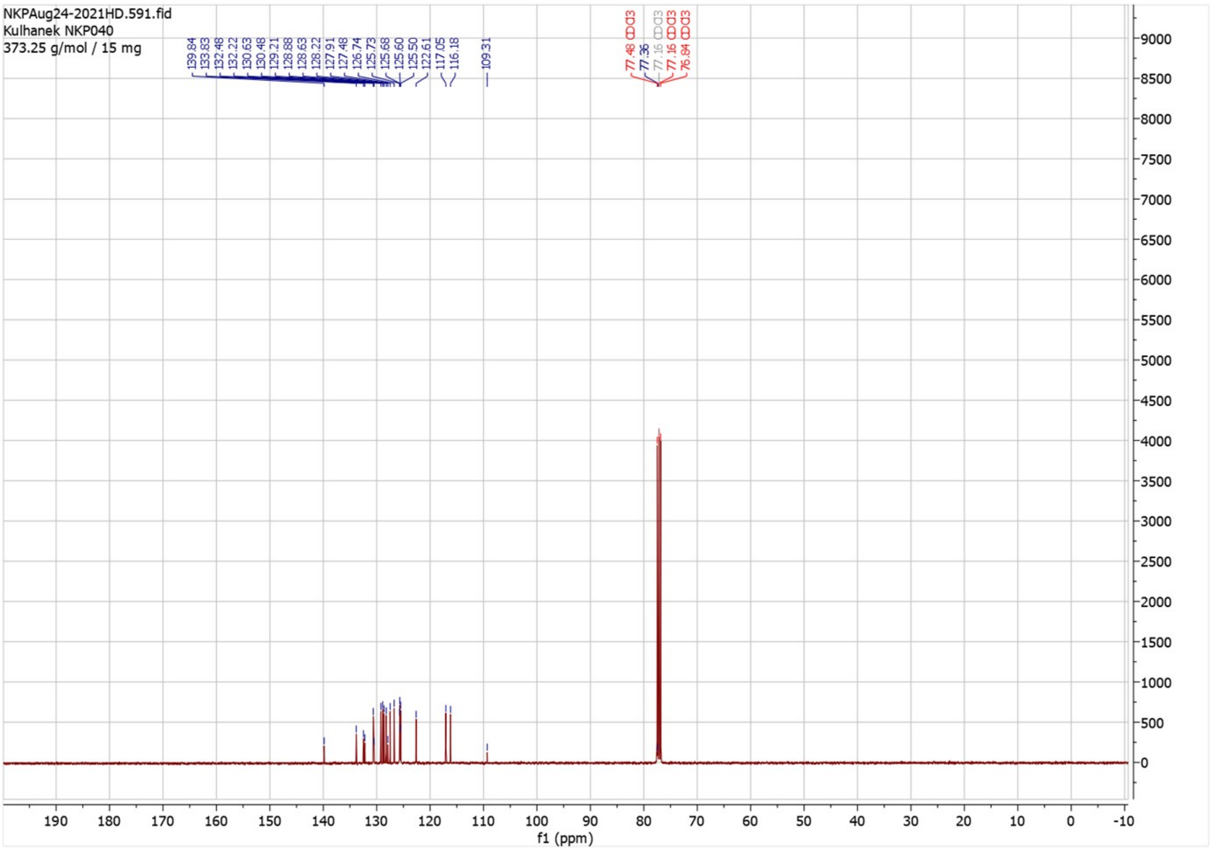
NKPAug24-2021HD.591.fid
Kulhanek NKP040
373.25 g/mol / 15 mg
139.84
133.83
132.48
132.22
130.63
130.48
129.21
128.88
128.63
128.22
127.91
127.48
126.74
125.73
125.68
125.60
125.50
122.61
117.05
116.18
109.31
77.48 CDCl3
77.36
77.16 CDCl3
77.16 CDCl3
76.84 CDCl3
f1 (ppm)

Compound 4c

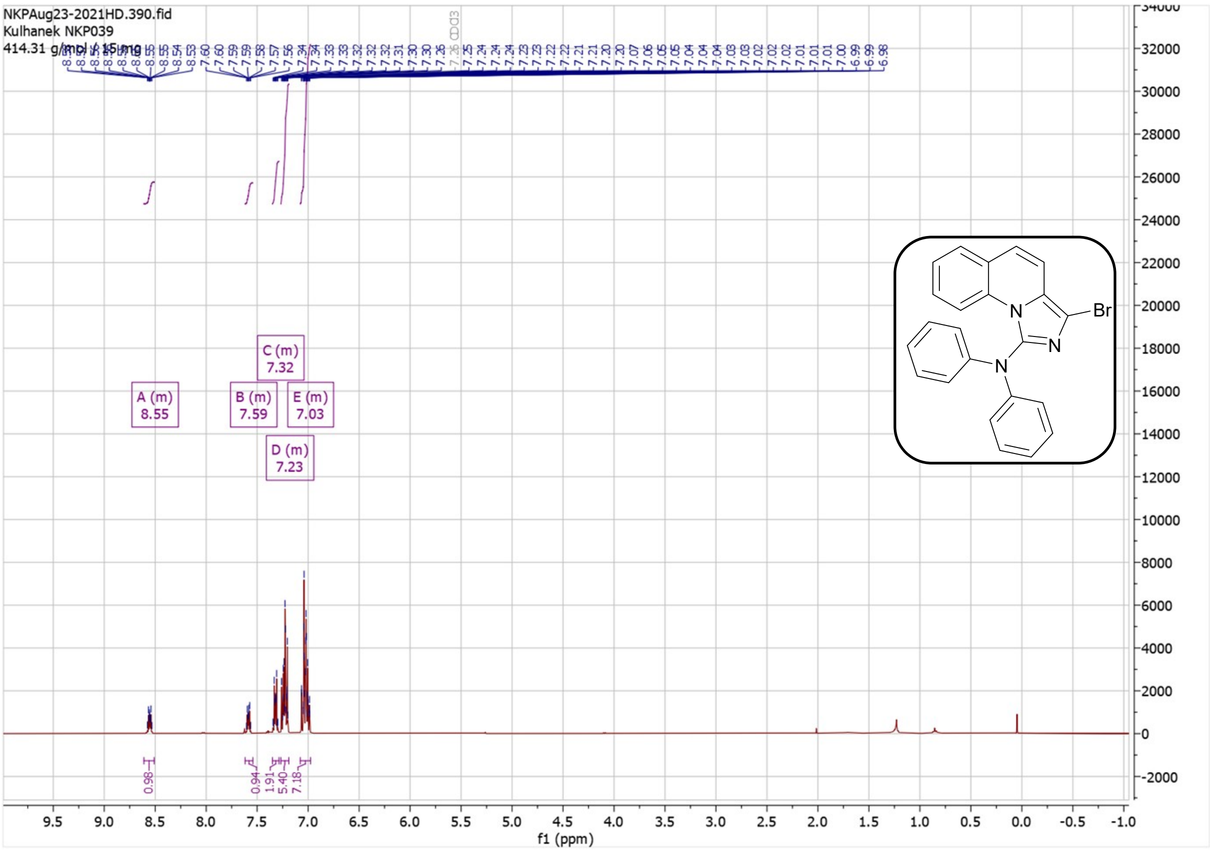

NKPAug23-2021HD.390.fid
Kulhanek NKP039
414.31 g/mol / 15 mg
A (m) 8.55
B (m) 7.59
C (m) 7.32
D (m) 7.23
E (m) 7.03
0.98
0.94
1.91
5.40
7.18
f1 (ppm)

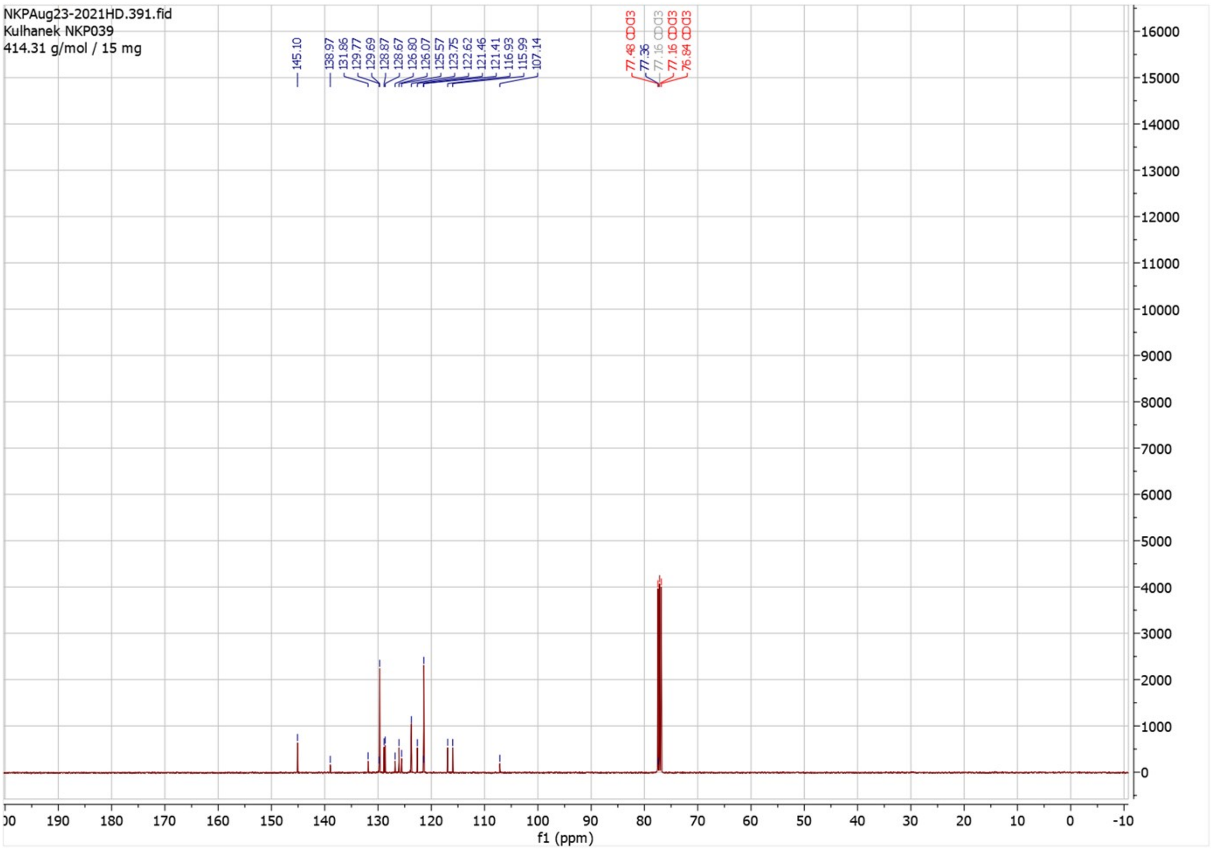

NKPAug23-2021HD.391.fid
Kulhanek NKP039
414.31 g/mol / 15 mg
145.10
138.97
131.86
129.77
129.69
128.87
128.67
126.80
126.07
125.57
123.75
122.62
121.46
121.41
116.93
115.99
107.14
77.48 CDCl3
77.36
77.16 CDCl3
77.16 CDCl3
76.84 CDCl3
f1 (ppm)

Compound 4d

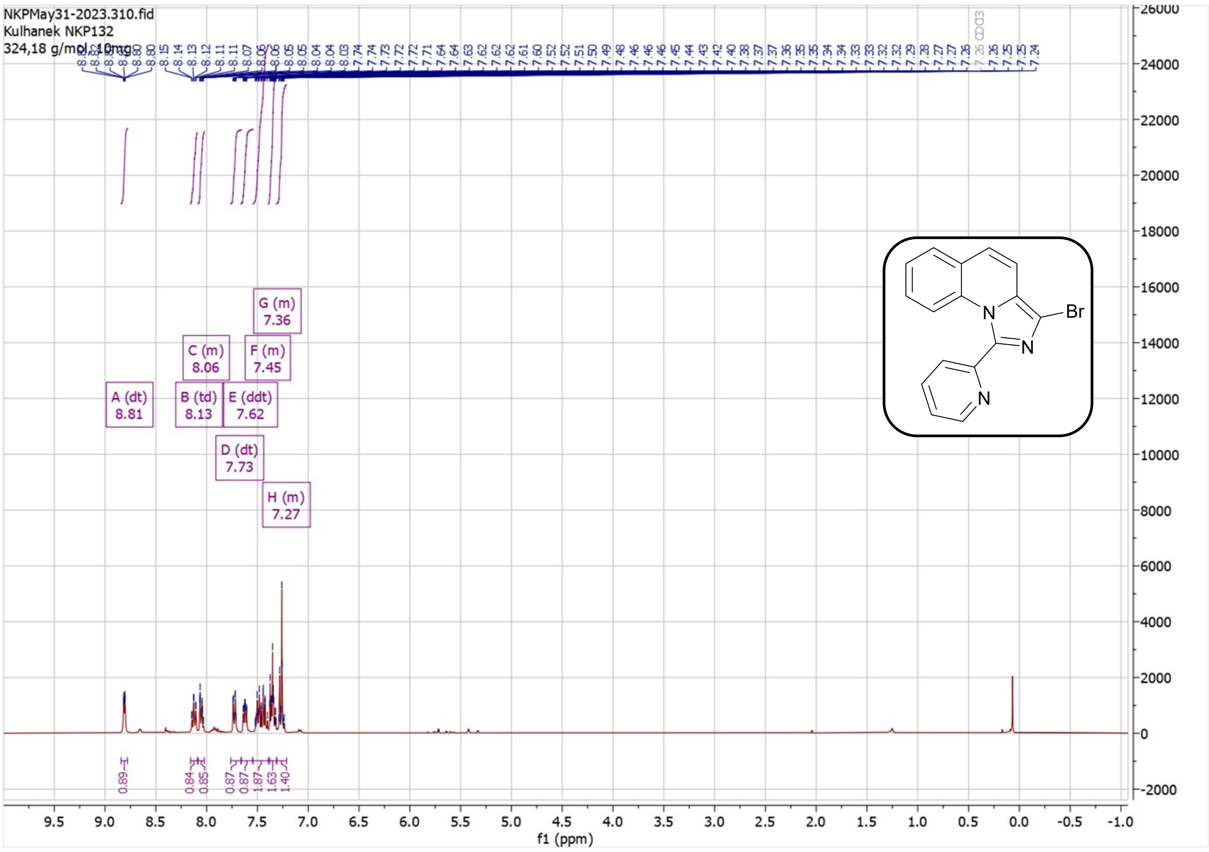

NKPMay31-2023.310.fid
Kulhanek NKP132
324,18 g/mol, 10mg
A (dt) 8.81
B (td) 8.13
C (m) 8.06
D (dt) 7.73
E (ddt) 7.62
F (m) 7.45
G (m) 7.36
H (m) 7.27
0.89
0.84
0.85
0.87
0.87
1.87
1.63
1.40
Br
N
N
N
f1 (ppm)

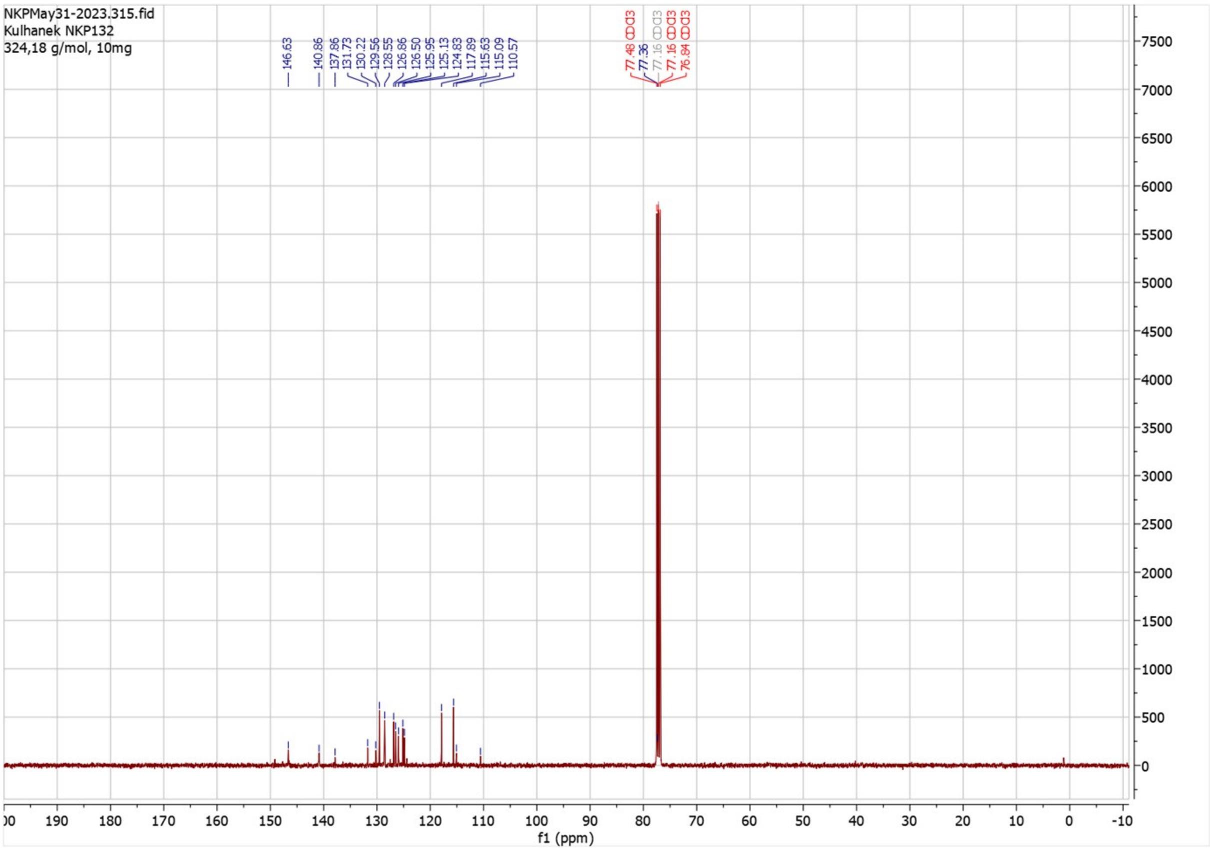

NKPMay31-2023.315.fid
Kulhanek NKP132
324,18 g/mol, 10mg
146.63
140.86
137.86
131.73
130.22
129.56
128.55
126.86
126.50
125.95
125.13
124.83
117.89
115.63
115.09
110.57
77.48 CDCl3
77.36
77.16 CDCl3
77.16 CDCl3
76.84 CDCl3
f1 (ppm)

Compound 6a

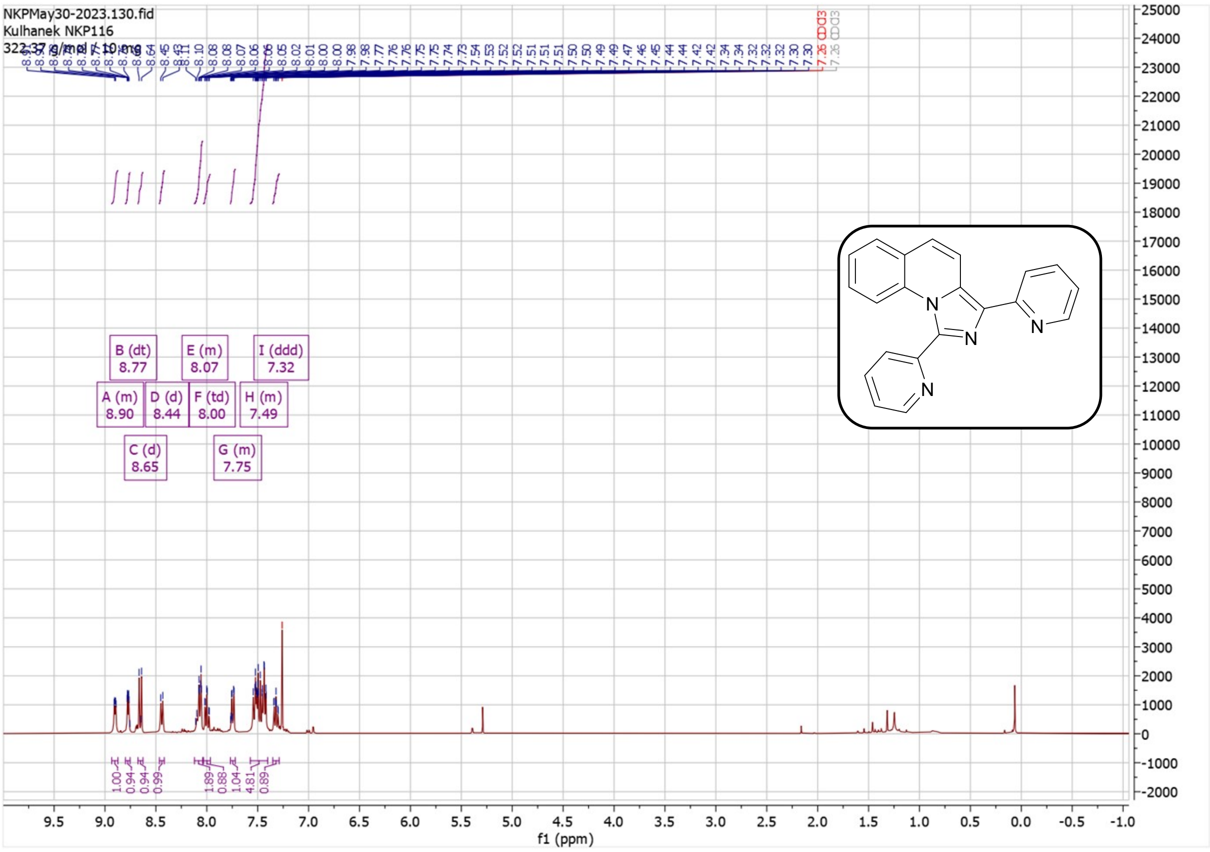

NKPMay30-2023.130.fid
Kulhanek NKP116
322.37 g/mol / 10 mg
A (m) 8.90
B (dt) 8.77
C (d) 8.65
D (d) 8.44
E (m) 8.07
F (td) 8.00
G (m) 7.75
H (m) 7.49
I (ddd) 7.32
1.00
0.94
0.94
0.99
1.89
0.88
1.04
4.81
0.89
N
f1 (ppm)

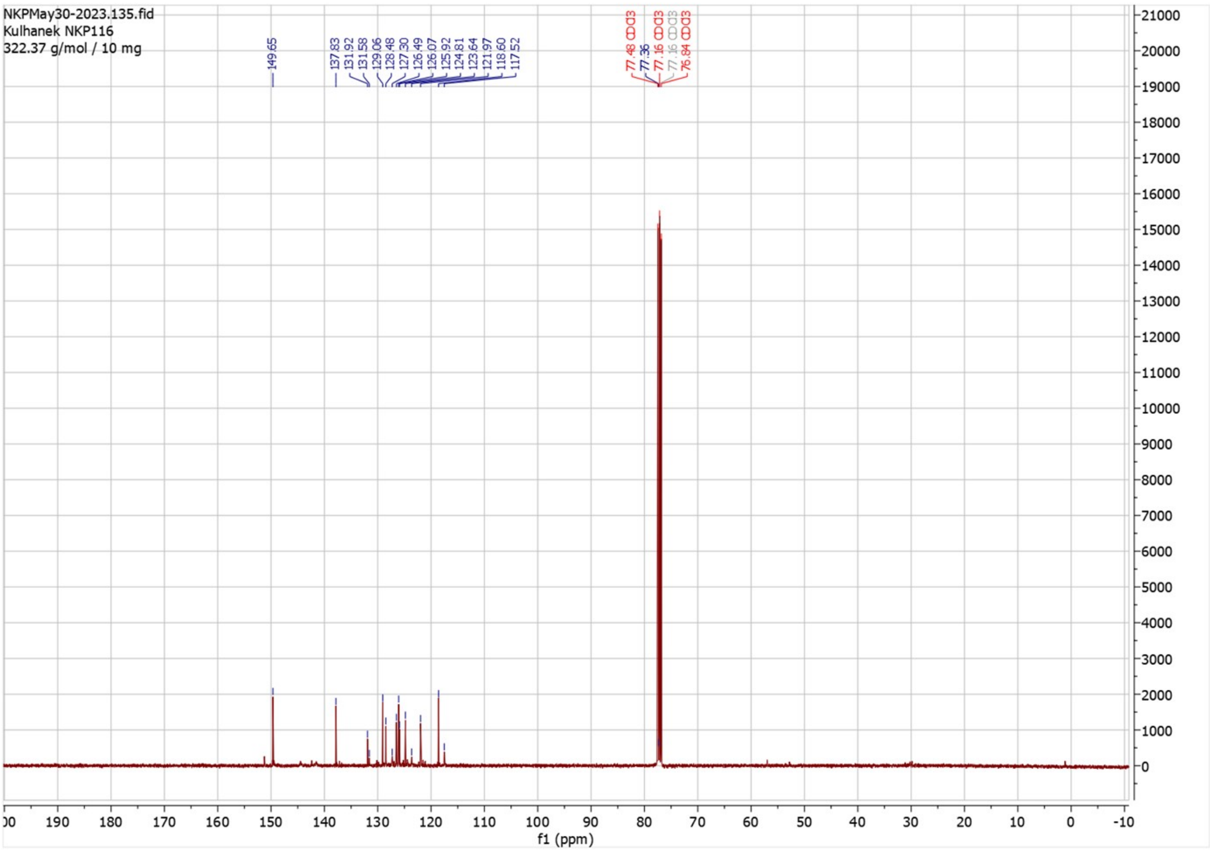

NKPMay30-2023.135.fid
Kulhanek NKP116
322.37 g/mol / 10 mg
149.65
137.83
131.92
131.58
129.06
128.48
127.30
126.49
126.07
125.92
124.81
123.64
121.97
118.60
117.52
77.48 CDCl3
77.36
77.16 CDCl3
76.84 CDCl3
f1 (ppm)

Compound 6b

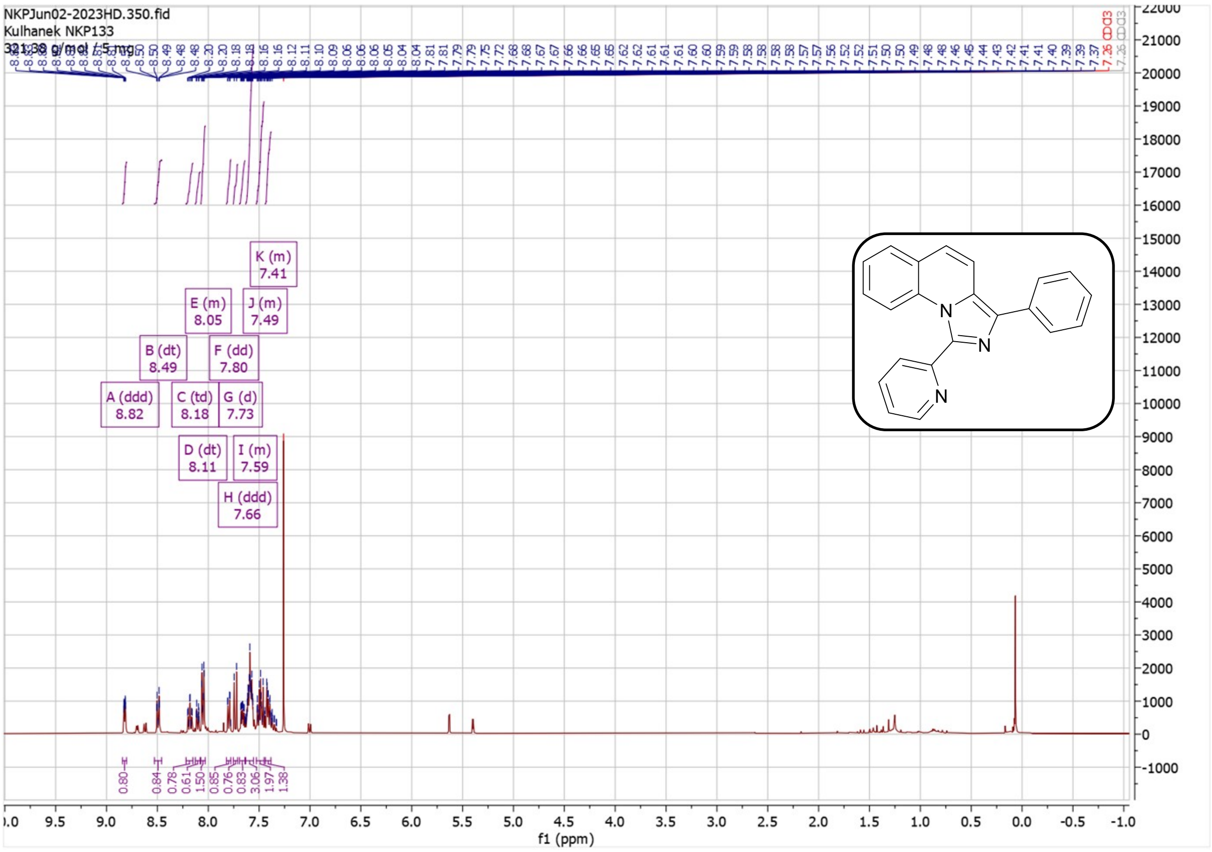
NKPJun02-2023HD.350.fid
Kulhanek NKP133
321.38 g/mol / 5 mg
A (ddd) 8.82
B (dt) 8.49
C (td) 8.18
D (dt) 8.11
E (m) 8.05
F (dd) 7.80
G (d) 7.73
H (ddd) 7.66
I (m) 7.59
J (m) 7.49
K (m) 7.41
f1 (ppm)

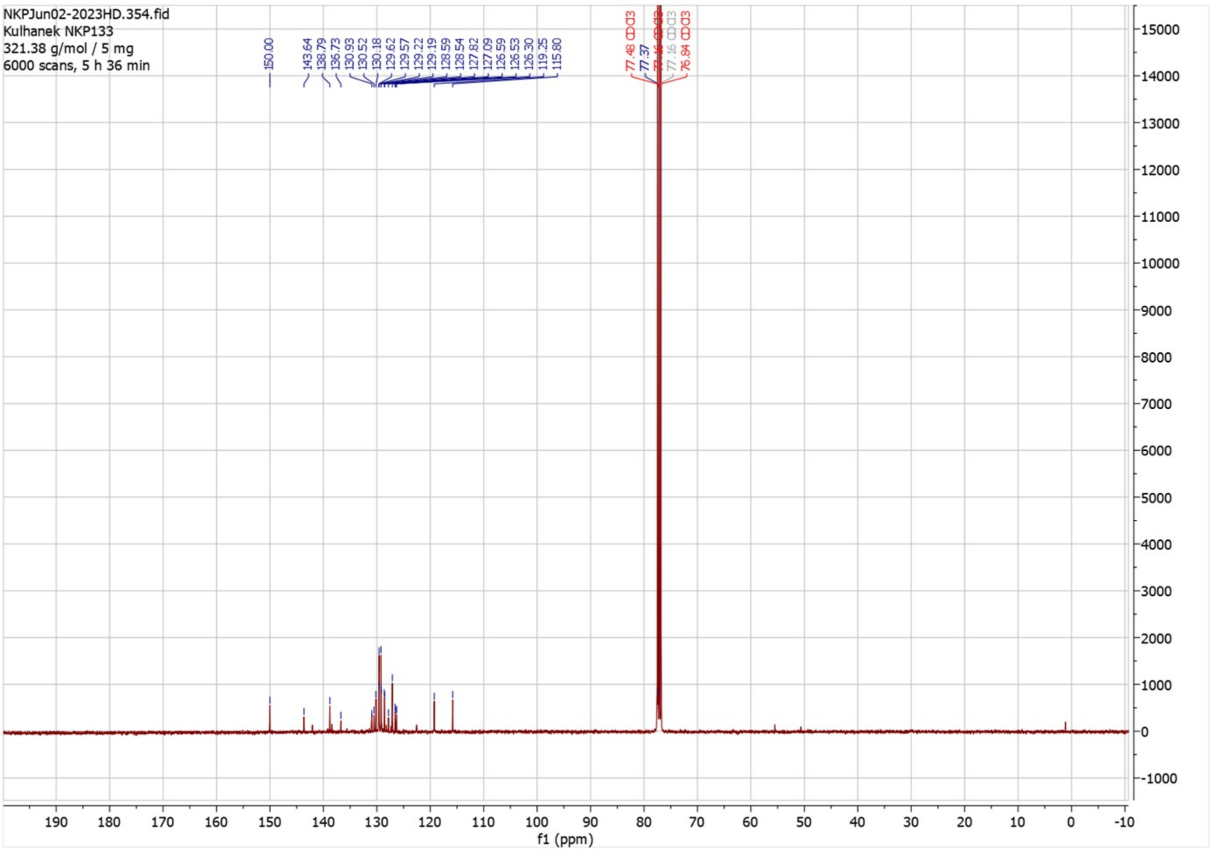
NKPJun02-2023HD.354.fid
Kulhanek NKP133
321.38 g/mol / 5 mg
6000 scans, 5 h 36 min
150.00
143.64
138.79
136.73
130.93
130.52
130.18
129.62
129.57
129.22
129.19
128.59
128.54
127.82
127.09
126.59
126.53
126.30
119.25
115.80
f1 (ppm)

Compound 7a

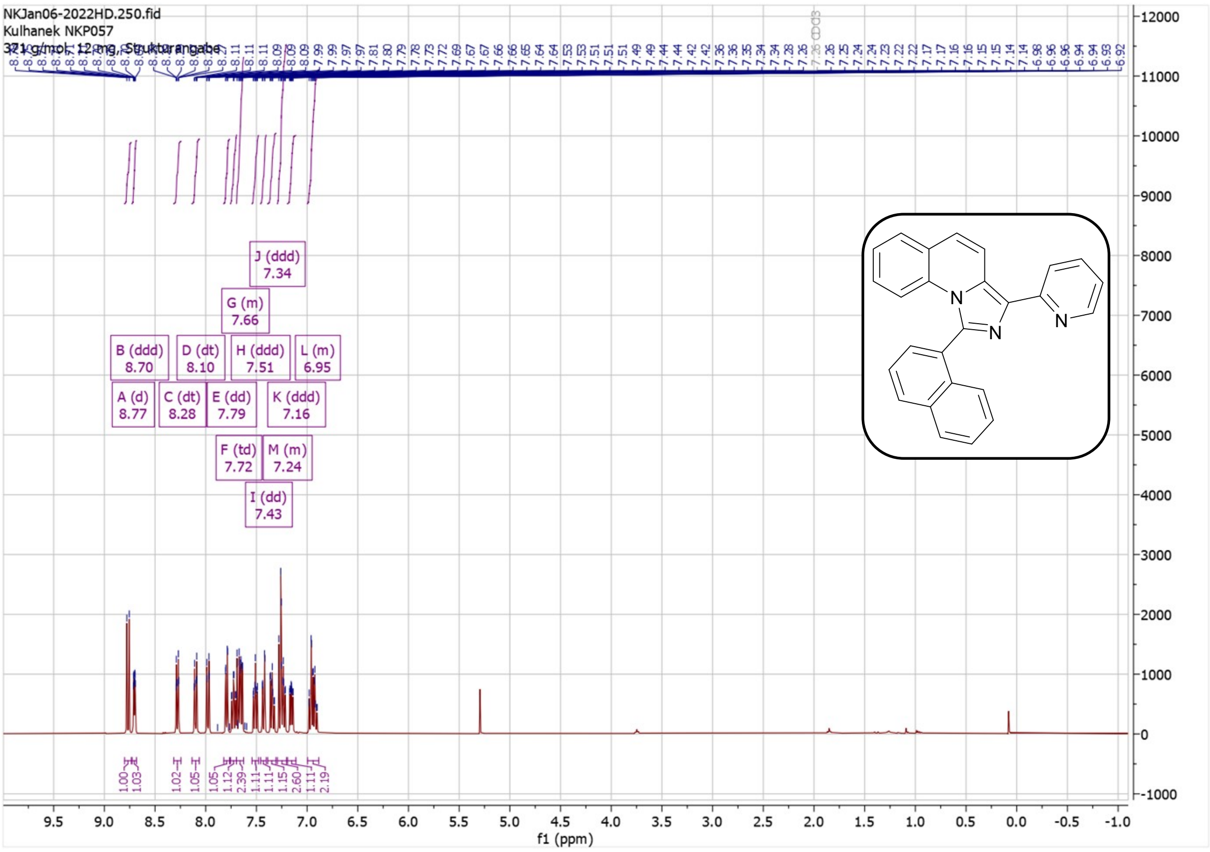
NKJan06-2022HD.250.fid
Kulhanek NKP057
371 g/mol, 12 mg, Strukturangabe
J (ddd) 7.34
G (m) 7.66
B (ddd) 8.70
D (dt) 8.10
H (ddd) 7.51
L (m) 6.95
A (d) 8.77
C (dt) 8.28
E (dd) 7.79
K (ddd) 7.16
F (td) 7.72
M (m) 7.24
I (dd) 7.43
N
N
N
f1 (ppm)

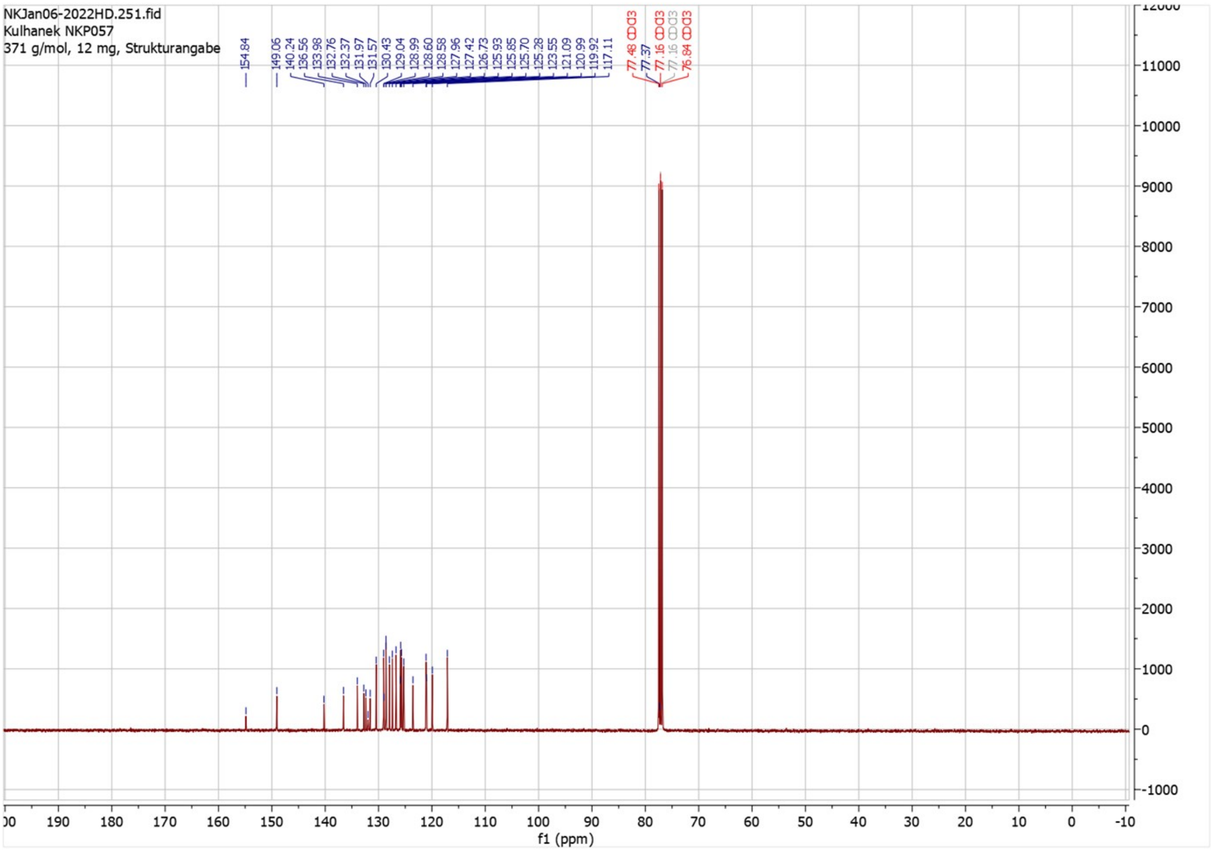
NKJan06-2022HD.251.fid
Kulhanek NKP057
371 g/mol, 12 mg, Strukturangabe
154.84
149.06
140.24
136.56
133.98
132.76
132.37
131.97
131.57
130.43
129.04
128.99
128.60
128.58
127.96
127.42
126.73
125.93
125.85
125.70
125.28
123.55
121.09
120.99
119.92
117.11
77.48 CDCl3
77.37
77.16 CDCl3
76.84 CDCl3
f1 (ppm)

Compound 8a

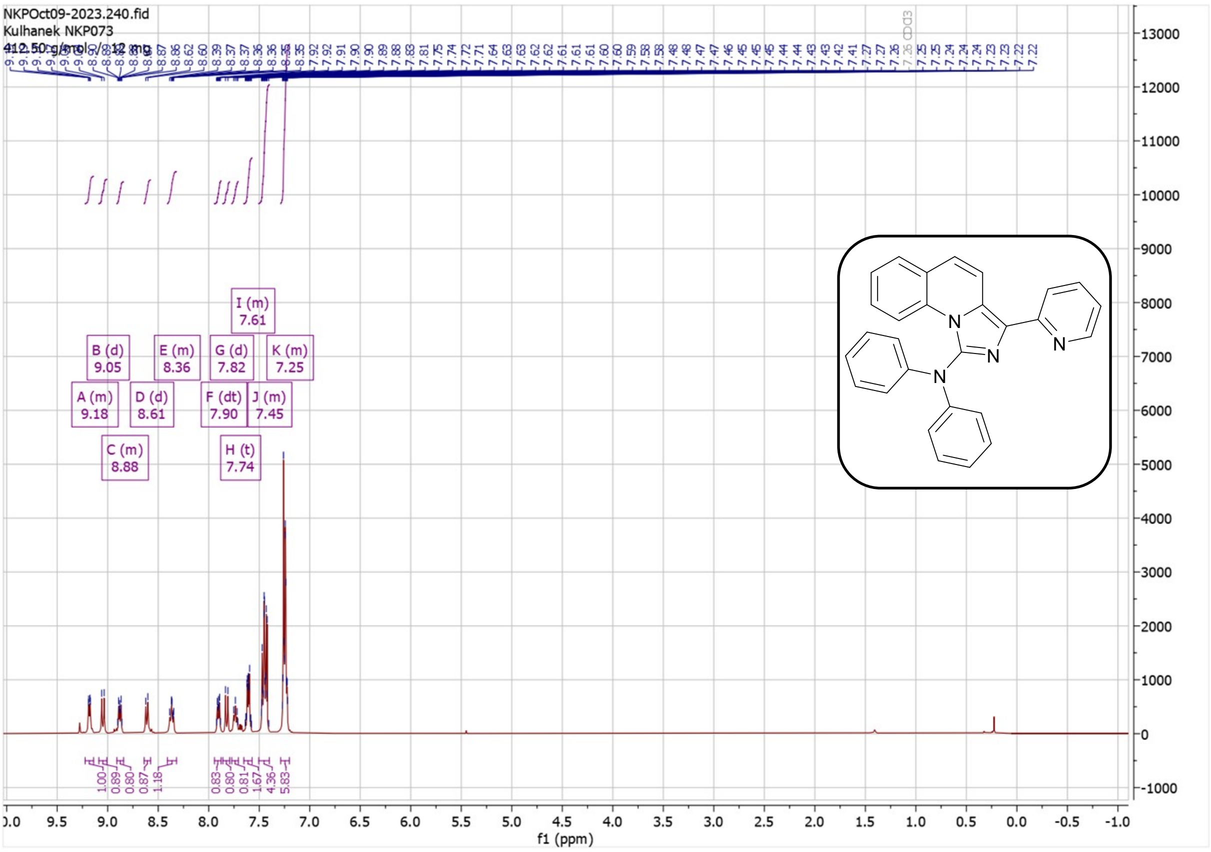
NKPOct09-2023.240.fid
Kulhanek NKP073
412.50 g/mol / 12 mg
A (m) 9.18
B (d) 9.05
C (m) 8.88
D (d) 8.61
E (m) 8.36
F (dt) 7.90
G (d) 7.82
H (t) 7.74
I (m) 7.61
J (m) 7.45
K (m) 7.25
1.00
0.89
0.80
0.87
1.18
0.83
0.80
0.81
1.67
4.36
5.83
f1 (ppm)

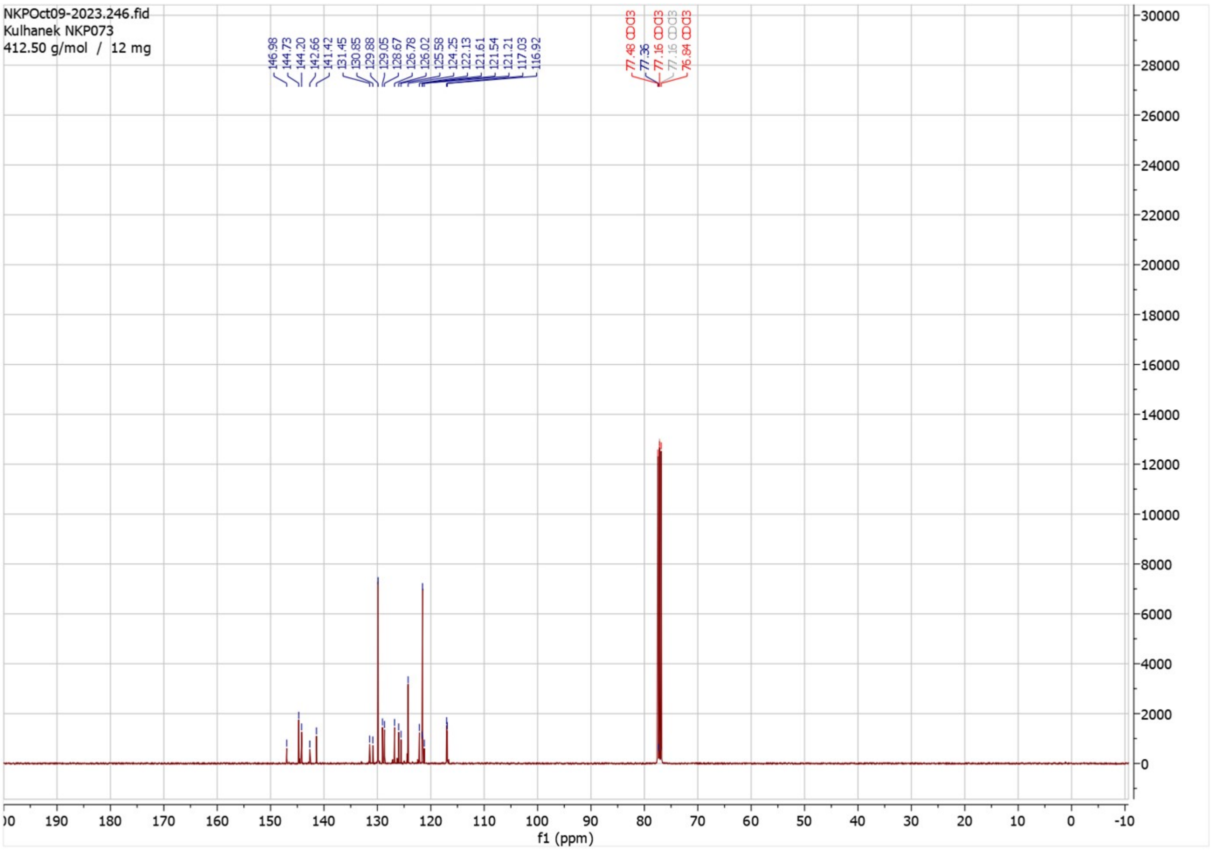
NKPOct09-2023.246.fid
Kulhanek NKP073
412.50 g/mol / 12 mg
146.98
144.73
144.20
142.66
141.42
131.45
130.85
129.88
129.05
128.67
126.78
126.02
125.58
124.25
122.13
121.61
121.54
121.21
117.03
116.92
77.48 CDCl3
77.36
77.16 CDCl3
77.16 CDCl3
76.84 CDCl3
f1 (ppm)

Compound C1

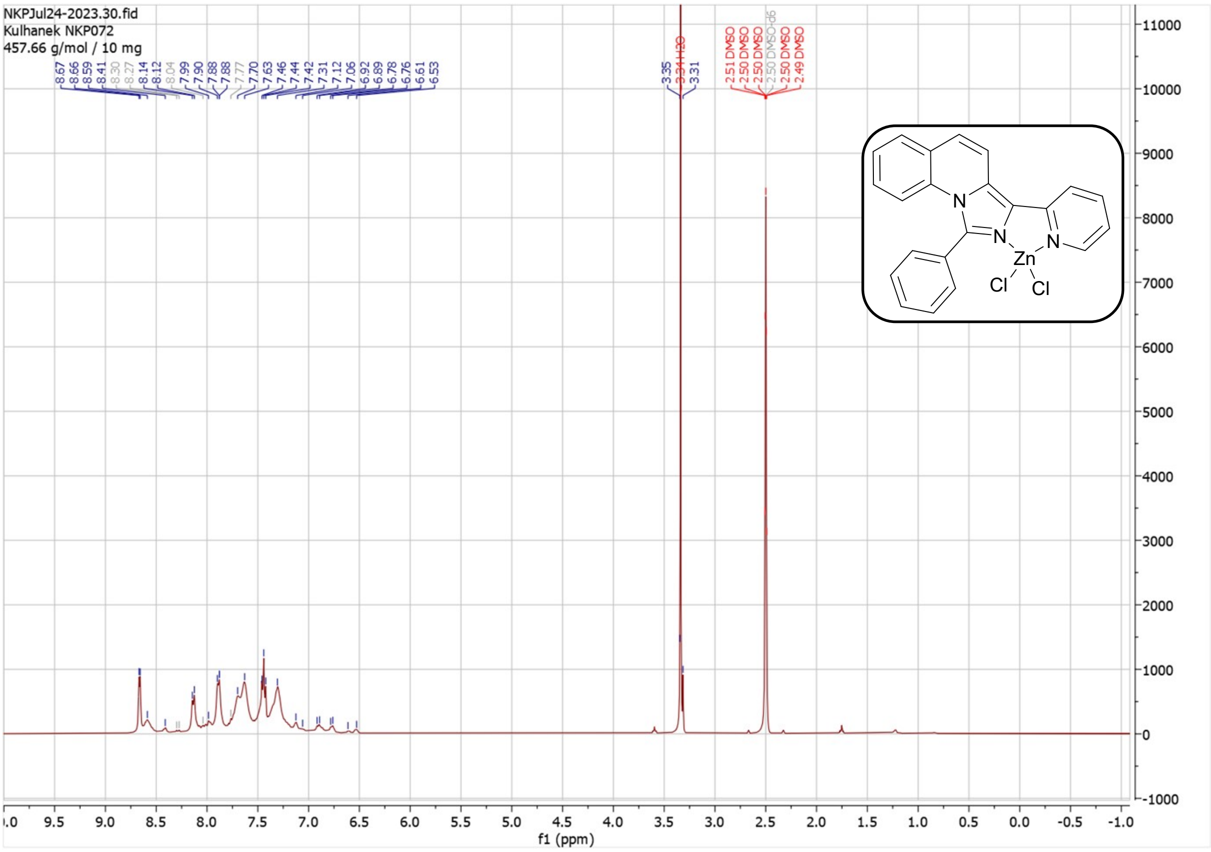
NKPJul24-2023.30.fid
Kulhanek NKP072
457.66 g/mol / 10 mg
8.67 8.66 8.59 8.41 8.30 8.27 8.14 8.12 8.04 7.99 7.90 7.88 7.88 7.77 7.70 7.63 7.46 7.44 7.42 7.31 7.12 7.06 6.92 6.89 6.78 6.76 6.61 6.53
3.35 3.34 H2O 3.31
2.51 DMSO 2.50 DMSO 2.50 DMSO 2.50 DMSO-d6 2.50 DMSO 2.49 DMSO
N
N
N
Zn
Cl
Cl
f1 (ppm)

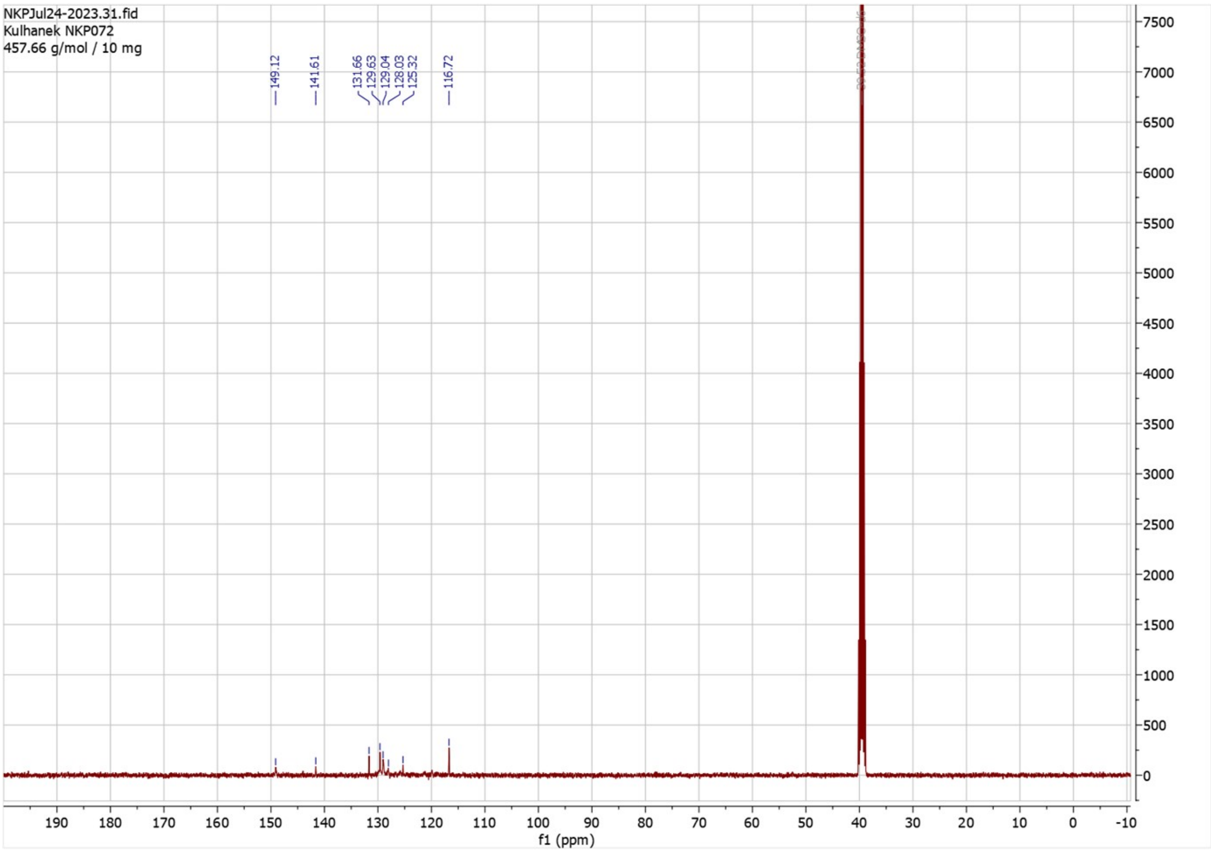
NKPJul24-2023.31.fid
Kulhanek NKP072
457.66 g/mol / 10 mg
149.12
141.61
131.66 129.63 129.04 128.03 125.32
116.72
f1 (ppm)

Compound C2

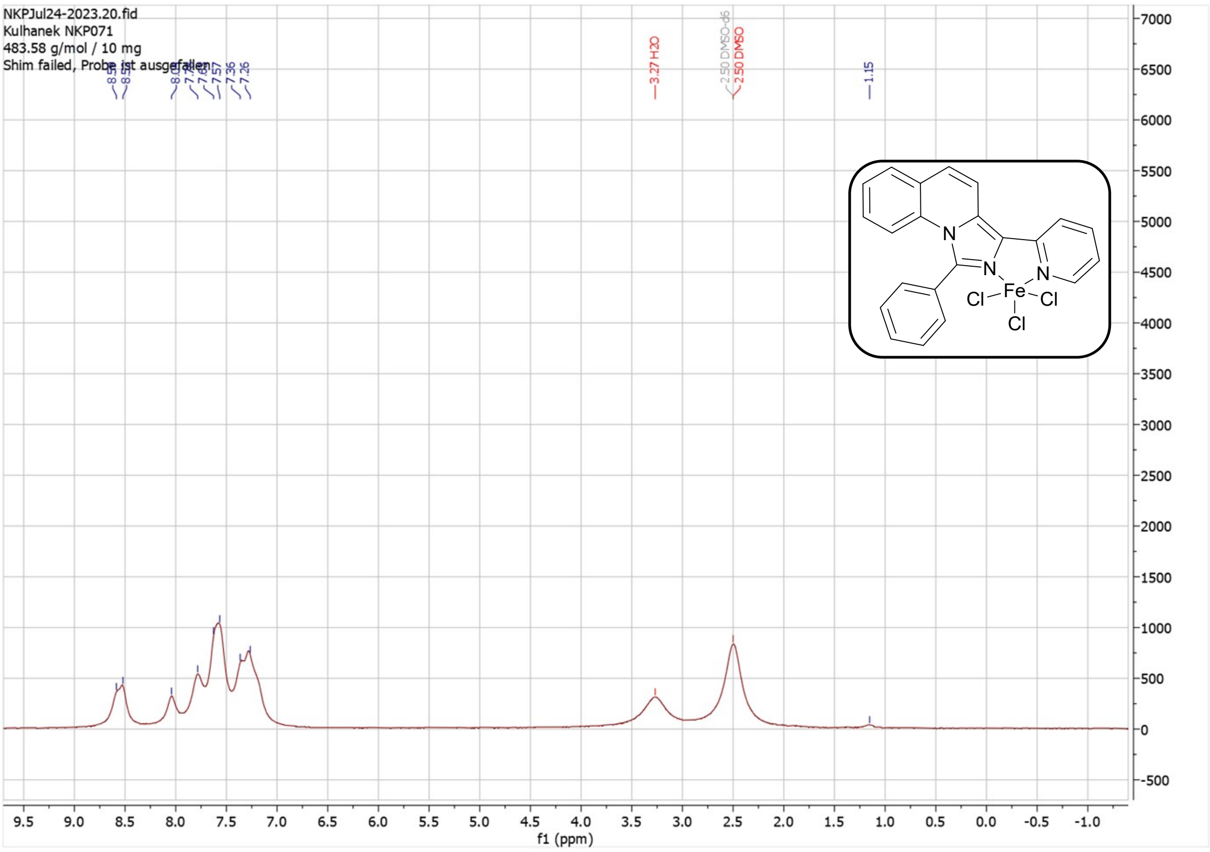

NKPJul24-2023.20.fid
Kulhanek NKP071
483.58 g/mol / 10 mg
Shim failed, Probe ist ausgefallen
7.57
7.36
7.26
3.27 H2O
2.50 DMSO-d6
2.50 DMSO
1.15
N
N
N
Fe
Cl
Cl
Cl
f1 (ppm)

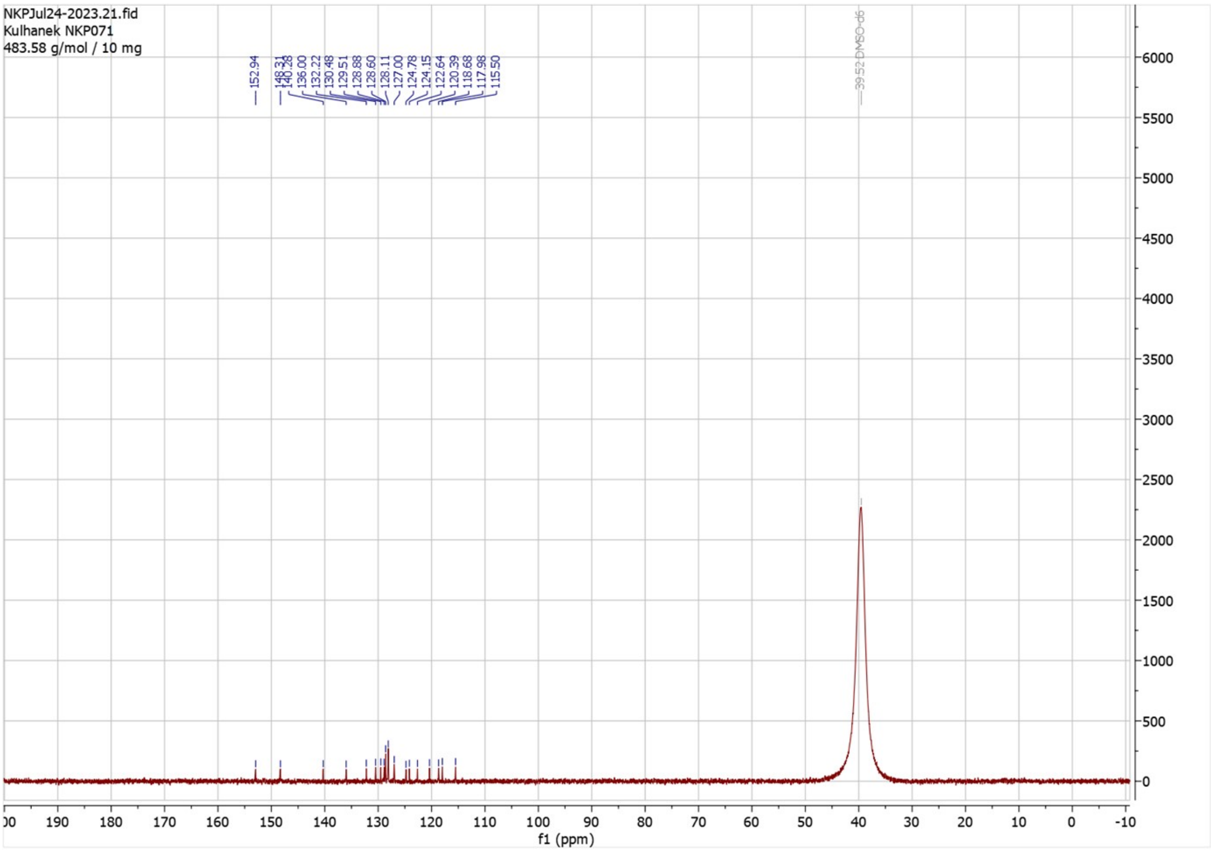

NKPJul24-2023.21.fid
Kulhanek NKP071
483.58 g/mol / 10 mg
152.94
148.31
136.00
132.22
130.48
129.51
128.88
128.60
128.11
127.00
124.78
124.15
122.64
120.39
118.68
117.98
115.50
39.52 DMSO-d6
f1 (ppm)

Compound C3

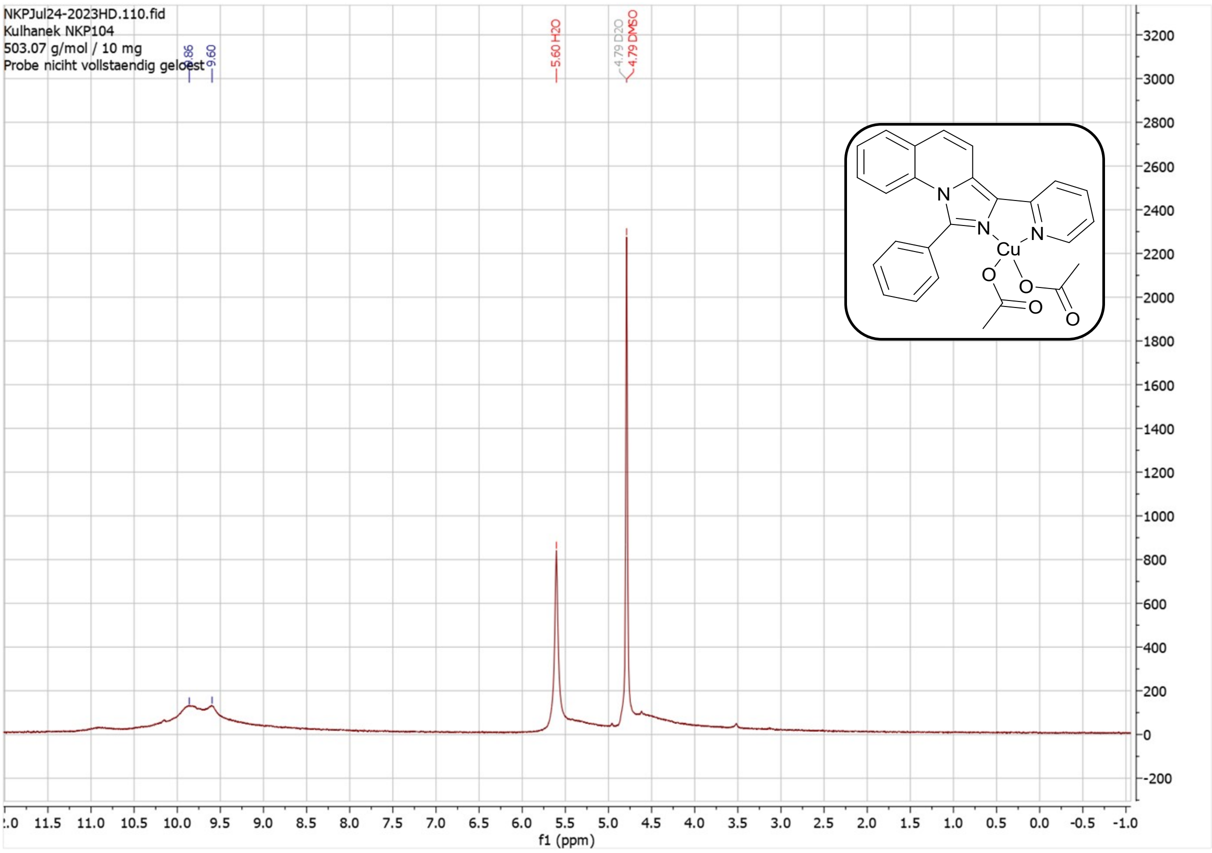

NKPJul24-2023HD.110.fid
Kulhanek NKP104
503.07 g/mol / 10 mg
Probe niciht vollstaendig geloest
9.86
9.60
5.60 H2O
4.79 D2O
4.79 DMSO
N
N
N
Cu
O
O
O
O
f1 (ppm)

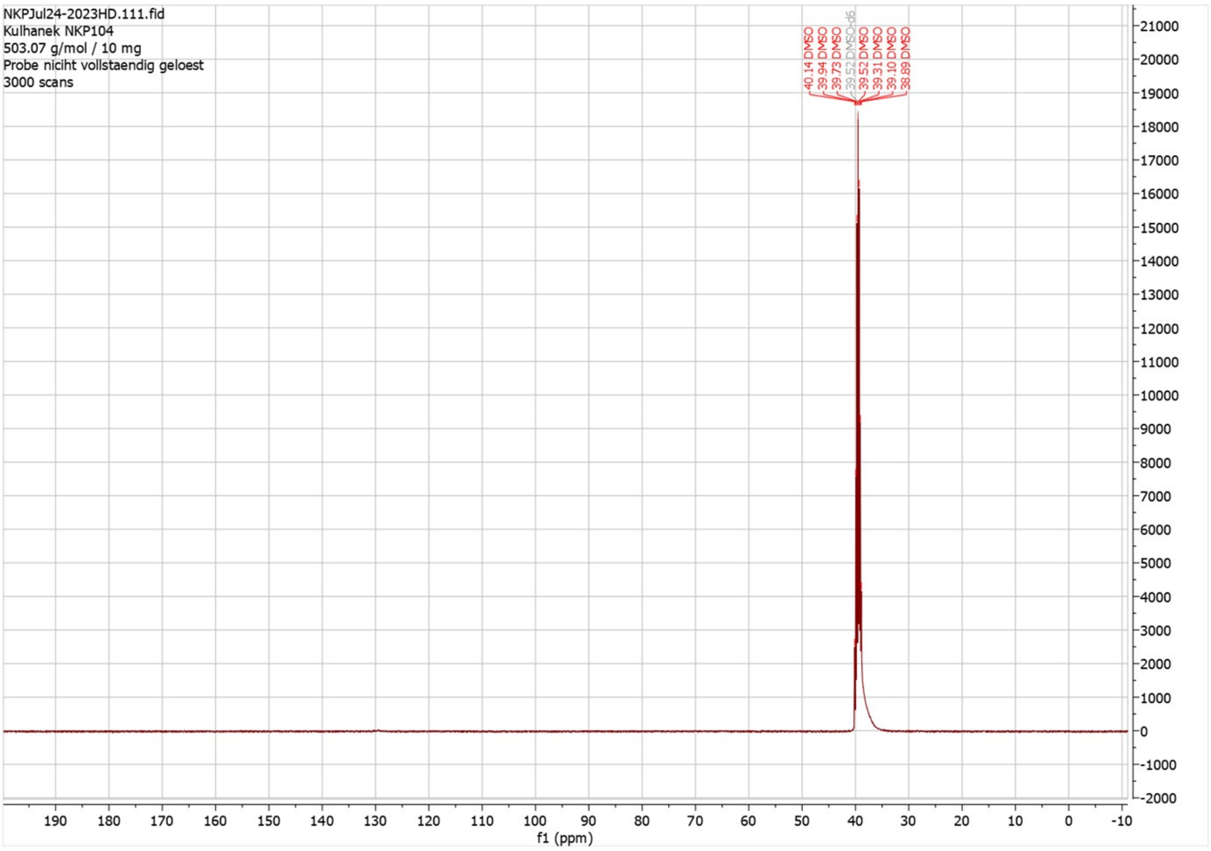

NKPJul24-2023HD.111.fid
Kulhanek NKP104
503.07 g/mol / 10 mg
Probe niciht vollstaendig geloest
3000 scans
40.14 DMSO
39.94 DMSO
39.73 DMSO
39.52 DMSO-d6
39.52 DMSO
39.31 DMSO
39.10 DMSO
38.89 DMSO
f1 (ppm)

Compound C4

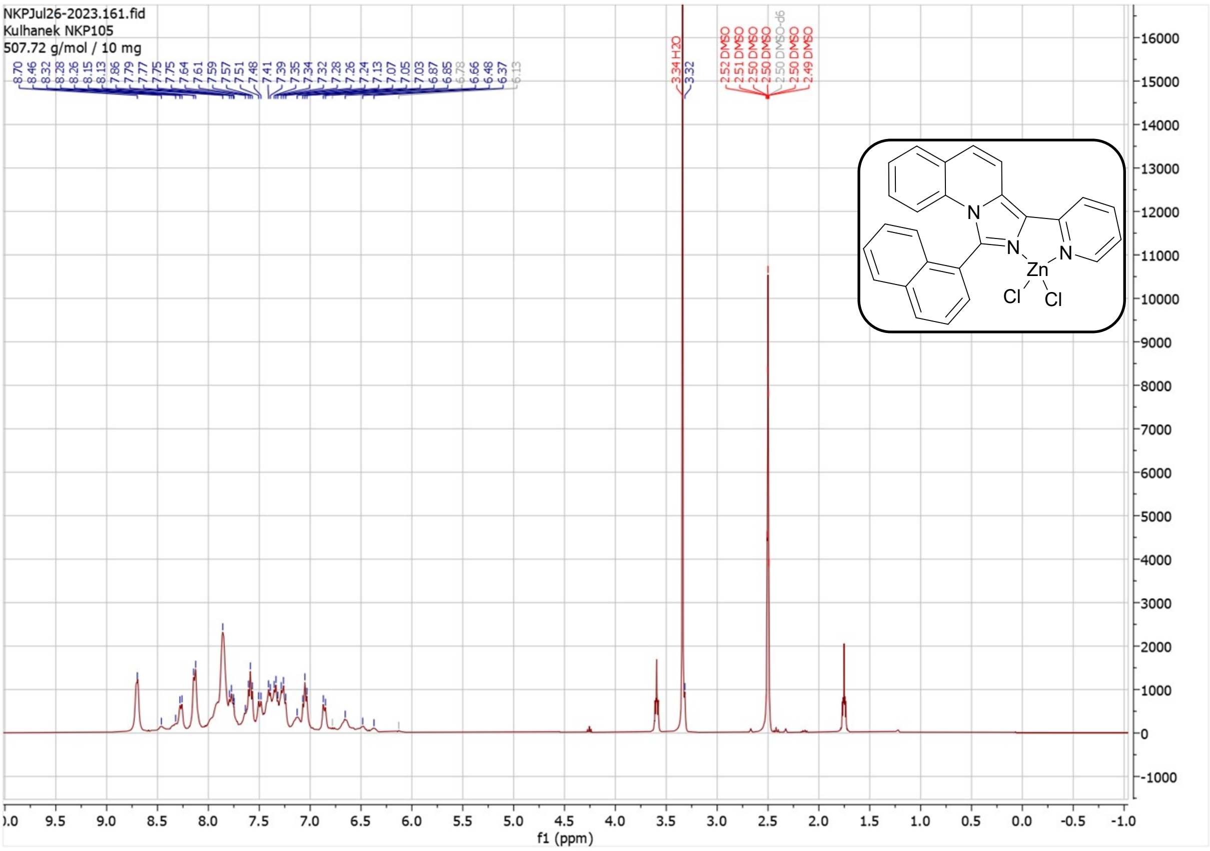
NKPJul26-2023.161.fid
Kulhanek NKP105
507.72 g/mol / 10 mg
8.70 8.46 8.32 8.28 8.26 8.15 8.13 7.86 7.79 7.77 7.75 7.75 7.64 7.61 7.59 7.57 7.51 7.48 7.41 7.39 7.35 7.34 7.32 7.28 7.26 7.24 7.13 7.07 7.05 7.03 6.87 6.85 6.78 6.66 6.48 6.37 6.13
3.34 H2O
3.32
2.52 DMSO 2.51 DMSO 2.50 DMSO 2.50 DMSO 2.50 DMSO-d6 2.50 DMSO 2.49 DMSO
N
N
N
Zn
Cl
Cl
f1 (ppm)

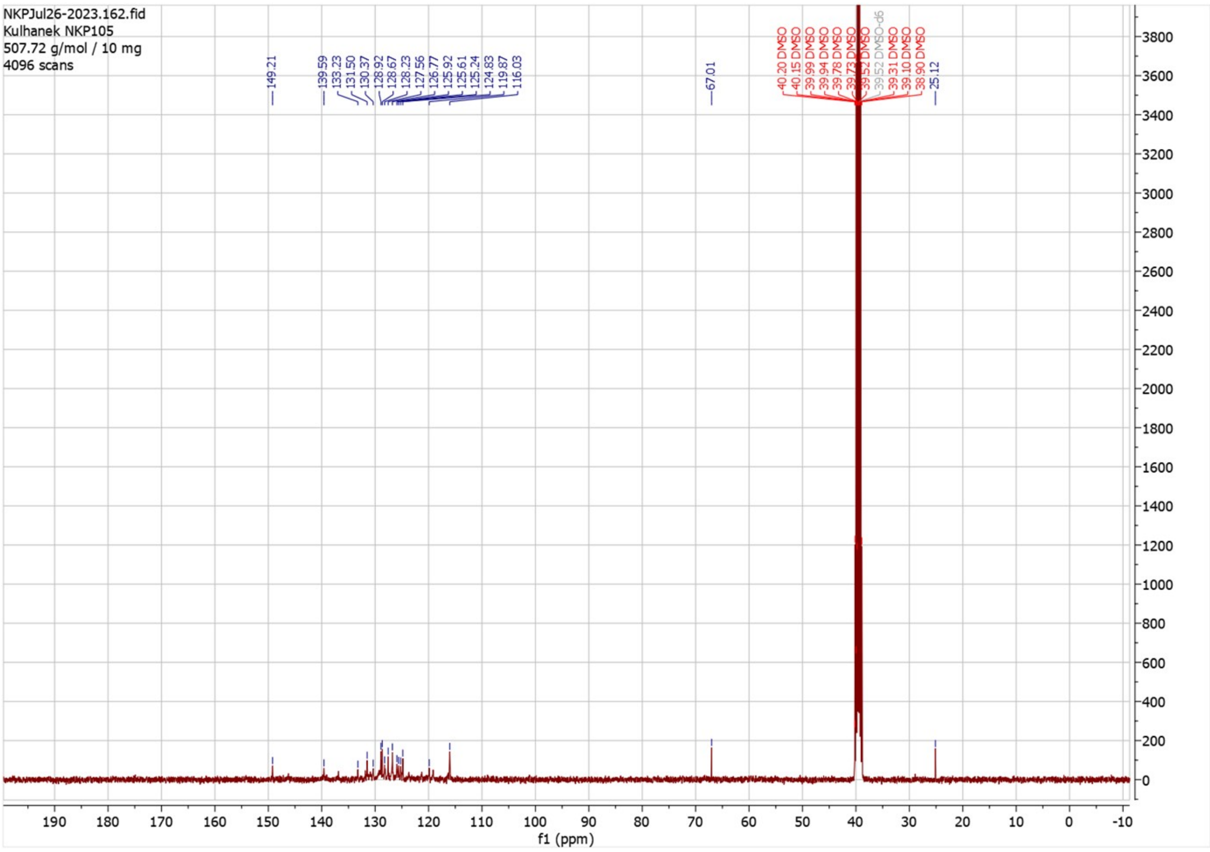
NKPJul26-2023.162.fid
Kulhanek NKP105
507.72 g/mol / 10 mg
4096 scans
149.21
139.59 133.23 131.50 130.37 128.92 128.67 128.23 127.56 126.77 125.92 125.61 125.24 124.83 119.87 116.03
67.01
40.20 DMSO 40.15 DMSO 39.99 DMSO 39.94 DMSO 39.78 DMSO 39.73 DMSO 39.52 DMSO-d6 39.31 DMSO 39.10 DMSO 38.90 DMSO
25.12
f1 (ppm)

Compound C5

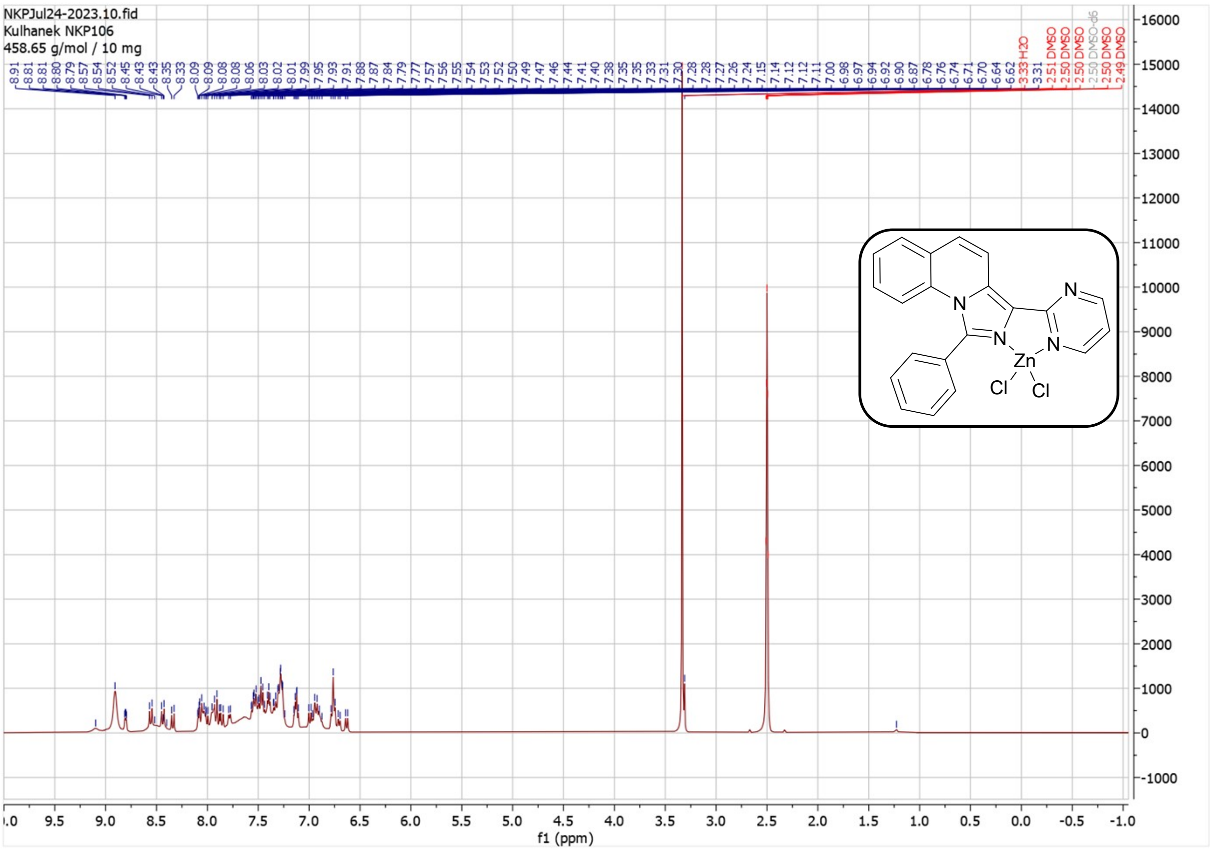
NKPJul24-2023.10.fid
Kulhanek NKP106
458.65 g/mol / 10 mg
8.91 8.81 8.80 8.79 8.57 8.54 8.52 8.45 8.43 8.35 8.33 8.09 8.09 8.08 8.08 8.06 8.03 8.02 8.01 7.99 7.95 7.93 7.91 7.88 7.87 7.84 7.79 7.77 7.57 7.56 7.55 7.54 7.53 7.52 7.50 7.49 7.47 7.46 7.44 7.41 7.40 7.38 7.35 7.35 7.33 7.31 7.30 7.28 7.28 7.27 7.26 7.24 7.15 7.14 7.12 7.11 7.00 6.98 6.97 6.94 6.92 6.90 6.87 6.78 6.76 6.74 6.71 6.70 6.64 6.62
3.33 H2O
3.31
2.51 DMSO
2.50 DMSO
2.50 DMSO
2.50 DMSO-d6
2.50 DMSO
2.49 DMSO
N
N
N
N
Zn
Cl
Cl
f1 (ppm)

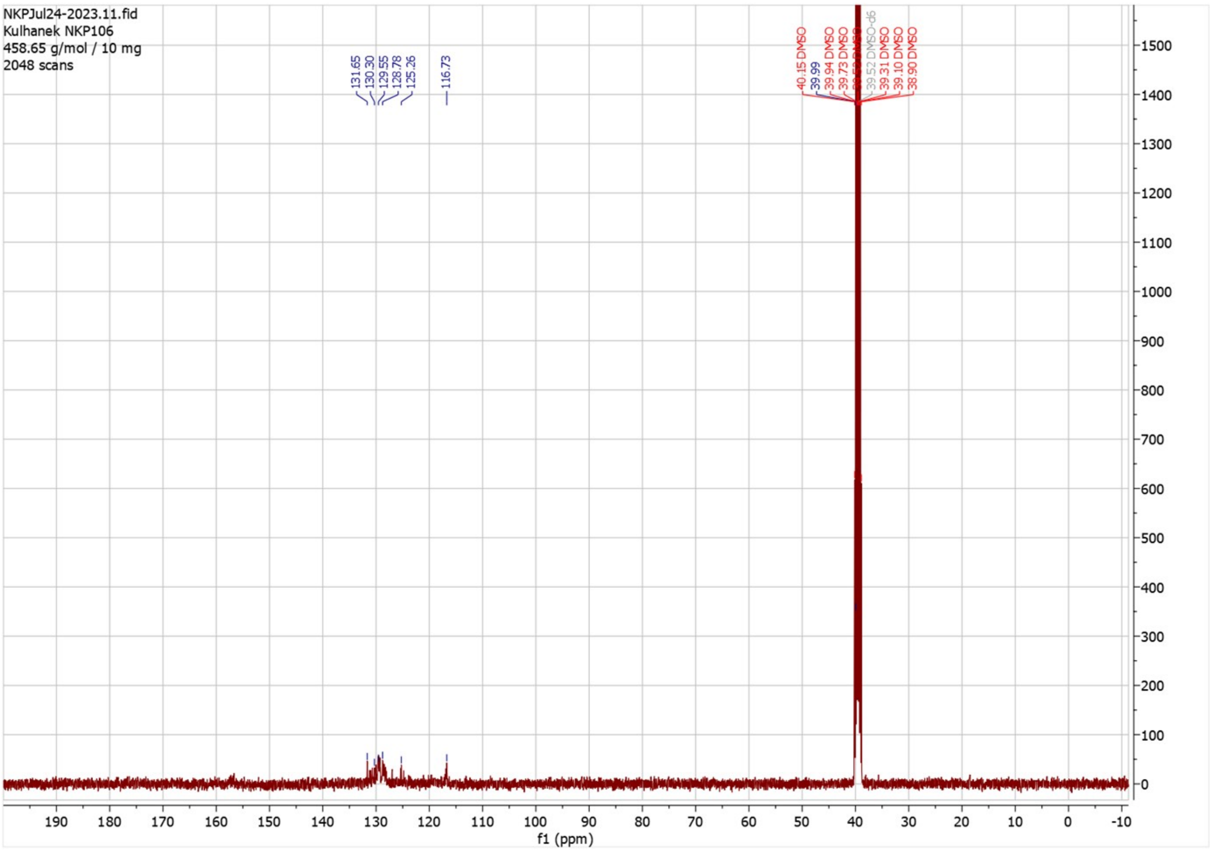
NKPJul24-2023.11.fid
Kulhanek NKP106
458.65 g/mol / 10 mg
2048 scans
131.65 130.30 129.55 128.78 125.26
116.73
40.15 DMSO
39.99
39.94 DMSO
39.73 DMSO
39.52 DMSO-d6
39.31 DMSO
39.10 DMSO
38.90 DMSO
f1 (ppm)

Compound C6

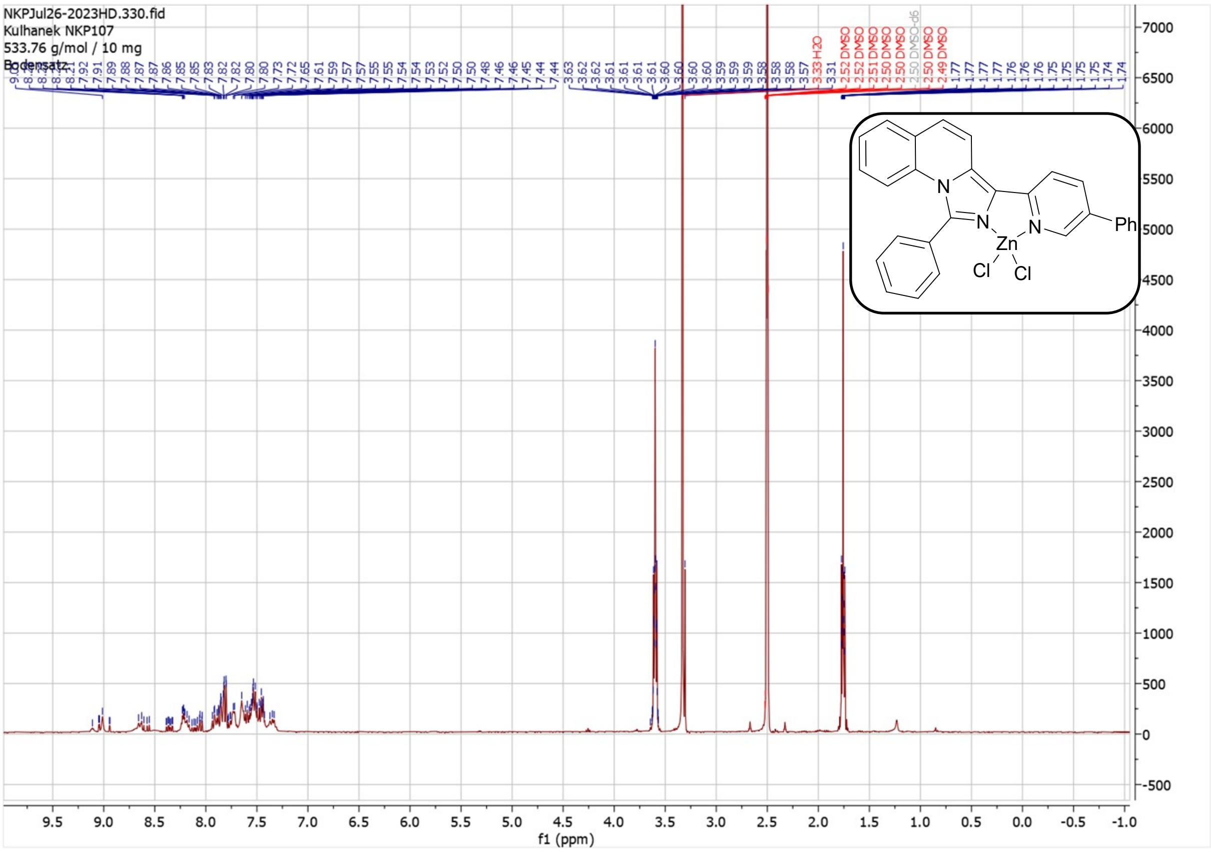
NKPJul26-2023HD.330.fid
Kulhanek NKP107
533.76 g/mol / 10 mg
Bodensatz
3.33 H2O
2.50 DMSO-d6
Zn
Cl
Cl
Ph
f1 (ppm)

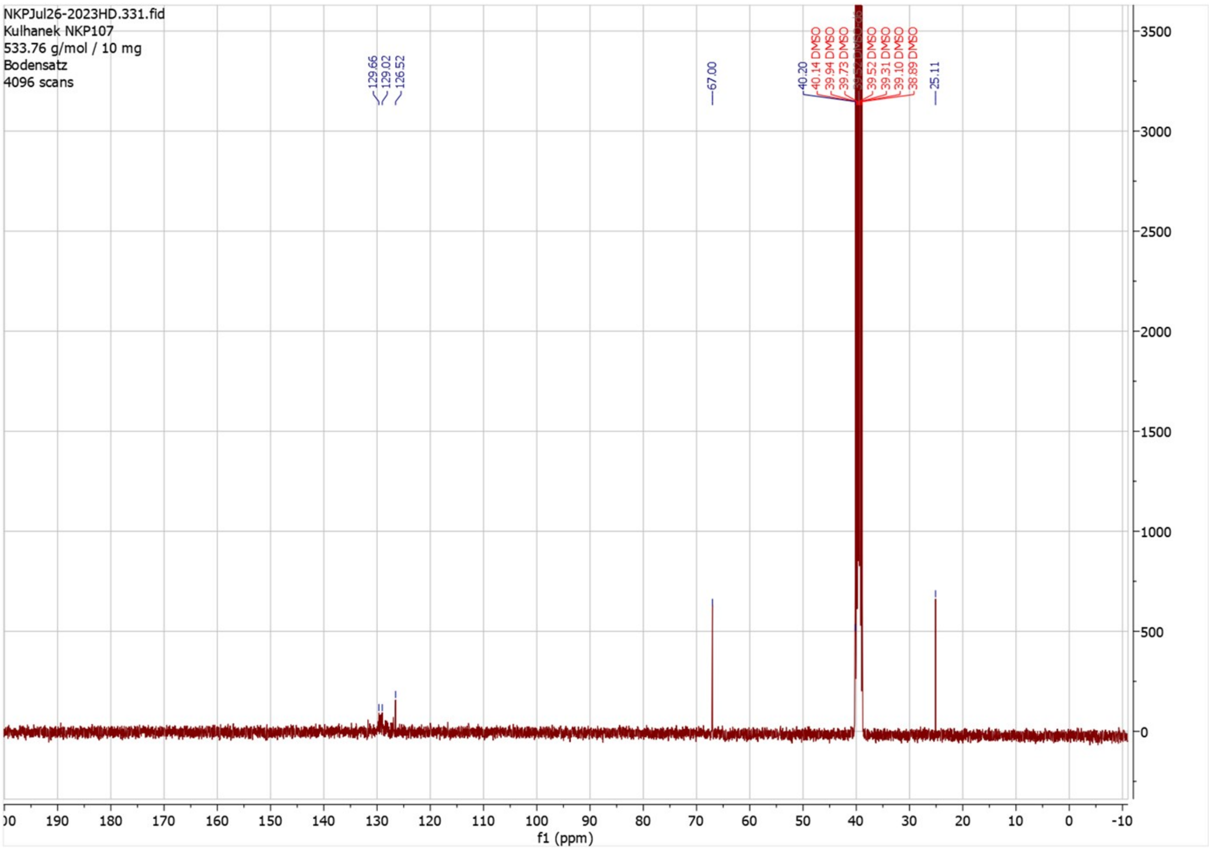
NKPJul26-2023HD.331.fid
Kulhanek NKP107
533.76 g/mol / 10 mg
Bodensatz
4096 scans
129.66
129.02
126.52
67.00
40.20
40.14 DMSO
39.94 DMSO
39.73 DMSO
39.52 DMSO
39.31 DMSO
39.10 DMSO
38.89 DMSO
25.11
f1 (ppm)

Compound C7

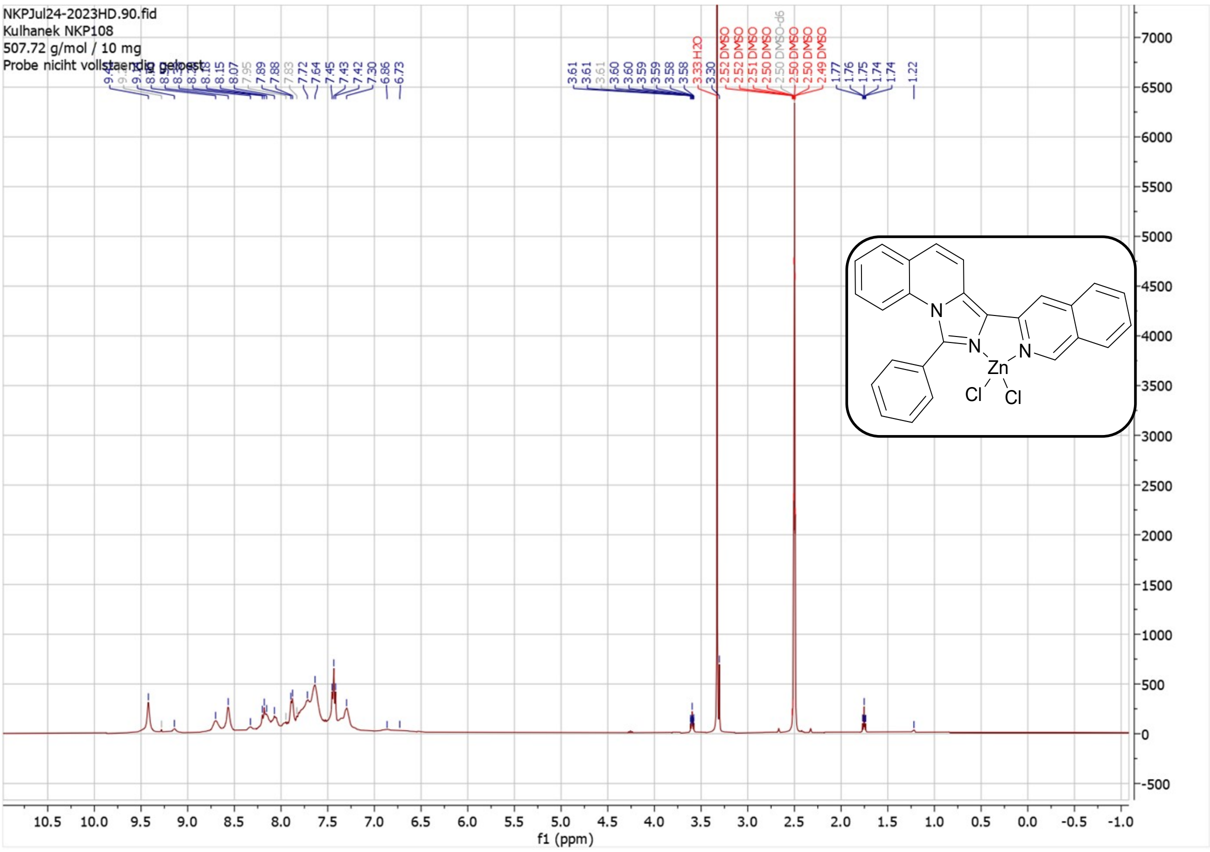
NKPJul24-2023HD.90.fid
Kulhanek NKP108
507.72 g/mol / 10 mg
Probe niciht vollstaendig geloest
8.15
8.07
7.95
7.89
7.88
7.83
7.72
7.64
7.45
7.43
7.42
7.30
6.86
6.73
3.61
3.61
3.61
3.60
3.60
3.59
3.59
3.58
3.58
3.33 H2O
3.30
2.52 DMSO
2.52 DMSO
2.51 DMSO
2.50 DMSO
2.50 DMSO-d6
2.50 DMSO
2.50 DMSO
2.49 DMSO
1.77
1.76
1.75
1.74
1.22
N
N
N
Zn
Cl
Cl
f1 (ppm)

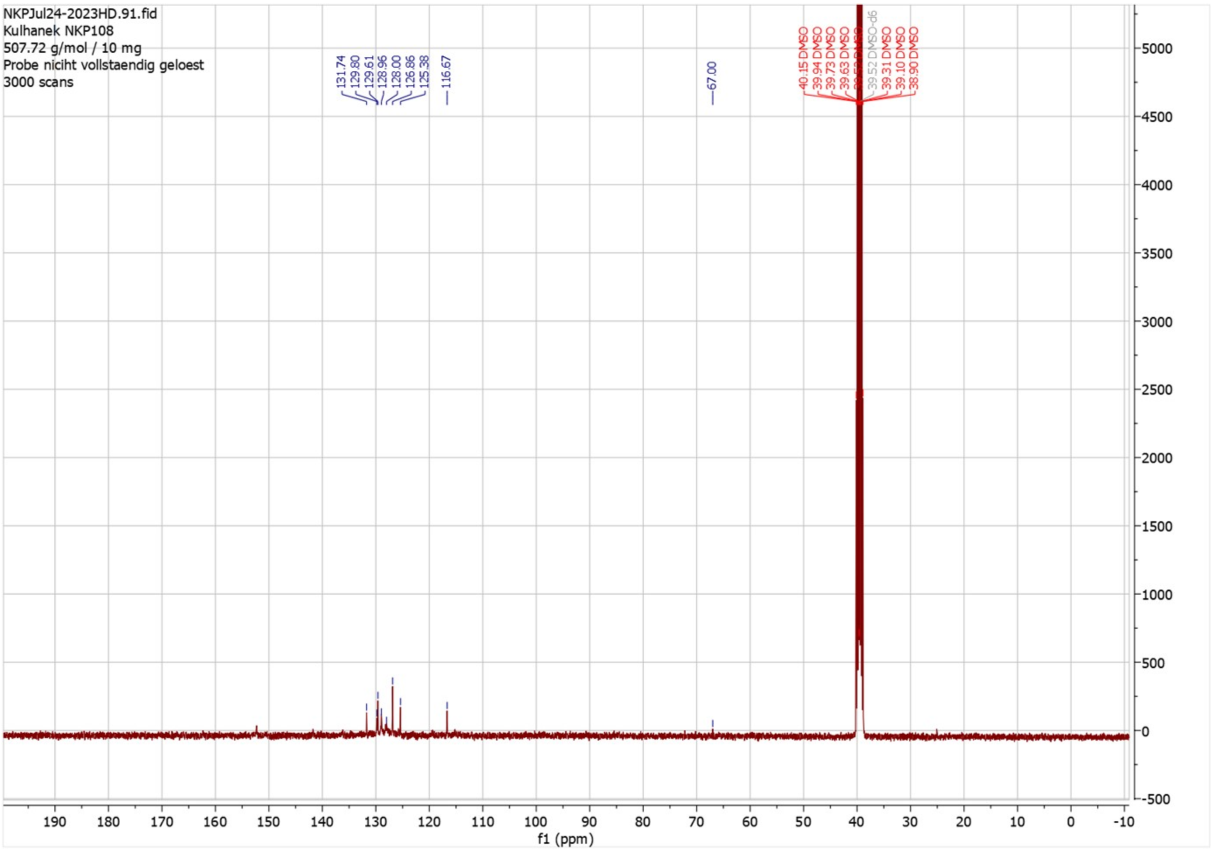
NKPJul24-2023HD.91.fid
Kulhanek NKP108
507.72 g/mol / 10 mg
Probe niciht vollstaendig geloest
3000 scans
131.74
129.80
129.61
128.96
128.00
126.86
125.38
116.67
67.00
40.15 DMSO
39.94 DMSO
39.73 DMSO
39.63 DMSO
39.52 DMSO-d6
39.31 DMSO
39.10 DMSO
38.90 DMSO
f1 (ppm)

Compound C8

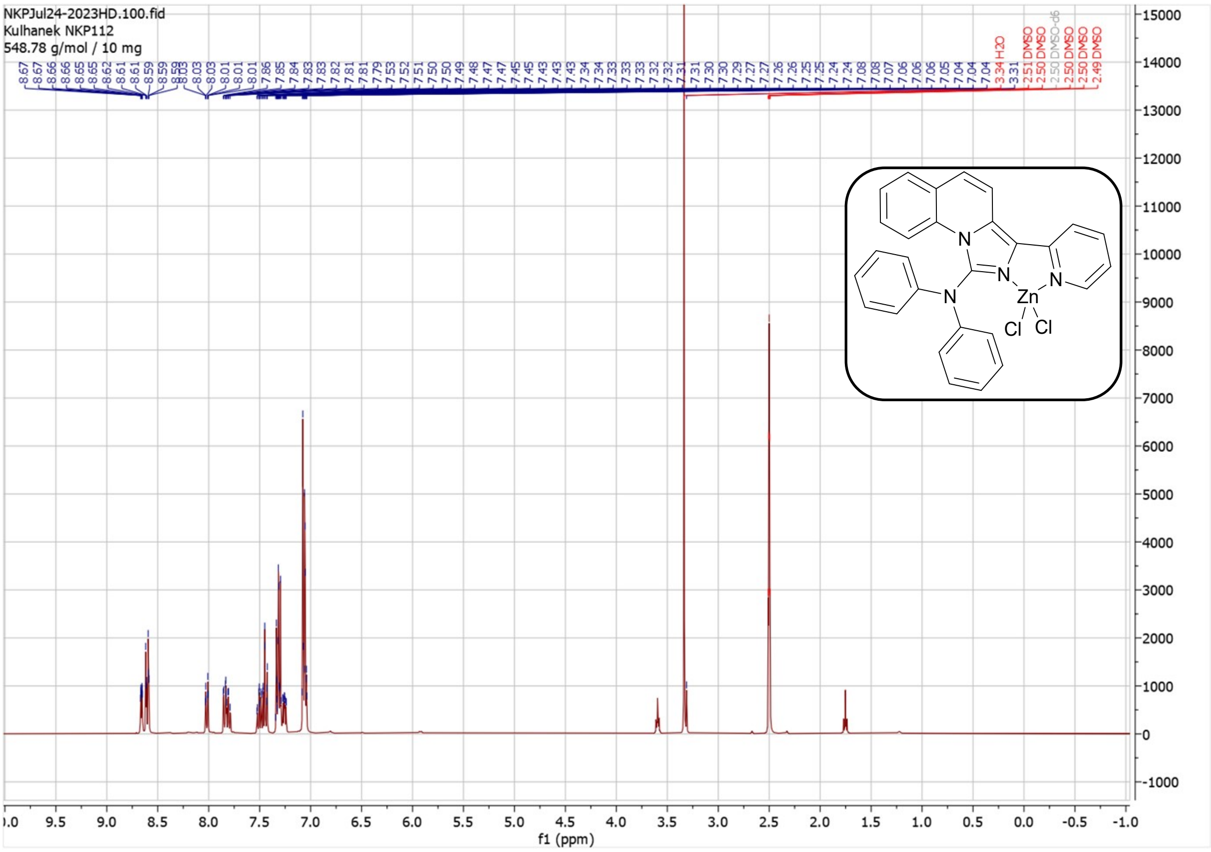
NKPJul24-2023HD.100.fid
Kulhanek NKP112
548.78 g/mol / 10 mg
8.67 8.67 8.66 8.66 8.65 8.65 8.62 8.61 8.61 8.59 8.59 8.59 8.03 8.03 8.01 8.01 7.86 7.85 7.84 7.83 7.83 7.82 7.81 7.79 7.53 7.52 7.51 7.50 7.50 7.49 7.48 7.47 7.45 7.45 7.43 7.43 7.34 7.34 7.33 7.33 7.33 7.32 7.32 7.31 7.31 7.30 7.30 7.29 7.27 7.27 7.26 7.26 7.25 7.25 7.24 7.24 7.08 7.08 7.07 7.06 7.06 7.05 7.04 7.04 7.04
3.34 H2O
3.31
2.51 DMSO
2.50 DMSO
2.50 DMSO-d6
2.50 DMSO
2.50 DMSO
2.49 DMSO
N
N
N
N
Zn
Cl
Cl
f1 (ppm)

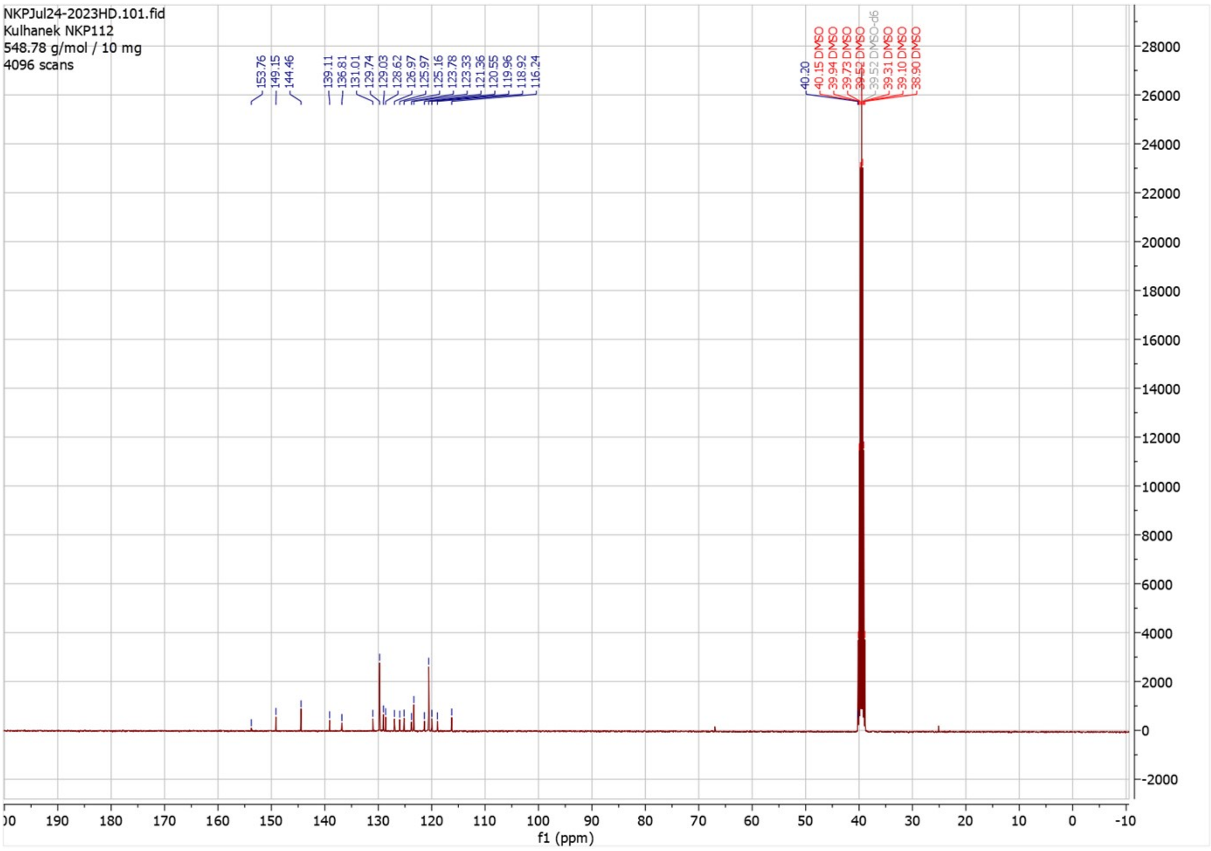
NKPJul24-2023HD.101.fid
Kulhanek NKP112
548.78 g/mol / 10 mg
4096 scans
153.76 149.15 144.46
139.11 136.81 131.01 129.74 129.03 128.62 126.97 125.97 125.16 123.78 123.33 121.36 120.55 119.96 118.92 116.24
40.20
40.15 DMSO
39.94 DMSO
39.73 DMSO
39.52 DMSO
39.52 DMSO-d6
39.31 DMSO
39.10 DMSO
38.90 DMSO
f1 (ppm)

5. XRD data

Diffraction data for all crystals was collected at 100K/125K using φ- and ω-scans on a BRUKER D8 Venture system equipped with dual IμS microfocus sources, a PHOTON100 detector, and an OXFORD CRYOSYSTEMS 700 low temperature system. Mo-Kα radiation with a wavelength of 0.71073 Å and a collimating Quazar multilayer mirror were used. Semi-empirical absorption correction from equivalents was applied using SADABS-2016/2.[3] The structures were solved by direct methods using SHELXT2015.[4] Refinement was performed against F^2 on all data by full-matrix least squares using SHELXL2019/1.[5] All non-hydrogen atoms were refined anisotropically. Aliphatic hydrogen atoms were positioned at geometrically calculated positions and refined using a riding model. The isotropic displacement parameters of all hydrogen atoms were fixed to 1.2x or 1.5x (CH_3 hydrogen atoms) the U_{eq} value of the atoms they are linked to. The crystallographic data have been deposited with the Cambridge Crystallographic Data Centre as CCDC 2284388 (**C1**), 2284389 (**C2**) and 2284387 (**C3**). This data can be obtained free of charge from The Cambridge Crystallographic Data Centre via www. ccdc.cam.ac.uk/structures/.

C1

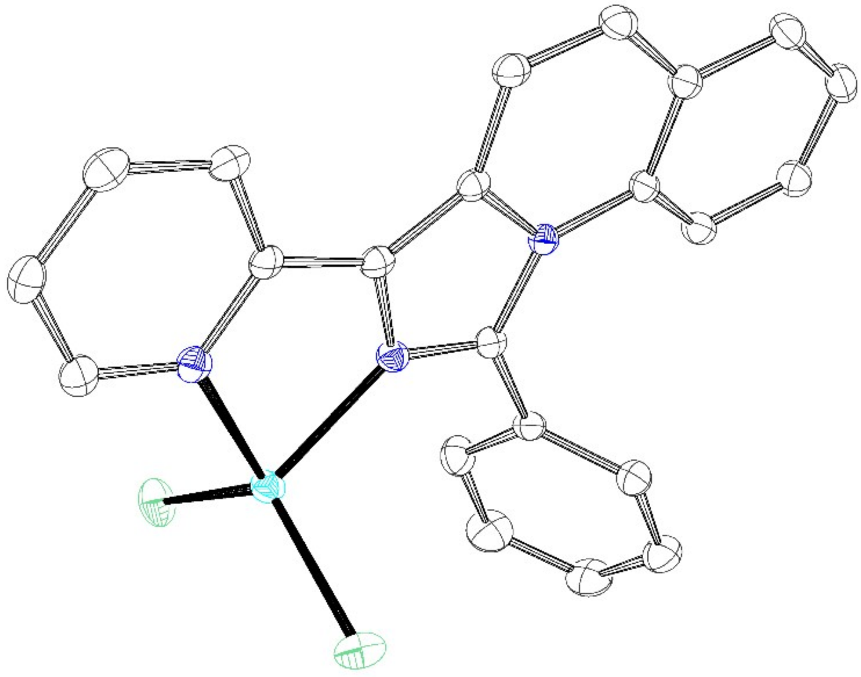

Figure C1 Crystal structure of zinc-complex **C1**. Analysed by XRD measurement, solved by ShelXL and visualized by ORTEP-3. Hydrogen atoms are hidden for better visibility. (white = carbon, blue = nitrogen, red = oxygen, green = chlorine, cyan = zinc, orange = iron and brown = copper)

C2

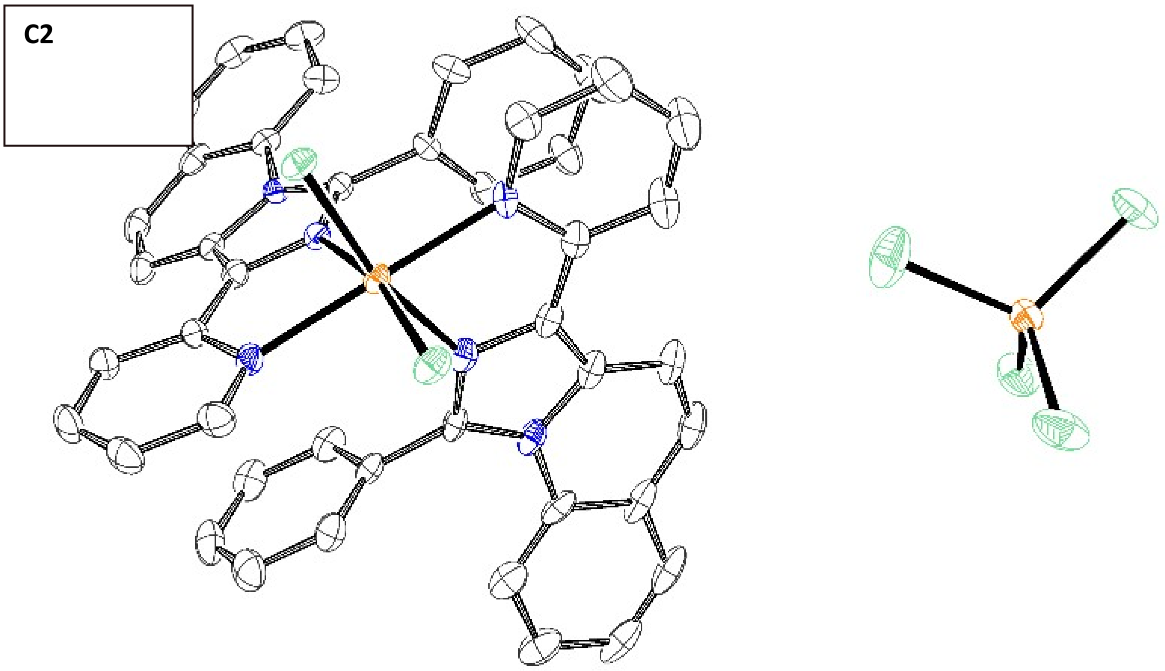

Figure C2 Crystal structure of iron-complex **C2**. Analysed by XRD measurement, solved by ShelXL and visualized by ORTEP-3. Hydrogen atoms are hidden for better visibility. (white = carbon, blue = nitrogen, red = oxygen, green = chlorine, cyan = zinc, orange = iron and brown = copper)

C3

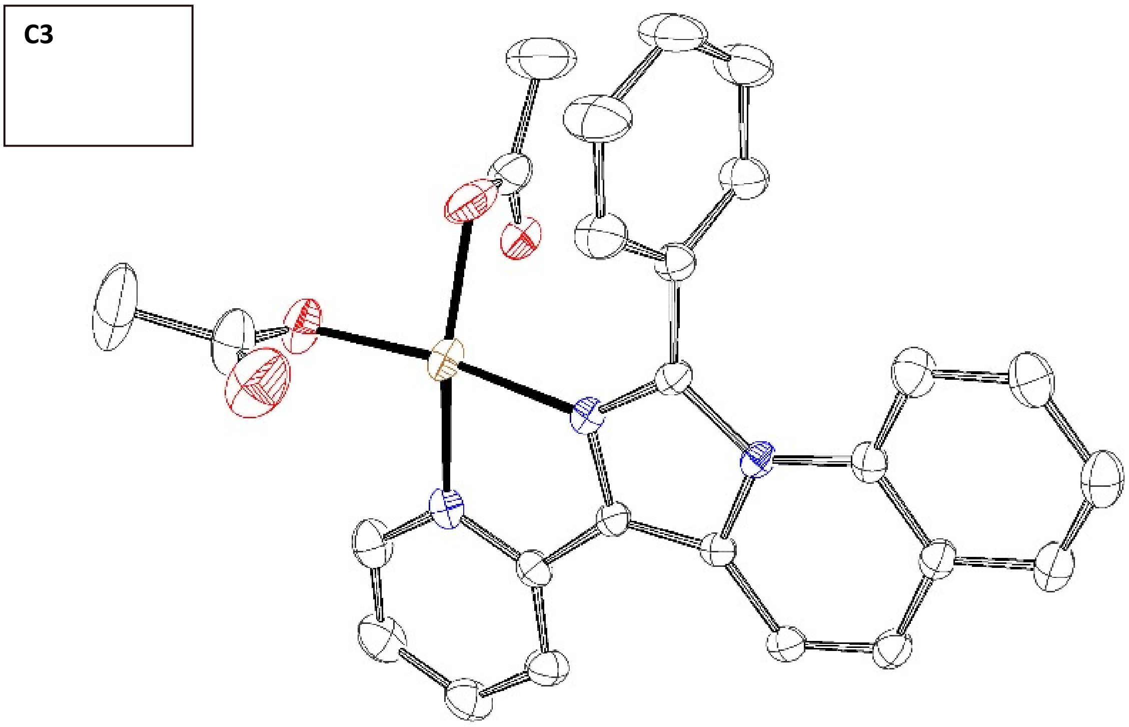

Figure C3 Crystal structure of copper-complex **C3**. Analysed by XRD measurement, solved by ShelXL and visualized by ORTEP-3. Hydrogen atoms are hidden for better visibility. (white = carbon, blue = nitrogen, red = oxygen, green = chlorine, cyan = zinc, orange = iron and brown = copper)

6. Biological Methods

6.1 Antimicrobial Assays

The minimum inhibitory concentration (MIC) of the 24 compounds **5a-i, 6a-b,7a, 8a and C1-8** was determined as previously described with only minor changes.[6,7] Briefly, the cell density of an overnight cultures of *Escherichia coli* ATCC35218 and *Staphylococcus aureus* ATCC25923 was diluted to 5 × 10^5 cells/mL in cation adjusted Mueller Hinton II medium (Becton Dickinson, Sparks, NV, USA) and distributed into 96-well plates. Three 12-point dilution series of rifampicin, tetracycline and gentamicin ranging from 64 - 0.03 µg/mL (all Sigma Aldrich, St. Louis, MO, USA), respectively, were used as positive controls. All tested compounds were dissolved in dimethyl sulfoxide (DMSO, Carl Roth GmbH + Co., Karlsruhe, Germany) and tested in triplicate at the same concentrations as the positive controls. Bacterial suspensions without test compound or positive control were used as negative controls. Medium background was averaged from 5 replicates. Assay incubation was done at 37°C, 180rpm and 85% relative humidity. The turbidity of each well was measured with a microplate spectrophotometer at 600 nm (LUMIstar® Omega BMG Labtech, Ortenberg, Germany). Growth inhibition was calculated relative to the absorption of the controls.

For *S. tritici* MUCL45408, a previously prepared spore solution was used to adjust the assay inoculum to 1 × 10^5 spores/mL in potato dextrose medium (Sigma Aldrich). *Septoria* assay plates were incubated for 4 days at 25°C, 180 rpm and 85% rH. Tebuconazole (Cayman Chemical Company, Ann Arbor, MI, USA) and amphotericin B (Sigma Aldrich) were used as positive control. Cell viability was evaluated via ATP quantification (BacTiter-Glo™, Promega, Walldorf, Germany) according to the manufacturer's instructions.

Mycobacterium tuberculosis ITM-M006710 H37Ra (ATCC 9431) was incubated for 14 days in Middlebrook 7H9 broth (from ThermoFisher Scientific, Walthman, MA, USA) supplemented with 5.6 µg/mL palmitic acid (Sigma Aldrich), 1 mg/mL casitone (ThermoFisher Scientific), 5mg/mL BSA, 4µg/mL catalase (Sigma Aldrich), and 0.2% glycerol at 37°C, 180 rpm and 85% rH. In a first step, the culture was diluted to McFarland standard of 1, before the final inoculum density of a 1 × 10^6 cells/mL was obtained by further dilution with freshly prepared medium. Individual 12-point dilution series of rifampicin, ampicillin and gentamicin (ranging from 64-0.03 µg/mL) were used as positive controls. The assay plates were incubated for 7 days under microaerophilic conditions at 37°C/5% CO_2 (BD gaspak CO_2 system) and read-out was done by ATP quantification (s. above).

The MIC was defined as the minimum concentration where at least 85% growth inhibition relative to the negative control was measured. Relative inhibition for the microtiter turbidity tests (MTT) were calculated as

$$rel.\ inh.\% = 100 * [1- (AU\ sample - AU\ Low)/(AU\ High - AU\ Low)]$$

AU: absorption unit at 600nm; Low: medium blank; High: negative control of maximal growth.

Accordingly, the BacTiter-Glo™ (BTG) assays were calculated as

$$rel.\ inh.\% = 100 * [1- (LU\ sample - LU\ Low)/(LU\ High - LU\ Low)]$$

AU: luminescence unit; Low: medium blank; High: negative control of maximal growth.

For *Mycobacterium tuberculosis* ATCC 35801 a resazurin microtiter assay was carried out as previously described[8] Briefly, assay plates were set up by transferring 10 µl of compounds form a 1:2-fold dilution series in distilled and sterile water to a 96-well plate. The final assay´s concentrations range from 0.39-100 µM and as positive and negative control 5 µM rifampicin and 0.5% DMSO were used. Subsequently a bacterial suspension with *Mycobacterium tuberculosis* with a final OD_{560} of 0.0003 was prepared in Middlebrook 7H9 supplemented with ADC (albumin, dextrose and catalase supplement), 0.2% glycerol and 0.05% Tween 80. 90 µl of the suspension were transferred into each well and plates were sealed with adhesive film. The plates were incubated at 37°C/5% CO_2 for 7 days, 10 µl of 0.025% resazurin was added to each plate and incubated for another 24 h. Fluorescence intensity (EX: 560, EM: 590) of the NADH-dependent metabolite resorufin was measured in a Biotek Cytation 3 reader.[8–10] Data were analyzed with R and its package drc. [11,12]

6.2 Cytotoxicity assays

Calu-3 cells (purchased from Sigma Aldrich (SCC438)) were maintained in Dulbecco's Modified Eagle Medium/ Nutrient Mixture F-12 (DMEM/F-12 GlutaMAX) supplemented with 10% fetal calf serum (FCS) and 1% penicillin/streptomycin (all reagents from ThermoFisher Scientific, Walthman, MA, USA). Cells were grown in an incubator at 37°C and 5% CO_2. The selected compounds and ionomycin (Cayman Chemical, Ann Arbor, MI, USA) as a positive control for cytotoxicity were dissolved in DMSO (10 mM stock solutions) and stored at -20°C. Calu-3 cells were seeded in 96-well plates, grown to 100 % confluence and treated with the compounds, ionomycin (all 100 µM final concentration per well), or DMSO. The DMSO concentration per well was 1%. After 48 h of incubation, cell viability was determined using CellTiter-Glo Luminescent Cell Viability assay (Promega, Walldorf, Germany) according to the manufacturer's instructions. Luminescence was recorded in black 96-well plates in a Synergy H4 microplate reader (Biotek, Waldbronn, Germany). Relative light units (RLU) were normalized to DMSO control set to 100 %. Measurements were performed in triplicates and are expressed as mean ± standard deviation.

HepG2 Assay plates were prepared as described for *Mycobacterium tuberculosis* ATCC 35801, but instead of rifampicin 1 µl 10% Trition X was used as positive control which was added 30 min prior the addition of resazurin. HepG2 cells, an immortal human liver cell line, were brought to a suspension of 2.22×10^5 cells/mL in DMEM supplemented with 10% FCS, 1% sodium pyruvate, and 1% MEM-non-essential amino acids. 90 µL (20,000 cells/well) were distributed to each well of the assay plates. The plates were incubated for 3 days at 37°C/5% CO_2. Trition X was added to designated wells and plates were incubated for 30 min at room temperature. 10 µL 0.025% resazurin was transferred to the plates which were again incubated at 37°C/5% CO_2 for 1 additional hour. Fluorescence intensity as described was measured in a Hidex Reader. Data were analyzed with R and its package drc. [11,12]

7. Bioactivity data

7.1 Antimicrobial data

Table S1 Minimum inhibitory concentration (MIC) of investigated imidazo[1,5-a]quinolines. Ec: *Escherichia coli* ATCC35218, Sa: *Staphylococcus aureus* ATCC33592; Str: *Septoria tritici* MUCL45408, Mtb: *Mycobacterium tuberculosis* H37Ra; MICs given in µM. Controls: RIF: rifampicin; TET: tetracycline; GEN: gentamicin; TEB: tebuconazole; AMB: amphotericin B; AMP: ampicillin. Readout: MTT: microtiter turbidity test @600nm; BTG: BacTiter-Glo™.

**tested n=3**	**MIC (µM)**					
	Ec **ATCC35218 TEM-1 MHII**	***Sa*** **ATCC33592 MRSA MHII**		***Str*** **MUCL45408 QoI res PDB**		***Mtb*** **ATCC25177 H37Ra 7H9-Palmitate**
**RI013**	>196	>196		>196		>196
**RI012**	>200	>200		>200		>200
**RI008**	>199	>199		>199		25
**RI009**	>172	>172		>172		86-43
**RI10S**	>148	>148		>148		1
**RI11S**	>136	>136		>136		34
**RI20S**	>144	>144		>144		1 **-** 0.5
**NKP069**	>198	>198		>198		12-6
**NKP070**	>199	>199		>199		50-25
**NKP071- Fe**	>132	>132		>132		2
**NKP072- Zn**	>140	>140		>140		2
**RI026**	>127	>127		>127		2
**RI021**	>183	>183		>183		46
**NKP057**	>172	>172		>172		5-1
**NKP073**	>155	>155		>155		>155
**NKP104-Cu**	>127	>127		16-8		2
**NKP105- Zn**	> 126	31.5		63		2-1
**NKP106- Zn**	>140	17 - 9		17		2-1
**NKP107- Zn**	>120	>120		>120		8
**NKP108- Zn**	>126	126		126		2-1
**NKP112- Zn**	>117	>117		>117		>117
**ZnCl2**	>470	>470		nd		235
**FeCl3**	>394	>394		nd		>394
**Cu(OAc)2**	nd	nd		nd		43
	controls					
RIF	5	>78	TEB	>0.01	RIF	0.07-0.03
TET	4.5 - 2.25	144 -72	AMB	0.135	AMP	184
GEN	2-1	1-0.5			GEN	4

readout	MTT	MTT		BTG		BTG

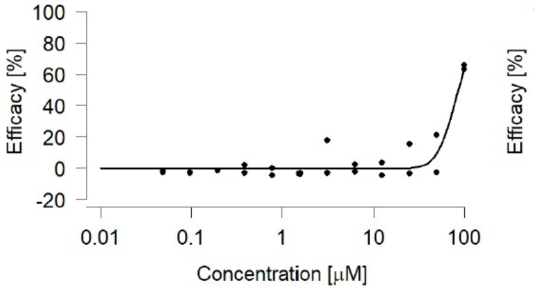

Figure S1. The inhibitory effect of compound **5g** against wild-type *Mycobacterium tuberculosis* strain ATCC 35801. Data was used to calculated IC50/90 values (86.5/136.2μM)

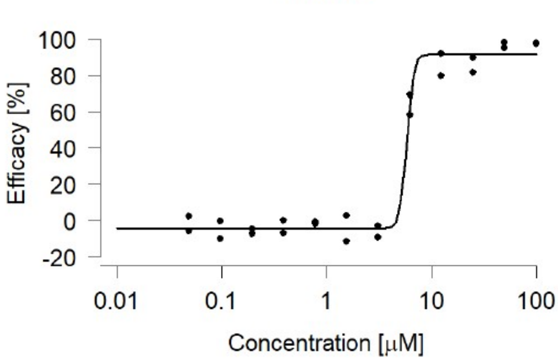

Figure S2. The inhibitory effect of compound **C1** against wild-type *Mycobacterium tuberculosis* strain ATCC 35801. Data was used to calculated IC50/90 values (6/7.7μM)

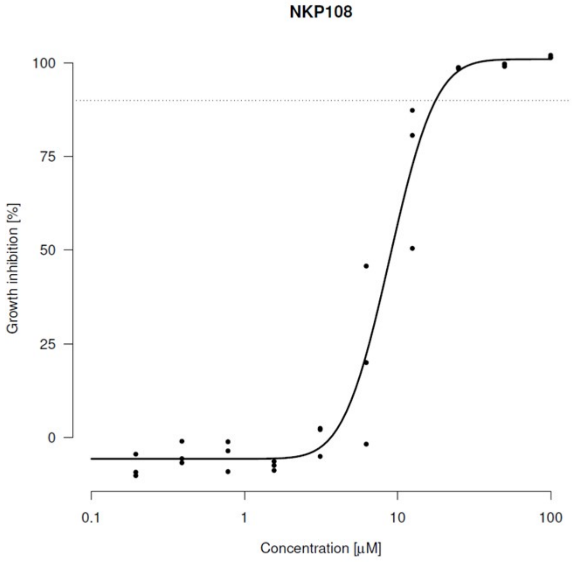

Figure S3. The inhibitory effect of compound **C7** against wild-type *Mycobacterium tuberculosis* strain ATCC 35801. Data was used to calculated IC50/90 values (9.7/17.7μM)

7.2 Cytotoxicity data

Calu-3

Compounds were tested at a single high dose of 100 µM in triplicate. In our tests, compounds causing a cell viability of less than 80% relative to the DMSO control were considered cytotoxic (dotted line).

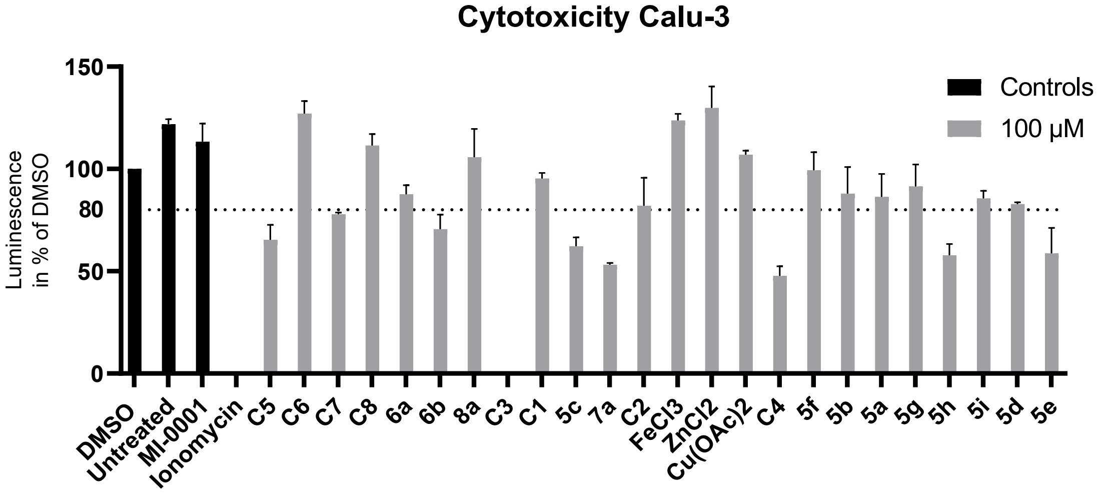

HepG2

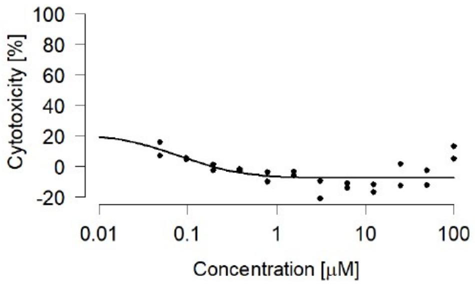

Figure S4. Cytotoxicity effect of compound **5g** human liver cancer cell line HepG2.

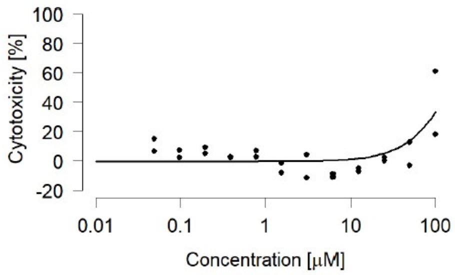

Figure S5. Cytotoxicity effect of compound **C1** human liver cancer cell line HepG2. Data was used to calculated IC50 value (142.2µM)

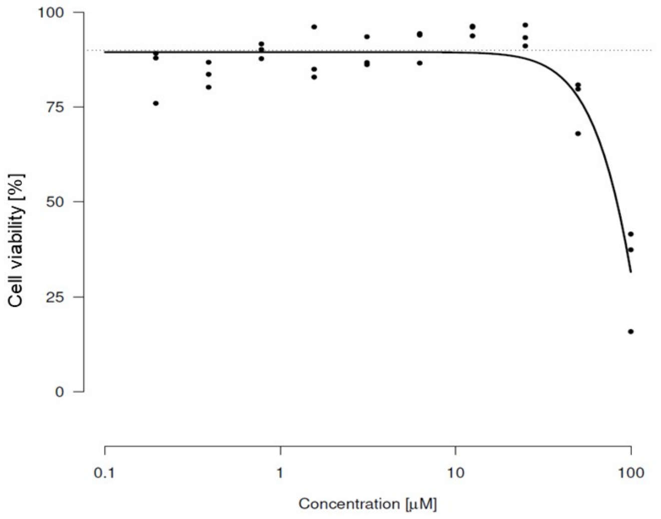

Figure S6. Cytotoxicity effect of compound **C7** human liver cancer cell line HepG2. Data was used to calculated IC50 value (83.3µM)

8. References

1 N. Kulhanek, N. Martin and R. Göttlich, *Eur. J. Org. Chem.*, 2024, **27**. DOI: 10.1002/ejoc.202301007.

2 G. Pelletier and A. B. Charette, *Org. Lett.*, 2013, **15**, 2290–2293.

3 L. Krause, R. Herbst-Irmer, G. M. Sheldrick and D. Stalke, *J. Appl. Cryst.*, 2015, **48**, 3–10.

4 G. M. Sheldrick, *Acta Cryst. A*, 2015, **71**, 3–8.

5 G. M. Sheldrick, *Acta Cryst. C*, 2015, **71**, 3–8.

6 I. D. M. Kresna, Z. G. Wuisan, J.-M. Pohl, U. Mettal, V. L. Otoya, M. Gand, M. Marner, L. L. Otoya, N. Böhringer, A. Vilcinskas and T. F. Schäberle, *J. Nat. Prod.*, 2022, **85**, 888–898.

7 M. Marner, M. A. Patras, M. Kurz, F. Zubeil, F. Förster, S. Schuler, A. Bauer, P. Hammann, A. Vilcinskas, T. F. Schäberle and J. Glaeser, *J. Nat. Prod.*, 2020, **83**, 2607–2617.

8 J.-C. Palomino, A. Martin, M. Camacho, H. Guerra, J. Swings and F. Portaels, *Antimicrob. Agents Chemother.*, 2002, **46**, 2720–2722.

9 J. Rybniker, A. Vocat, C. Sala, P. Busso, F. Pojer, A. Benjak and S. T. Cole, *Nat. Commun.*, 2015, **6**, 7659.

10 I. Kollmorgen, N. Bachmann, M. Dal Molin, C. Degenhart, E. Zent, V. Pareek, U. Koch, J. Rybniker, N. Metzler-Nolte, R. Stoll, B. Klebl, J. E. Bandow and J. Scherkenbeck, *ChemMedChem*, 2023, **18**, e202200631.

11 C. Ritz and J. C. Streibig, *J. Stat. Soft.*, 2005, **12**, 1–22.

12 R Core Team, *R: A Language and Environment for Statistical Computing*, Vienna, Austria, 2023, http://www.R-project.org/, (accessed 9 October 2023).

9. Author Contribution

Michael Marner: Writing original draft (lead), bioactivity assessment (lead)

Niclas Kulhanek: Writing original draft (lead), synthesis (lead), method optimization (lead).

Richard Göttlich: Writing original draft (supporting), project administration (lead).
